# Immunosenescence patterns differ between populations but not between sexes in a long-lived mammal

**DOI:** 10.1038/s41598-017-13686-5

**Published:** 2017-10-20

**Authors:** L. Cheynel, J.-F. Lemaître, J.-M. Gaillard, B. Rey, G. Bourgoin, H. Ferté, M. Jégo, F. Débias, M. Pellerin, L. Jacob, E. Gilot-Fromont

**Affiliations:** 1 Univ Lyon, Université Lyon 1; CNRS, Laboratoire de Biométrie et Biologie Evolutive UMR 5558, F-69622 Villeurbanne, France; 2 Université de Lyon, VetAgro Sup, Marcy-l’Etoile, France; 3 EA 4688 “VECPAR”, UFR Pharmacie, Université de Reims Champagne-Ardenne, Reims, France; 4 Office National de la Chasse et de la Faune Sauvage, Centre National de Recherches Appliquées sur les Cervidés-Sanglier, Bar-le-Duc, France

## Abstract

In animals, physiological mechanisms underlying reproductive and actuarial senescence remain poorly understood. Immunosenescence, the decline in the ability to display an efficient immune response with increasing age, is likely to influence both reproductive and actuarial senescence through increased risk of disease. Evidence for such a link has been reported from laboratory animal models but has been poorly investigated in the wild, where variation in resource acquisitions usually drives life-history trade-offs. We investigated immunosenescence patterns over 7 years in both sexes of two contrasting roe deer populations (*Capreolus capreolus*). We first measured twelve immune markers to obtain a thorough identification of innate and adaptive components of immunity and assessed, from the same individuals, the age-dependent variation observed in parasitic infections. Although the level of innate traits was maintained at old age, the functional innate immune traits declined with increasing age in one of two populations. In both populations, the production of inflammatory markers increased with advancing age. Finally, the adaptive response declined in late adulthood. The increasing parasite burden with age we reported suggests the effective existence of immunosenescence. Age-specific patterns differed between populations but not between sexes, which indicate that habitat quality could shape age-dependent immune phenotype in the wild.

## Introduction

With increasing age, most organisms experience senescence, a process characterised by progressive and irreversible decline in age-specific reproductive success (*i*.*e*. reproductive senescence) and survival (*i*.*e*. actuarial senescence)^1^. Reproductive and actuarial senescence have been repeatedly documented in laboratory animal models^2^, captive^3^ and wild^4^ populations, and senescence appears to be the rule rather than the exception^4,5^ in the living world. However, senescence patterns can be extremely diverse across species^5^ and the reasons for such a high diversity remain poorly understood. It seems that variation in senescence patterns is influenced by environmental conditions^3,6,7^ and often differs between sexes^8,9^. It is thus of particular importance to identify the underlying physiological mechanisms that shape the diversity of senescence patterns between sexes and in relation to environmental conditions.

Many physiological mechanisms potentially underlying senescence have been proposed. They notably include telomere attrition^10^, oxidative stress^11^ and dysregulation of the immune response with increasing age, called immunosenescence^12^. Among these processes, immunosenescence is likely to play an important role on the variation observed in life history traits^13,14^. The deterioration of immune function with age makes individuals more sensitive to infections and diseases, and is thus expected to affect reproductive success and survival under natural conditions^15,16^. Although immunosenescence has been well studied in laboratory conditions, much less is known in the wild because of the difficulty of measuring within-individual changes in the field^17^ (but see^18,19^). For the time being, observations suggest that patterns of age-specific changes in the immune response are similar in wild and laboratory conditions^20^.

In vertebrates, the immune system has two different but complementary components: the innate and the adaptive system. The innate response mostly corresponds to the unspecific cellular response mediated principally by monocytes, natural killer and dendritic cells, while the adaptive response provides an antigen-specific response mediated by T and B lymphocytes. A decline in the adaptive response with increasing age has been reported in humans, laboratory animal models^21^, as well as in the wild (*e*.*g*. collared flycatchers *Ficedula albicollis* ^22^; Soay sheep *Ovis aries* ^20^). On the other hand, the innate component of the immune response such as the inflammatory processes seems to be maintained over ages^21,23^ or even enhanced^20,24^, throughout the lifetime, causing a persistent low-grade inflammation referred to as ‘inflammaging’. Inflammaging has multiple origins and may have many detrimental effects on organisms^25^. These age-related dysregulations and the subsequent reshaping of both components of the immune system can deeply affect the resistance against parasitism and infectious diseases of old individuals as suggested by the consistent increase in parasite burden with age reported so far (*e*.*g*. in Soay sheep^26,27^; in house martins *Delichon urbica* ^28^).

Studies conducted on humans also revealed sex differences in immunosenescence patterns^29^ and generally pointed out that adaptive immune traits decline at a lower rate in women than in men. In the wild, available evidence of sex-differences in immunosenescence patterns are rather indirect (*e*.*g*., age-specific changes in parasitism^26,30^). A better assessment of sex-specific immunosenescence in the wild is thus required. In addition, it could provide important insights to better understand differences in longevity between sexes of many animals such as mammals, where males generally show shorter lifespan^31^ and earlier onsets of senescence^32^ than females. Finally, whether environmental conditions influence immunosenescence profiles remains unknown. In natural conditions, animals are subjected to varying amounts of resources, which likely influence their allocation to immunity^33^. As developing an efficient immune response is a highly energetically demanding process, long-lasting caloric restriction can lead to the suppression of the immune system^34^. Moreover, in mammals, increasing tooth wear with age leads food to be increasingly difficult to acquire (*e*.*g*.^35^ for large herbivores), and even more when resources are limited. This decrease of nutrient input with increasing age could have direct consequences on immune responsiveness and may contribute to accelerate immunosenescence.

We aimed at investigating age-related changes of immune parameters and parasitic load, in males and females of two populations of roe deer *Capreolus capreolus*, subjected to highly different ecological contexts in the wild. In this weakly polygynous ungulate, both actuarial^36^ and reproductive^37^ senescence have been reported, as well as senescence in body mass^38^, home range size^39^ and haematological parameters^40^. Both parasite prevalence and load are higher in old age classes than in young age classes in roe deer^41^, suggesting the existence of some immunosenescence. However, the exact age-specific changes in parasite burden remain to be identified. We thus measured a large set of immune markers, encompassing both innate and adaptive components of the immune response, to obtain a comprehensive picture of the age-related changes in the immune system of known-aged roe deer. We also assessed age-specific patterns in the parasite burden of the same individuals. Based on our current knowledge we predicted: (i) a progressive decline of the adaptive immune component but a stable innate response and an increased inflammatory markers in old individuals, (ii) an earlier and more pronounced immunosenescence in males than in females, (iii) an earlier and sharper immunosenescence in the population with poor and limited food resources (Chizé) than in the population with rich and abundant food resources (Trois-Fontaines), and (iv) an increase in parasitic load with age for individuals of a given sex in a given population.

## Results

Senescence patterns of immune response were analysed from 615 measures on 325 known-aged (from 2 to 16 years) roe deer captured between 2010 and 2016 (Trois-Fontaines n = 166, sex ratio (*i*.*e*. male/female ratio) 0.80:1; Chizé n = 159, sex ratio 0.83:1). Among the 325 studied individuals, 163 were captured once, 80 twice, 47 three times, 25 four times and 10 five or six times. Most individuals captured (76%, *i*.*e*. 467 capture events) were prime-aged adults between 2 and 7 years old, with a sex ratio of 0.86:1 at Trois-Fontaines and of 0.83:1 at Chizé. Old adults (*i*.*e*., older than 7 years) were less numerous (24%, *i*.*e*. 148 capture events), with a more female-biased sex ratio, especially at Chizé (0.57:1 at Trois-Fontaines and 0.26:1 at Chizé). Full information on age- and sex-specific sample size is given in supporting information (Table S1).

As in many studies in wild animal populations, we collected relatively few data from very old individuals (*i*.*e*. older than 10 years, see in supporting information Table S1). This is particularly true for males in Chizé with only two males older than 10 years (captured at 11 and 14 years old). These two individuals had values very different from those of younger individuals, likely due to a degraded physical condition at the end of their life, and these extreme values drove the model selection. We therefore decided to report results without these oldest males. However, we provide results from analyses including these individuals in the supporting information (Table S2). In females at Chizé and in both sexes in Trois-Fontaines, we consistently had at least 3 individuals in each age class.

### Innate immune response

**Figure 1:**
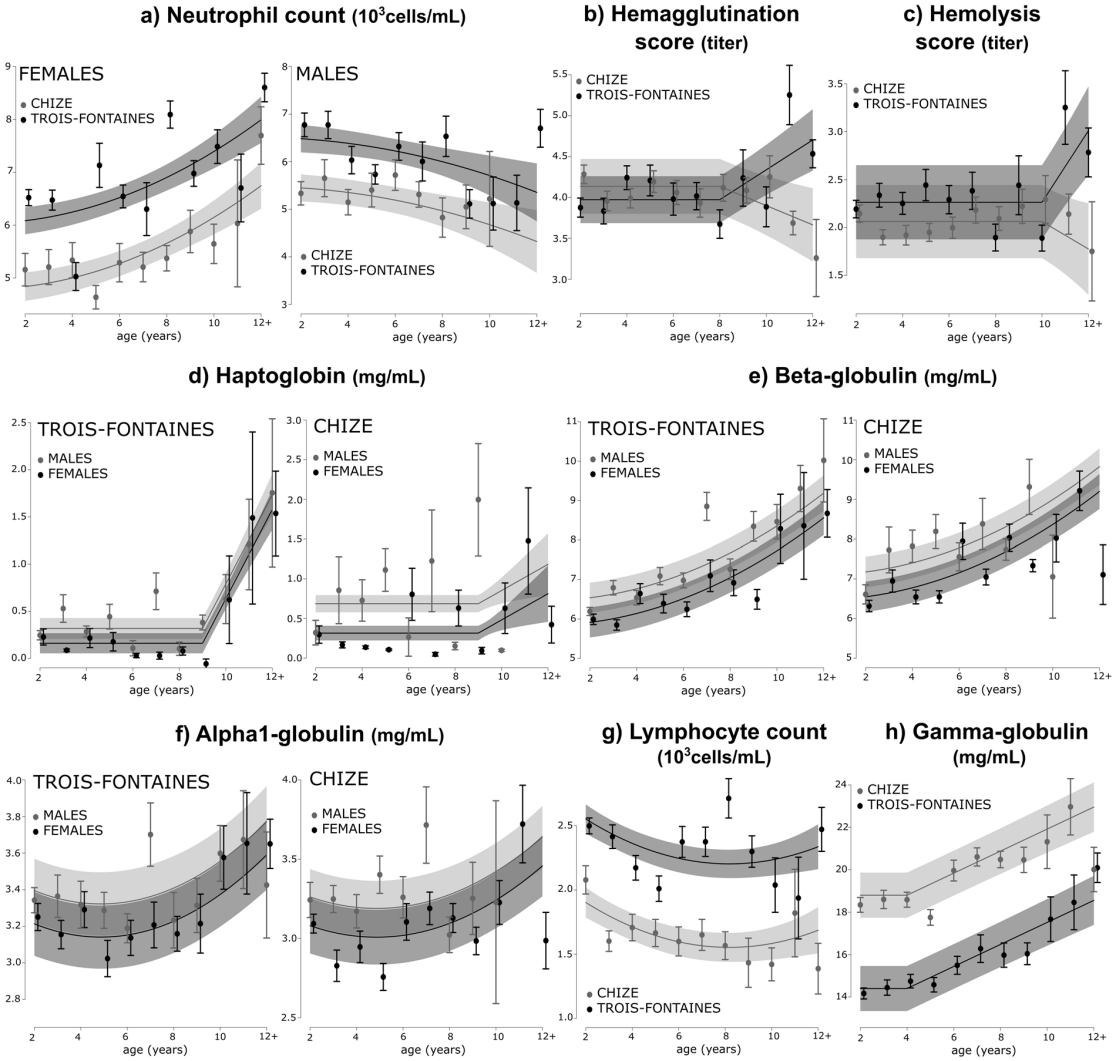
Predicted age-related changes in immune parameters in two populations of roe deer (Trois-Fontaines and Chizé). Plots are based on predicted effects from the selected model for each parameter (see Table 1). The lines represent model predictions and the shaded areas the 95% CIs. The points correspond to the average value per age and the bars correspond to ± Standard Error. All individuals older than 12 years of age were pooled within a “12+” age class.

At two years of age, roe deer of both sexes showed higher levels of neutrophils at Trois-Fontaines than at Chizé (see Table 1 for the predicted values at 2 years old). The neutrophil count increased with age in females in both populations (Table 1; Fig. 1a), whereas it slightly decreased in males in both populations (Table 1; Fig. 1a). Hemagglutination (HA) ability changed with age from 8 years onwards, with marked between-population differences (Table 1; Fig. 1b): it increased at old ages at Trois-Fontaines but decreased at Chizé. Hemolysis (HL) ability showed both similar age-related changes from 10 years and similar between-population differences (Table 1; Fig. 1c). Monocyte, basophil and eosinophil counts all remained constant from 2 years of age onwards in both sexes and populations (Table 1).

**Table 1:** Linear mixed effect models selected for 12 immune parameters.

Immune trait	Best model selected	Age function	Variable	Parameter estimate ± SE	t-value	p	R^2^m	R^2^c	Predicted value at two years			
									♂ TF	♀ TF	♂ CH	♀ CH
INNATE TRAITS												
Neutrophil count	I(age^2)*sex + pop	quadratic	Intercept	6.00 ± 0.25	23.97	***	0.09	0.51	6.25 ± 0.29	6.52 ± 0.15	5.34 ± 0.34	5.14 ± 0.18
			I(age^2)	0.01 ± 0.003	3.97	***						
			Sex (M)	0.57 ± 0.29	1.96	—						
			Pop (CH)	−1.16 ± 0.22	−5.15	***						
			I(age^2): sex (M)	−0.02 ± 0.01	−3.69	***						
Monocyte count	Constant	—	Intercept	0.29 ± 0.09	3.12	—	0.00	0.46	0.30 ± 0.10	0.31 ± 0.10	0.29 ± 0.07	0.30 ± 0.10
Basophil count	Constant	—	Intercept	0.07 ± 0.02	4.33	—	0.00	0.28	0.07 ± 0.02	0.07 ± 0.02	0.07 ± 0.02	0.08 ± 0.01
Eosinophil count	Sex	—	Intercept	0.12 ± 0.01	10.03	***	0.02	0.15	0.07 ± 0.01	0.12 ± 0.02	0.10 ± 0.02	0.11 ± 0.01
			Sex (M)	−0.03 ± 0.01	−2.90	**						
Hemagglutination	Age * pop	threshold (8 years)	Intercept	2.43 ± 0.65	3.76	***	0.01	0.44	4.03 ± 0.33	4.03 ± 0.25	4.26 ± 0.35	3.98 ± 0.36
			Age	0.19 ± 0.07	2.85	**						
			Pop (CH)	2.67 ± 0.88	3.04	**						
			Age: Pop	−0.32 ± 0.10	−3.01	**						
Hemolysis	Age * pop	threshold (10 years)	Intercept	−1.52 ± 1.31	−1.17	—	0.01	0.62	2.24 ± 0.41	2.35 ± 0.37	2.10 ± 0.40	1.97 ± 0.38
			Age	0.38 ± 0.12	3.07	**						
			Pop (CH)	5.11 ± 2.06	2.48	*						
			Age: Pop	−0.53 ± 0.20	−2.57	*						
INFLAMMATORY TRAITS												
Alpha1-globulin	Age + I(age^2) + pop + sex	quadratic	Intercept	3.35 ± 0.19	17.45	***	0.04	0.5	3.35 ± 0.20	3.11 ± 0.19	3.24 ± 0.16	2.94 ± 0.15
			Age	−0.09 ± 0.03	−2.54	*						
			I(age^2)	0.01 ± 0.003	3.29	**						
			Pop (CH)	−0.13 ± 0.05	−2.76	**						
			Sex (M)	0.18 ± 0.05	3.79	***						
Alpha2-globulin	Constant	—	Intercept	5.79 ± 0.30	19.62	−	0.00	0.26	5.63 ± 0.30	5.84 ± 0.34	5.66 ± 0.33	5.88 ± 0.29
Beta-globulin	I(age^2) + sex + pop	quadratic	Intercept	5.84 ± 0.38	15.22	***	0.12	0.43	6.20 ± 0.54	5.73 ± 0.36	7.12 ± 0.51	6.41 ± 0.41
			I(age^2)	0.02 ± 0.002	8.71	***						
			Sex (M)	0.63 ± 0.16	3.81	***						
			Pop (CH)	0.64 ± 0.16	3.87	***						
Haptoglobin	Age * pop + sex	threshold (9 years)	Intercept	−4.24 ± 0.74	−5.71	***	0.07	0.15	0.34 ± 0.10	0.15 ± 0.06	0.69 ± 0.14	0.30 ± 0.11
			Age	0.48 ± 0.08	6.04	***						
			Pop (CH)	3.18 ± 1.20	2.66	**						
			Sex (M)	0.25 ± 0.08	3.05	**						
			Age: Pop	−0.33 ± 0.13	−2.50	*						
ADAPTIVE TRAITS												
Lymphocyte count	Age + I(age^2) + pop	quadratic	Intercept	2.81 ± 0.17	16.49	***	0.14	0.37	2.25 ± 0.14	2.42 ± 0.13	1.90 ± 0.17	1.89 ± 0.15
			Age	−0.15 ± 0.05	−2.76	**						
			I(age^2)	0.01 ± 0.004	2.12	*						
			Pop (CH)	−0.65 ± 0.08	−8.27	***						
Gamma-globulin	Age + pop	threshold (4 years)	Intercept	12.32 ± 1.14	10.5	***	0.21	0.65	13.86 ± 0.90	13.54 ± 0.93	18.55 ± 1.66	18.43 ± 1.57
			Age	0.52 ± 0.08	6.79	***						
			Pop (CH)	4.40 ± 0.39	11.43	***						

The effect of different age functions (factor, linear, threshold, quadratic), of sex (F: Female, M: Male), of population (TF: Trois-Fontaines, CH: Chizé), with all two and three-way interactions between them, were tested. All models included individual identity, the year of capture and the cohort of individuals as random effects. When the age threshold model was selected, we have indicated the age at which the parameter begins to vary, and the “Parameter estimate” of the age function is the slope of the variation with age after the threshold age. Statistical significance is represented by *for p < 0.05, **for p < 0.01 and ***for p < 0.001. R^2^m and R^2^c are the marginal and conditional variance of the model, respectively. Values are presented ± Standard Error.

### Inflammatory markers

At two years of age, males had higher levels of haptoglobin than females in both populations, with higher values at Chizé than at Trois-Fontaines in both sexes (see Predicted values at 2 years in Table 1). Age-related changes in haptoglobin concentration differed between the populations (Table 1; Fig. 1d). In both sexes at Trois-Fontaines, haptoglobin levels increased markedly from 9 years of age (Fig. 1d). Likewise, haptoglobin levels also increased from 9 years of age in both sexes at Chizé, but displayed quite lower values (Fig. 1d). Beta-globulin levels varied in relation to both population and sex, but consistently increased from 2 years of age (see population effect estimates in Table 1, Fig. 1e). Levels of alpha1-globulin increased with advancing age in both sexes of the two populations (Table 1; Fig. 1f). Finally, alpha2-globulin levels remained constant whatever the sex and the population considered (Table 1).

### Adaptive response

At two years of age, roe deer had lower levels of lymphocytes at Chizé than at Trois-Fontaines (see Predicted values at 2 years in Table 1). Lymphocyte counts declined with increasing age in both sexes of the two populations (Table 1; Fig. 1g). On the contrary, at two years of age, roe deer had higher gamma-globulins levels at Chizé than at Trois-Fontaines (see Predicted values at 2 years in Table 1). Gamma-globulins concentration increased from 4 years of age in both populations (Table 1; Fig. 1h).

### Parasitism

**Figure 2:**
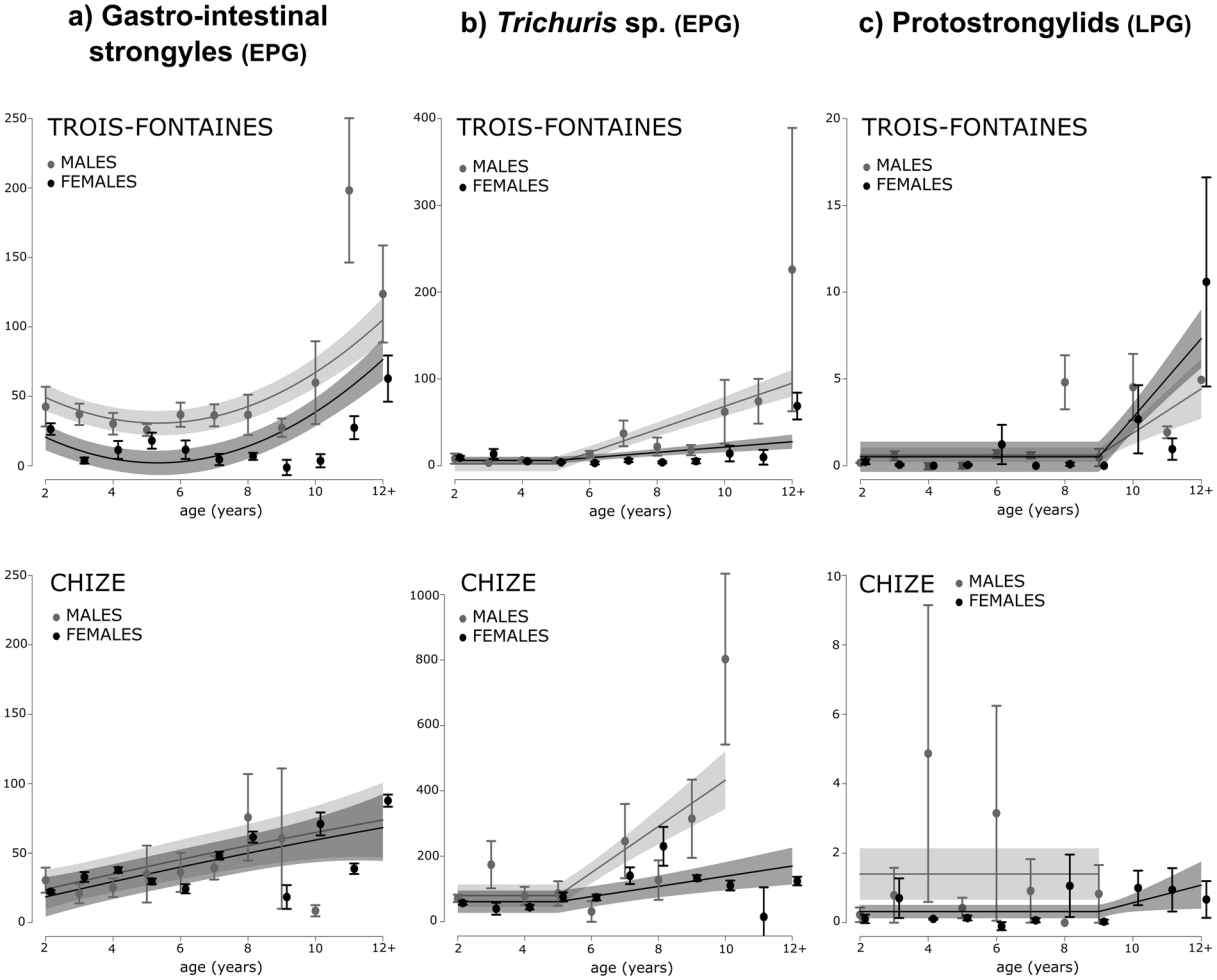
Predicted age-related changes in parasite abundance in two roe deer populations (Trois-Fontaines and Chizé). Plots are based on predicted effects from the selected model for each parameter (see Table 2). The lines represent model predictions and the shaded areas the 95% CIs. The points correspond to the average value per age and the bars correspond to ± Standard Error. All individuals older than 12 years of age were pooled in a “12+” age class.

The abundance of gastro-intestinal strongyles increased with age in both sexes of the two populations (Table 2; Fig. 2a). The abundance of *Trichuris* sp. markedly increased from 5 years of age in males of the two populations (Table 2; Fig. 2b). The abundance of *Trichuris* sp. also increased from 5 years of age in females of the two populations, but more slightly (Fig. 2b). The abundance of protostrongylids increased from 9 years of age in both sexes at Trois-Fontaines and in females at Chizé (Table 2; Fig. 2c). In Chizé males, the abundance of protostrongylids remained constant with age, but we did not have data in males older than 9 years (Fig. 2c). Finally, we did not find any age-related changes in coccidia abundance in either sex of the two populations (Table 2).

**Table 2:** Linear mixed effect models selected for 4 parasite abundances.

Parasitic trait	Best model selected	Age function	Variable	Parameter estimate ± SE	t-value	p	R^2^m	R^2^c	Predicted value at two years			
									♂TF	♀TF	♂CH	♀CH
Gastro-intestinal strongyles	(age + I(age^2)) * pop + sex	quadratic	Intercept	45.63 ± 16.02	2.85	**	0.06	0.77	34.99 ± 10.81	15.05 ± 4.93	34.48 ± 10.61	22.01 ± 16.35
			Age	−15.30 ± 5.16	−2.97	**						
			I(age^2)	1.55 ± 0.42	3.72	***						
			Pop (CH)	−48.27 ± 21.40	−2.26	*						
			Sex (M)	19.86 ± 8.27	2.4	*						
			Age: Pop (CH)	20.35 ± 7.76	2.62	**						
			I(age^2): Pop (CH)	−1.42 ± 0.64	−2.23	*						
*Trichuris* sp.	age * pop * sex	threshold (5 years)	Intercept	−20.85 ± 40.58	−0.51	—	0.15	0.18	7.97 ± 4.75	7.35 ± 3.40	81.42 ± 32.81	86.40 ± 29.43
			Age	4.19 ± 5.66	0.74	—						
			Pop (CH)	−4.21 ± 55.94	−0.08	—						
			Sex (M)	−35.95 ± 60.37	−0.60	—						
			Age: Pop (CH)	10.28 ± 8.02	1.28	—						
			Age: Sex (M)	8.13 ± 9.20	0.88	—						
			Pop (CH): Sex (M)	−222.44 ± 99.69	−2.23	*						
			Age: Pop (CH): Sex (M)	49.66 ± 16.08	3.09	**						
Protostrongylids	age * pop * sex	threshold (9 years)	Intercept	−24.67 ± 4.09	−6.04	***	0.13	0.15	0.17 ± 0.02	0.41 ± 0.37	1.40 ± 0.74	0.38 ± 0.18
			Age	2.76 ± 0.44	6.3	***						
			Pop (CH)	22.83 ± 6.14	3.72	***						
			Sex (M)	17.84 ± 8.22	2.17	*						
			Age: Pop (CH)	−2.52 ± 0.66	−3.82	***						
			Age: Sex (M)	−1.94 ± 0.90	−2.17	*						
			Pop (CH): Sex (M)	−213.75 ± 38.07	−5.61	***						
			Age: Pop (CH): Sex (M)	23.83 ± 4.22	5.65	***						
Coccidia	constant	—	Intercept	2560.00 ± 2296	1.12	—	0.00	0.98	233.38 ± 194.70	244.10 ± 202.30	26.07 ± 13.75	27.48 ± 8.86

The effect of different age functions (factor, linear, threshold, quadratic), of sex (F: Female, M: Male), of population (TF: Trois-Fontaines, CH: Chizé), with all two and three-way interactions between them, were tested. All models included individual identity, the year of capture and the cohort of individuals as random effects. When the age threshold model was selected, we have indicated the age at which the parameter begins to vary, and the “Parameter estimate” of the age function is the slope of the variation with age after the threshold age. Statistical significance is represented by *for p < 0.05, **for p < 0.01 and ***for p < 0.001. R^2^m and R^2^c are the marginal and conditional variance of the model, respectively. Values are presented ± Standard Error.

## Discussion

Our results provide the first assessment of age-related and sex-specific changes of a large array of immune parameters (12 markers) and parasitic load (4 parasitic traits) in two contrasted populations of a wild vertebrate. Our findings demonstrate that both components of the immune response do show senescence, as characterized by marked changes with increasing age in many measured immune parameters. We also observed an age-specific increase in the parasitic burden of both sexes in the two populations studied.

Different patterns of senescence occurred between sexes and populations (see also^27^). As some individuals may experience specific conditions associated with particular immune responses, variation in the health status of roe deer provides a first plausible explanation for these different patterns. Although the clinical examination of roe deer did not reveal any sign of pathology at the time of capture, some individuals might have experienced inflammatory state, dehydration or malnutrition that could have influenced the measures^42^.

The cellular innate immune traits (*i*.*e*. monocyte, eosinophil and basophil counts) did not decline with increasing age in both populations. The neutrophil counts even increased in females in both populations. We only found a decline in the neutrophil count in males of the two populations. While the stability in the number of innate immune cells over age has also been reported in humans^21^ and in wild Tree swallows *Tachycineta bicolor* ^23^, this does not imply that the performance of the innate immune system remains stable over the individual life course. The increase in neutrophil counts observed in females of the two populations could be a way to compensate^25^ not only for the decline of the adaptive response often occurring with increasing age, but also for the reduction of intrinsic functional activity of leukocytes. Indeed, the phagocytic ability of neutrophils or monocytes often decreases in old individuals^43^. The decline in adaptive response (*i*.*e*. lymphocyte count) is actually observed in the two populations. We also brought evidence of an increase of inflammatory markers with age (*i*.*e*. haptoglobin, alpha1-globulin and beta-globulin) in both sexes of the two populations. The increase of inflammatory markers has been observed in old humans^24,44^ and in Soay sheep older than 7 years^20^. It suggests that a progressive dysregulation of the inflammatory response occurs at old ages, leading to an increase in the production of related inflammatory products. While an acute and transient inflammation allows neutralizing invading pathogens and facilitates repair and turn-over of injury tissues, chronic inflammation causes tissue degeneration affecting body condition and weakening individuals^25^. Circulating pro-inflammatory molecules are considered as strong predictors of age-related morbidity and mortality^44^. This increased inflammatory state could also be linked to the decline in survival in the older age classes previously reported in roe deer^36^.

The decline in adaptive response at old ages we report on roe deer has previously been documented in humans^21^, mice^45^ and in two vertebrates in the wild (Tree swallows^23^; and Soay sheep^20^). Although our method does not discriminate T and B lymphocytes, a decline in naïve T lymphocytes is expected to occur with ageing, and could thus possibly occur in old roe deer. Such decline may be due to the involution of the thymus, occurring quite early in life in all vertebrates and leading to a decline of the naïve T cell production^46^. On the other hand, we observed an increase of the gamma-globulin concentration with age in both sexes of the two populations. Thus, the capacity of B lymphocytes to produce gamma-globulins seems to be maintained, or maybe even increased, with age.

Our findings attest the existence of immunosenescence in roe deer in the wild. In this species, actuarial senescence begins at around 7 and 8 years of age in the two studied populations^36,47^, reproductive senescence at around 11 and 12 years of age for females^37^ and body mass senescence between 7 to 9 years of age^38^. However, some haematological parameters like creatinine or albumin start to decline earlier in life^39^, and some immune traits (*e*.*g*. beta-globulin, gamma-globulin, lymphocyte count) begin to show age-related changes prior to 8 years of age. Haematological and biochemical traits, which show quite early senescence, could play a crucial role in the senescence of body mass or female reproduction, which both start at older ages. The assessment of a decline of physiological traits with increasing age could also provide a reliable measure of individual body condition and allow more accurate detection of the onset of the decline in physiological mechanisms, ultimately leading to the death of individuals. The assessment of age-related changes in parasitic burden, which occurred in both roe deer populations, also suggests that immunosenescence may impact individual’s physiological condition. Indeed, in both sexes of the two populations, at least two out of four parasitic traits involving digestive or pulmonary nematodes increased with age. Like the immune traits, the increase of gastrointestinal strongyles and *Trichuris* sp. started quite early in life, prior to 8 years of age in both sexes of the two populations, which suggests that immune functions are effectively declining with increasing age. A covariation between immune and parasite traits might involve a direct functional relationship. Thus, an immune trait may indicate the strength of the parasite exposure (positive association) or resistance (negative association) to infection. However, a detailed analysis of the relationships between parasitism and immune profile is beyond the scope of this work because it is required to account for likely confounding effects of yearly variation in environmental conditions and the past parasitic status of individual roe deer to obtain firm results. The descriptive analysis of the relationships between immune and parasite trait we reported in Supplementary Information should thus be interpreted with great caution.

Importantly, our study also allows detecting differences in age-related changes in immunity and parasitism between the two populations. Although we cannot identify the exact driver of this between-population difference, environmental conditions offer a potential candidate. The marked differences in food resources between these populations have been previously shown to influence strongly body size^48^ and demographic traits^49^ and could likely affect the immune phenotype as well. Two years-old roe deer had higher levels of adaptive humoral response (antibodies) at Chizé than at Trois-Fontaines, which indicates a higher allocation to the adaptive response (see also^50^). As the adaptive response is likely to be less energetically costly than innate immune response^51^, the higher adaptive response observed at Chizé matches the predicted response to low resource availability in an environment with predictable pathogens. Males at Chizé also had high values of inflammatory proteins at two years of age (haptoglobin). Then, statistically significant interactive effects between age and population occurred in humoral innate (*i.e.* hemagglutination and hemolysis) and inflammatory (*i.e.* haptoglobin) markers: these three traits markedly increased with age in Trois-Fontaines, but increased much less (or even decreased) at Chizé. One cannot exclude that the two studied populations may differ in several aspects, such as genetic background and pathogenic environment. However, the latter was found to be relatively similar^40^. In contrast, environmental conditions (especially nutritional resources) clearly differ between both sites, and this difference is expected to affect both life history and immunity^52^. In the wild, organisms may have access to a limited amount of resources, leading them to share the energy gained from these resources among growth, reproduction, and soma-maintenance^33,53^. As the development, maintenance and use of efficient immune responses are costly and require nutrients^34^, poorer nutritional resources and starvation experienced more often by roe deer at Chizé could account for the between-population difference observed in their immunosenescence patterns.

Contrary to studies in humans^29^, we did not find clear evidence of sex differences in immunosenescence patterns. While immune differences between sexes occur from early life onwards^54^, patterns of age-related changes appear to be quite similar between sexes. Thus, the decline in immune response with increasing age is probably not an underlying cause of observed sex differences in longevity, at least in the two roe deer populations we studied. However, in these populations, roe deer males exhibited higher levels of parasitism than females and a steeper increase in parasite burden with age, in accordance with previous studies^55^. As roe deer males and females do not segregate spatially^56^, the exposure to parasites is expected to be similar in both sexes. The increase of parasite burden with age might involve a weaker immune ability of males compared to females, which may have not been detected.

Our first assessment of age-specific variation in immune traits and parasitism in two populations of roe deer offers a comprehensive view of age variation in physiological performance in the wild, and appeals for future studies to uncover the demographic consequences of this pattern. A key challenge would thus be to investigate how the age-related changes we highlighted in immunological and parasitological traits relate to both reproductive and actuarial senescence patterns.

## Methods

### Ethics

The protocol of capture and blood sampling of roe deer under the authority of the Office National de la Chasse et de la Faune Sauvage (ONCFS) was approved by the Director of Food, Agriculture and Forest (Prefectoral order 2009–14 from Paris). The land manager of both sites, the Office National des Forêts (ONF), permitted the study of the populations (Partnership Convention ONCFS-ONF dated 2005-12-23). All experiments were performed in accordance with guidelines and regulations of the Ethical Committee of Lyon 1 University (project DR2014-09, June 5, 2014).

### Study population

We focused on two populations of roe deer in the wild, ‘Trois-Fontaines’ and ‘Chizé’. Both sites are enclosed forests. Trois-Fontaines (1,360 ha), located in north-eastern France (48°43′N, 4°55′E), has a continental climate characterized by cold winters and warm rainy summers. This site has rich soils and offers habitat of high quality to roe deer. In contrast, in Chizé (2,614 ha) located in western France (46°50′N, 0°25′W), the climate is temperate oceanic with Mediterranean influences. This site presents low productivity due to poor quality soils and frequent summer droughts^57^ and thus offers a relatively poor habitat to roe deer.

Roe deer from these two populations have been monitored using a long-term Capture-Mark-Recapture program since 1975 and 1977 for Trois-Fontaines and Chizé, respectively. Every year and for each site, 10-12 days of capture are organized between December and March (see^36^ for details about the capture sessions). Once an individual is captured, its sex and body mass (to the nearest 50 g) are recorded and a basic clinical examination is performed. In this study, we only used data from known-age individuals (*i*.*e*., caught during their year of birth, identified using tooth eruption patterns^58^). Since 2010, we collected blood samples from the jugular vein (up to 20 mL for a 20 kg roe deer). Whole blood was EDTA-preserved for cell count and serum was extracted for other measures. We also collected fecal samples. After sampling, roe deer were released at the location of capture within a couple of hours. Samples were received at the laboratory within 48 hours after sampling and analysed within 4 hours after reception.

### Characterization of immune phenotype

We measured a set of 12 immune parameters in order to depict both the innate and the adaptive responses. These two responses are represented by both humoral and cell-mediated components^59^.

First we assessed innate cellular immunity by counting total white blood cells (WBC, in 10^3^ cells/mL), which is considered as a proxy of the allocation to immunity, using a Konelab 30i automaton (Fisher Thermo Scientific, Cergy-Pontoise, France). We also determined the composition of the WBC population (five different cell types), based on the identification of the first hundred WBC in Wright-Giemsa-stained blood smears^60^. Among these, neutrophils and monocytes are phagocytes involved in the innate response. Basophils, which are quite rare, play a key role against macroparasites such as ticks^61^ while eosinophils are associated with defence against internal parasites and inflammatory response. Total white blood cells (WBC) and neutrophil count were highly correlated (r = 0.91, see pairwise correlations displayed in Table S3), because neutrophils represent the majority of white blood cells (between 60 and 80% of the total WBC). However, we reported estimates of the relationship between WBC and age in supporting information (Table S4) to allow future potential comparisons or meta-analyses across species because WBC is a commonly used marked in immunosenescence studies. Finally, lymphocytes represented the adaptive cellular part of immunity (see below).

Innate humoral immunity was assessed by measuring the circulating levels of natural antibodies (NAbs) and the complement-mediated cell lysis activity following the hemagglutination-hemolysis (HAHL) assay^62^ previously performed on roe deer^50^. In this assay, the HA score (titer) measures the ability of NAbs to agglutinate exogenous cells and provides a proxy of the NAbs concentration, and HL score measures the ability of the complement system to cause hemolysis.

Innate humoral immunity also includes numerous proteins involved in acute and chronic inflammatory processes. We thus measured alpha1-globulins, alpha2-globulins and betaglobulins; globulins fractions including several acute phase proteins of the inflammatory response^63^. Total protein content (in g/L) was first assessed by refractometry followed by automatic agarose gel electrophoresis (HYDRASYS, Sebia, Evry, France) that separates albumin and the 4 fractions of globulins (α1, α2, β, and γ). We also measured the specific level of haptoglobin (in mg/mL), a protein that belongs to alpha2-globulin fraction synthesized in case of chronic infection or inflammation. Haptoglobin analyses were performed on a Konelab 30i automaton (Fisher Thermo Scientific, Cergy-Pontoise, France) using phase Haptoglobin assay (Tridelta Development LTD, County Kildare, Ireland) chromogenic kit.

The humoral component of the adaptive immunity response was assessed by measuring the concentration of gamma-globulins (see above for details about the electrophoresis protocol), or immunoglobulins, which represent the majority of circulating antibodies. The cellular component of adaptive immunity was assessed by lymphocyte counts including both T and B cells, B cells being particularly involved in the production of antibodies.

### Measures of parasitic load

We investigated fecal propagule counts of parasites frequently occurring in roe deer^41^: nematodes parasite from the lung (protostrongylids), from the digestive tract (gastro-intestinal strongyles, *Trichuris* sp.), and coccidia (*Eimeria* sp., Protozoa). The McMaster protocol^64^ was used for the count of gastrointestinal nematode eggs/g (EPG) and coccidian oocysts/g (OPG); and the Baermann fecal technique^65^ for the count of first stage larvae of pulmonary nematode (protostrongylids, in larvae/g, LPG). We previously provided evidence that egg counts in faeces allow a reliable estimate of the number of parasites in roe deer from the studied populations during the capture period^41^.

### Statistical analysis

In all the analyses, we only included known-aged male and female roe deer from 2 years of age onwards, which corresponds to the minimum age of reproduction in roe deer^66,67^. We pooled individuals aged 12 years and older in a single age class ‘12+’, as supported by survival analyses^68^. This class included 3 males (aged 12, 12 and 13) and 10 females (8 aged 12–13; 2 aged 15 and 16) at Trois-Fontaines. At Chizé, 1 male (14 years old) and 6 females (aged 12–13) were the oldest.

To assess age-specific changes in immunological parameters and in parasitic load, analyses were performed using linear mixed-effect models (LMMs). Individual identity was included as a random effect, to avoid pseudo-replication issues^69^ and to account for confounding effects of individual heterogeneity when assessing age-specific changes^70^. The cohort was also included as a random effect to take into account the marked differences roe deer faced during early life in response to high variation in environmental conditions among years^71^, which could influence senescence rates^72^. Although, there is no clear evidence of any density response in a life history trait during the study period^49^, we controlled for between-year variation in population density that could potentially influence immune or parasitic traits markers. We thus included the year of capture as a random effect. Each immune or parasitic trait was analysed as a response variable. Age, sex and population were entered as explanatory variables, with all two and three-way interactions between them. Four types of age functions were tested separately and compared: full age dependence (11 separated age classes from 2 to 12+), linear, quadratic, or threshold. For the “threshold model” model, the threshold was determined by maximum likelihood estimation over a grid of values between 3 and 11 years of age (see^75^ and Fig. S1 for the deviance profile). The full list of fitted models is provided in supporting information (Table S5 for immune traits, Table S6 for parasitic traits). To select the best model of age-specific variation in each immune or parasitic variable, we used a model selection procedure based on the Akaike Information Criterion (AIC^73^). For each trait, we retained the model with the lowest AIC, and when the difference of AICs between competing models was less than 2, we retained the simplest model to satisfy parsimony rules^74^. In addition, we calculated the AIC weights (w_i_) to measure the relative likelihood of each model to be the best among the set of fitted models. The normality of the residuals for the selected model was tested (Shapiro–Wilk normality test) and visually assessed with histograms. Goodness-of-fit was assessed through calculating conditional (total variance explained by the best supported model) and marginal (variance explained by fixed effects alone) R^2^ formulations (Table 1, Table 2) and standard residual plot techniques^76^. From the selected model, we then estimated the value of the explained variable (±SE) at two years of age (Table 1, Table 2). Model selection and results of similar analyses performed separately per sex and population (along with additional fixed effect of body mass) are provided in supporting information (Table S7).

When analysing the parasite load, we considered for each parasite its presence (individual presence/absence, analysed with a GLMM with a binomial error), intensity (mean faecal egg or oocyst counts in infested hosts) and abundance (mean faecal egg or oocyst counts per host). Some models could not be fitted to intensity or presence data because of too little data that lead to optimization problems over which classical numerical methods failed to converge. As abundance encompasses both presence and intensity, we only present results on abundance.

To assess the relationships between the 12 immune and the 4 parasite traits, analyses were performed using linear mixed-effect models (LMMs). Each immune trait was analysed as a function of parasite load, population and the interaction parasite load*population, considering 4 different parasite groups. Models included individual identity as a random effect. This descriptive analysis is provided in Supplementary Information (Table S8).

Blood sampling started in 2010 in both study sites and the age at death was known for 37% of the individuals (120 out of 325) included in our analyses. It was thus not possible to fully account for the possible selective disappearance of individuals with poor immune performance or high pathogens prevalence, by including longevity as a covariate^70^. However, to control for such effect, we replicated our analyses on a subset of individuals (n = 120) by including as a fixed factor whether or not individuals reached 8 years of age. Models including this longevity metric were never retained (see Table S9 for a description of these models), suggesting that selective disappearance did not influence the outcome of our analyses.

All analyses were carried out in R version 3.2.3^77^ and using the function lmer from package lme4^78^.

### Data accessibility

All data will be deposited in Dryad.

## Electronic supplementary material

Supporting Information
