# Supplementary material for: Immunosenescence patterns differ between populations but not between sexes in a long-lived mammal
Source: Sci Rep. 2017 Oct 20;7:13700. doi: 10.1038/s41598-017-13686-5 (PMC5651810; doi:10.1038/s41598-017-13686-5)

Supporting information

Immunosenescence patterns differ between populations but not between sexes in a long-lived mammal.

AUTHORS: L. Cheynel, J-F. Lemaître, J-M. Gaillard, B. Rey, G. Bourgoin, H. Ferté, M. Jégo, F. Débias, M. Pellerin, L. Jacob and E. Gilot-Fromont.

Table S1. Composition of age classes in both sexes of the two populations of roe deer.

Table S2. Best models selected to describe senescence patterns of 12 immune and 4 parasitic traits, when including the two oldest males in Chizé (see Methods). We tested the effect of population ("pop"), sex ("sex") and 4 age functions (linear, factor, threshold and quadratic). Model comparison was based on AIC, "k" is the number of parameters, "weight" is the AIC weight of each model. All models included individual identity, the year of capture and the cohort of individuals as random effects. r2m and r2c are the marginal and conditional variance of the model, respectively.

Table S3. Pearson correlation matrix for the immune traits used in this study.

Table S4. Linear mixed effect model selected for WBC. The effect of 4 different age functions (factor, linear, threshold, quadratic), of sex (F: Female, M: Male) and of population (TF: Trois-Fontaines, CH: Chizé) were tested. All models included individual identity, the year of capture and the cohort of individuals as random effects. Statistical significance of age or body mass function are represented by * for p=0.05, ** for p=0.01 and *** for p=0.001. R2m and r2c are the marginal and conditional variance of the model, respectively. Values are presented ± Standard Error.

Table S5. Set of models fitted to assess senescence patterns of 12 immune parameters. We tested the effect of population ("P"), sex ("S), 4 age functions (linear "AL", factor "AF", threshold "AT" and quadratic "I(AL^2)") with all two and three-way interactions between them. "I" is the Intercept, "df" is the number of parameters, "delta" is the difference of AIC between the candidate model and the model having the lowest AIC, and "weight" the AIC weight of each model. We selected the model with the lowest AIC, and when some models had very similar explanatory power (delta<2), we selected the one which included fewer terms (shown in bold).

Table S6. Set of models fitted to assess senescence patterns of 4 parasitic traits. We tested the effect of population ("P"), sex ("S), 4 age functions (linear "AL", factor "AF", threshold "AT" and quadratic "I(AL^2)") with all two and three-way interactions between them. "I" is the Intercept, "df" is the number of parameters, "delta" is the difference of AIC between the candidate model and the model having the lowest AIC, and "weight" the AIC weight of each model. We selected the model with the lowest AIC, and when some models had very similar explanatory power (delta<2), we selected the one which included fewer terms (shown in bold).

Table S7. Linear mixed effect models selected for 12 immune parameters and 4 parasitic traits, analysed separately according to sex and roe deer populations (TF: Trois-Fontaines, CH: Chizé). The effect of different age functions (factor, linear, threshold, quadratic) was tested. All models included individual identity, the year of capture and the cohort of individuals as random effects; and were tested with and without body mass (BM) as a covariate. When the age threshold model was selected, "Age of change" represents the age at which the parameter begins to vary, and the "Parameter estimate" of the age function is the slope of the variation with age after the threshold age. Statistical significance of age or body mass function are represented by * for p=0.05, ** for p=0.01 and *** for p=0.001. R2m and R2c are the marginal and conditional variance of the model, respectively. Values are presented ± Standard Error.

Table S8. Linear mixed models describing the relationships between the 12 immune traits and the 4 parasitic traits of the study. Each immune trait was analysed as a function of parasite load, population and the interaction parasite load*population, considering 4 different parasite groups. Models included individual identity as a random effect. The "estimate" for the population or the parasitic trait is the slope of the variation. Statistical significance is represented by . for p=0.1, * for p=0.05, ** for p=0.01 and *** for p=0.001. Values are presented ± 1 SE.

Table S9. Set of models fitted to assess senescence patterns of immune traits including a fixed effect of the longevity of individuals, in males and females at Trois-Fontaines, and in females at Chizé. We do not have enough data on Chizé males to test these models. Model comparison was based on AIC, k is the number of parameters, ΔAIC is the difference of AIC between the candidate model and the model having the lowest AIC, and wi the AIC weight of each model. All models included individual identity, the year of capture and the cohort of individuals as random effects. The model with the lowest AIC is shown in italics; and models with very similar explanatory power (ΔAIC < 2), which included fewer terms, are shown in bold.

Fig. S1. Selection of the best threshold for the "threshold model" by maximum likelihood (see Methods). We used the deviance profiles of a continuous age model with a varying threshold and selected the age leading to the lowest deviance as the threshold age.

Table S1. Composition of age classes in both sexes of the two populations of roe deer.

	**TROIS-FONTAINES**		**CHIZE**	
Age (years)	Males	Females	Males	Females
2	29	38	29	24
3	18	29	25	20
4	17	24	16	19
5	22	14	11	23
6	20	23	9	16
7	12	9	7	14
8	8	12	8	27
9	8	7	4	11
10	3	10	2	13
11	3	5	1	5
12	2	4	0	5
13	1	4	0	1
14	0	0	1	0
15	0	1	0	0
16	0	1	0	0
**total**	**143**	**181**	**113**	**178**

Table S2. Best models selected to describe senescence patterns of 12 immune and 4 parasitic traits, when including the two oldest males in Chizé (see Methods). We tested the effect of population ("pop"), sex ("sex") and 4 age functions (linear, factor, threshold and quadratic). Model comparison was based on AIC, "k" is the number of parameters, "weight" is the AIC weight of each model. All models included individual identity, the year of capture and the cohort of individuals as random effects. r^2m and r^2c are the marginal and conditional variance of the model, respectively.

Trait	Best model selected	Age function	k	AIC	weight	r2m	r2c
Neutrophil count	age : pop : sex	Threshold (9 years)	12	2609.15	0.74	0.10	0.50
Monocyte count	constant	-	5	202.52	0.51	0.00	0.46
Basophil count	constant	-	5	-1017.03	0.41	0.00	0.28
Eosinophil count	sex	-	6	-690.54	0.12	0.02	0.15
Hemagglutination	age : pop	Threshold (8 years)	8	2043.18	0.43	0.01	0.44
Hemolysis	age + pop	Threshold (8 years)	7	1771.51	0.14	0.01	0.61
Alpha1-globulin	age + pop + sex	Threshold (8 years)	8	1093.19	0.09	0.03	0.50
Alpha2-globulin	constant	-	5	2278.83	0.07	0.00	0.26
Beta-globulin	age + pop + sex	Linear	8	2656.85	0.23	0.12	0.42
Haptoglobin	age : pop + sex	Threshold (9 years)	9	1839.66	0.31	0.08	0.15
Gamma-globulin	age + pop	Threshold (4 years)	7	3653.52	0.36	0.22	0.65
Lymphocyte count	Age + I(age^2) + pop	Quadratic	8	1509.26	0.14	0.14	0.37
Gastro-intestinal strongyles	age : sex	Threshold (9 years)	8	6105.00	0.32	0.10	0.75
Trichuris sp.	age : sex : pop	Factor	48	6991.74	0.99	0.34	0.39
Protostrongylids	age : sex : pop	Threshold (11 years)	12	2936.80	0.99	0.50	0.50
Coccidia	pop	-	6	10756.79	0.38	0.00	0.99

Table S3. Pearson correlation matrix for the immune traits used in this study.

	WBC	Neutrophil	Eosinophil	Basophil	Monocyte	Lymphocyte	Alpha-1-globulin	Alpha-2-globulin	Beta-globulin	Gamma-globulin	Haptoglobin	Hemag-glutination	Hemo-lysis
WBC	1.00	0.91	0.08	0.08	0.07	0.48	0.04	-0.01	-0.01	-0.09	0.04	-0.01	0.10
Neutrophil	0.91	1.00	-0.02	-0.03	-0.07	0.12	0.05	0.05	0.07	-0.05	0.08	-0.03	0.13
Eosinophil	0.08	-0.02	1.00	0.27	0.09	0.07	-0.07	0.03	0.03	0.07	-0.03	-0.05	-0.04
Basophil	0.08	-0.03	0.27	1.00	0.22	0.05	0.00	-0.05	0.04	0.11	0.10	0.04	-0.05
Monocyte	0.07	-0.07	0.09	0.22	1.00	-0.09	-0.13	-0.12	-0.06	0.01	0.05	0.01	-0.24
Lymphocyte	0.48	0.12	0.07	0.05	-0.09	1.00	0.04	-0.09	-0.15	-0.13	-0.07	0.03	0.07
Alpha-1-globulin	0.04	0.05	-0.07	0.00	-0.13	0.04	1.00	0.39	0.52	0.44	0.24	-0.04	-0.05
Alpha-2-globulin	-0.01	0.05	0.03	-0.05	-0.12	-0.09	0.39	1.00	0.17	0.26	-0.04	-0.06	0.03
Beta-globulin	-0.01	0.07	0.03	0.04	-0.06	-0.15	0.52	0.17	1.00	0.65	0.63	-0.07	-0.02
Gamma-globulin	-0.09	-0.05	0.07	0.11	0.01	-0.13	0.44	0.26	0.65	1.00	0.32	0.04	-0.08
Haptoglobin	0.04	0.08	-0.03	0.10	0.05	-0.07	0.24	-0.04	0.63	0.32	1.00	0.02	0.00
Hemagglutination	-0.01	-0.03	-0.05	0.04	0.01	0.03	-0.04	-0.06	-0.07	0.04	0.02	1.00	0.45
Hemolysis	0.10	0.13	-0.04	-0.05	-0.24	0.07	-0.05	0.03	-0.02	-0.08	0.00	0.45	1.00

Table S4. Linear mixed effect model selected for WBC. The effect of 4 different age functions (factor, linear, threshold, quadratic), of sex (F: Female, M: Male) and of population (TF: Trois-Fontaines, CH: Chizé) were tested. All models included individual identity, the year of capture and the cohort of individuals as random effects. Statistical significance of age or body mass function are represented by * for p=0.05, ** for p=0.01 and *** for p=0.001. R2m and r2c are the marginal and conditional variance of the model, respectively. Values are presented ± Standard Error.

Immune trait	Best model selected	Age function	Variable	Parameter estimate ± SE	t-value	p	r2m	r2c
**White Blood Cells count**	**Age +I(age^2)*sex + pop**	**quadratic**	Intercept	9.87 ±0.45	21.91	***	0.13	0.52
			Age	-0.36 ±0.15	-2.39	*		
			I(age^2)	0.04 ±0.01	3.17	**		
			Sex (M)	0.35 ±0.33	1.05	-		
			Pop (CH)	-1.74 ±0.33	-6.98	***		
			I(age^2) : sex (M)	-0.02 ±0.01	-2.76	**		

**Predicted value at two years**			
**♂ TF**	**♀TF**	**♂ CH**	**♀ CH**
8.93 ±0.28	8.90 ±0.39	7.50 ±0.41	7.49 ±0.23

Table S5. Set of models fitted to assess senescence patterns of 12 immune parameters. We tested the effect of population (“P”), sex (“S), 4 age functions (linear “AL”, factor “AF”, threshold “AT” and quadratic “I(AL^2)”) with all two and three-way interactions between them. “I” is the Intercept, “df” is the number of parameters, “delta” is the difference of AIC between the candidate model and the model having the lowest AIC, and “weight” the AIC weight of each model. We selected the model with the lowest AIC, and when some models had very similar explanatory power (delta<2), we selected the one which included fewer terms (shown in bold).

NEUTROPHIL COUNT

	I	AL	P	S	AL:P	AL:S	P:S	AL:P:S	NA	NA	NA	NA	df	AIC	delta	weight
24	5.71	0.15	+	+	NA	+	NA	NA	NA	NA	NA	NA	9	2588.20	0.00	0.50
56	5.75	0.15	+	+	NA	+	+	NA	NA	NA	NA	NA	10	2590.11	1.90	0.19
32	5.69	0.16	+	+	+	+	NA	NA	NA	NA	NA	NA	10	2590.25	2.05	0.18
64	5.73	0.16	+	+	+	+	+	NA	NA	NA	NA	NA	11	2592.17	3.97	0.07
128	5.62	0.18	+	+	+	+	+	+	NA	NA	NA	NA	12	2593.44	5.24	0.04
4	6.11	0.06	+	NA	NA	NA	NA	NA	NA	NA	NA	NA	7	2597.30	9.09	0.01
3	6.41	NA	+	NA	NA	NA	NA	NA	NA	NA	NA	NA	6	2597.92	9.72	0.00
8	6.18	0.06	+	+	NA	NA	NA	NA	NA	NA	NA	NA	8	2598.97	10.76	0.00
7	6.49	NA	+	+	NA	NA	NA	NA	NA	NA	NA	NA	7	2599.31	11.11	0.00
12	6.14	0.05	+	NA	+	NA	NA	NA	NA	NA	NA	NA	8	2599.31	11.11	0.00
40	6.23	0.06	+	+	NA	NA	+	NA	NA	NA	NA	NA	9	2600.67	12.46	0.00
16	6.20	0.05	+	+	+	NA	NA	NA	NA	NA	NA	NA	9	2601.01	12.81	0.00
39	6.53	NA	+	+	NA	NA	+	NA	NA	NA	NA	NA	8	2601.17	12.97	0.00
48	6.26	0.05	+	+	+	NA	+	NA	NA	NA	NA	NA	10	2602.69	14.49	0.00
22	5.21	0.14	NA	+	NA	+	NA	NA	NA	NA	NA	NA	8	2612.32	24.11	0.00
2	5.59	0.06	NA	NA	NA	NA	NA	NA	NA	NA	NA	NA	6	2617.69	29.49	0.00
1	5.90	NA	NA	NA	NA	NA	NA	NA	NA	NA	NA	NA	5	2618.46	30.25	0.00
6	5.66	0.06	NA	+	NA	NA	NA	NA	NA	NA	NA	NA	7	2619.44	31.24	0.00
5	5.97	NA	NA	+	NA	NA	NA	NA	NA	NA	NA	NA	6	2619.95	31.75	0.00
	**I**	**AF**	**P**	**S**	**AF:P**	**AF:S**	**P:S**	**AF:P:S**	**NA**	**NA**	**NA**	**NA**	**df**	**AIC**	**delta**	**weight**
3	6.41	NA	+	NA	NA	NA	NA	NA	NA	NA	NA	NA	6	2597.92	0.00	0.32
4	6.43	+	+	NA	NA	NA	NA	NA	NA	NA	NA	NA	16	2598.09	0.17	0.29
7	6.49	NA	+	+	NA	NA	NA	NA	NA	NA	NA	NA	7	2599.31	1.39	0.16
8	6.48	+	+	+	NA	NA	NA	NA	NA	NA	NA	NA	17	2599.98	2.05	0.11
39	6.53	NA	+	+	NA	NA	+	NA	NA	NA	NA	NA	8	2601.17	3.25	0.06
40	6.51	+	+	+	NA	NA	+	NA	NA	NA	NA	NA	18	2601.94	4.01	0.04
12	6.53	+	+	NA	+	NA	NA	NA	NA	NA	NA	NA	26	2606.04	8.12	0.01
24	6.34	+	+	+	NA	+	NA	NA	NA	NA	NA	NA	27	2607.86	9.94	0.00
16	6.59	+	+	+	+	NA	NA	NA	NA	NA	NA	NA	27	2607.95	10.03	0.00
48	6.63	+	+	+	+	NA	+	NA	NA	NA	NA	NA	28	2609.94	12.02	0.00
56	6.36	+	+	+	NA	+	+	NA	NA	NA	NA	NA	28	2610.00	12.08	0.00
32	6.43	+	+	+	+	+	NA	NA	NA	NA	NA	NA	37	2616.20	18.27	0.00
2	5.95	+	NA	NA	NA	NA	NA	NA	NA	NA	NA	NA	15	2616.48	18.55	0.00
64	6.45	+	+	+	+	+	+	NA	NA	NA	NA	NA	38	2618.40	20.48	0.00
6	6.00	+	NA	+	NA	NA	NA	NA	NA	NA	NA	NA	16	2618.42	20.50	0.00
1	5.90	NA	NA	NA	NA	NA	NA	NA	NA	NA	NA	NA	5	2618.46	20.53	0.00
5	5.97	NA	NA	+	NA	NA	NA	NA	NA	NA	NA	NA	6	2619.95	22.03	0.00
128	6.34	+	+	+	+	+	+	+	NA	NA	NA	NA	46	2627.36	29.44	0.00
22	5.88	+	NA	+	NA	+	NA	NA	NA	NA	NA	NA	26	2629.50	31.58	0.00
	**I**	**AT**	**P**	**S**	**AT:P**	**AT:S**	**P:S**	**AT:P:S**	**NA**	**NA**	**NA**	**NA**	**df**	**AIC**	**delta**	**weight**
4	2.02	0.48	+	NA	NA	NA	NA	NA	NA	NA	NA	NA	7	2590.51	0.00	0.25
24	0.67	0.63	+	+	NA	+	NA	NA	NA	NA	NA	NA	9	2590.65	0.14	0.23
12	2.69	0.40	+	NA	+	NA	NA	NA	NA	NA	NA	NA	8	2592.21	1.69	0.11
8	2.15	0.47	+	+	NA	NA	NA	NA	NA	NA	NA	NA	8	2592.32	1.81	0.10
56	0.72	0.63	+	+	NA	+	+	NA	NA	NA	NA	NA	10	2592.64	2.13	0.08
32	0.52	0.64	+	+	+	+	NA	NA	NA	NA	NA	NA	10	2592.71	2.20	0.08
16	2.80	0.40	+	+	+	NA	NA	NA	NA	NA	NA	NA	9	2594.04	3.53	0.04
40	2.18	0.47	+	+	NA	NA	+	NA	NA	NA	NA	NA	9	2594.16	3.64	0.04
64	0.63	0.64	+	+	+	+	+	NA	NA	NA	NA	NA	11	2594.72	4.20	0.03
48	2.90	0.39	+	+	+	NA	+	NA	NA	NA	NA	NA	10	2595.82	5.30	0.02
128	0.74	0.62	+	+	+	+	+	+	NA	NA	NA	NA	12	2596.70	6.18	0.01
3	6.41	NA	+	NA	NA	NA	NA	NA	NA	NA	NA	NA	6	2597.92	7.41	0.01
7	6.49	NA	+	+	NA	NA	NA	NA	NA	NA	NA	NA	7	2599.31	8.80	0.00
39	6.53	NA	+	+	NA	NA	+	NA	NA	NA	NA	NA	8	2601.17	10.66	0.00
2	1.06	0.53	NA	NA	NA	NA	NA	NA	NA	NA	NA	NA	6	2609.19	18.68	0.00
22	-0.16	0.66	NA	+	NA	+	NA	NA	NA	NA	NA	NA	8	2610.34	19.82	0.00
6	1.16	0.52	NA	+	NA	NA	NA	NA	NA	NA	NA	NA	7	2611.08	20.56	0.00
1	5.90	NA	NA	NA	NA	NA	NA	NA	NA	NA	NA	NA	5	2618.46	27.94	0.00
5	5.97	NA	NA	+	NA	NA	NA	NA	NA	NA	NA	NA	6	2619.95	29.44	0.00
	**I**	**AL**	**I(AL^2)**	**P**	**S**	**AL:P**	**AL:S**	**I(AL^2):P**	**I(AL^2):S**	**P:S**	**AL:P:S**	**I(AL^2):P:S**	**df**	**AIC**	**delta**	**weight**
144	6.61	-0.24	0.03	+	+	NA	NA	NA	+	NA	NA	NA	10	2583.83	0.00	0.15
**143**	**6.00**	**NA**	**0.01**	**+**	**+**	**NA**	**NA**	**NA**	**+**	**NA**	**NA**	**NA**	**9**	**2584.89**	**1.05**	**0.09**
48	6.44	-0.16	0.03	+	+	NA	+	NA	NA	NA	NA	NA	10	2585.09	1.25	0.08
176	6.69	-0.27	0.03	+	+	NA	+	NA	+	NA	NA	NA	11	2585.81	1.98	0.06
400	6.63	-0.24	0.03	+	+	NA	NA	NA	+	+	NA	NA	11	2585.85	2.02	0.06
208	6.59	-0.24	0.03	+	+	NA	NA	+	+	NA	NA	NA	11	2585.89	2.06	0.05
160	6.60	-0.24	0.03	+	+	+	NA	NA	+	NA	NA	NA	11	2585.90	2.07	0.05
399	6.04	NA	0.01	+	+	NA	NA	NA	+	+	NA	NA	10	2586.82	2.99	0.03
207	5.98	NA	0.01	+	+	NA	NA	+	+	NA	NA	NA	10	2586.87	3.04	0.03
304	6.46	-0.16	0.02	+	+	NA	+	NA	NA	+	NA	NA	11	2587.07	3.23	0.03
64	6.44	-0.16	0.03	+	+	+	+	NA	NA	NA	NA	NA	11	2587.16	3.33	0.03
112	6.44	-0.16	0.03	+	+	NA	+	+	NA	NA	NA	NA	11	2587.16	3.33	0.03
432	6.70	-0.27	0.03	+	+	NA	+	NA	+	+	NA	NA	12	2587.84	4.01	0.02
240	6.68	-0.28	0.03	+	+	NA	+	+	+	NA	NA	NA	12	2587.86	4.03	0.02
192	6.68	-0.27	0.03	+	+	+	+	NA	+	NA	NA	NA	12	2587.89	4.05	0.02
224	6.67	-0.27	0.03	+	+	+	NA	+	+	NA	NA	NA	12	2587.90	4.06	0.02
464	6.61	-0.24	0.03	+	+	NA	NA	+	+	+	NA	NA	12	2587.92	4.09	0.02
416	6.62	-0.24	0.03	+	+	+	NA	NA	+	+	NA	NA	12	2587.93	4.10	0.02
46	5.71	0.15	NA	+	+	NA	+	NA	NA	NA	NA	NA	9	2588.20	4.37	0.02
463	6.01	NA	0.01	+	+	NA	NA	+	+	+	NA	NA	11	2588.84	5.01	0.01
320	6.47	-0.16	0.03	+	+	+	+	NA	NA	+	NA	NA	12	2589.15	5.31	0.01
368	6.46	-0.16	0.02	+	+	NA	+	+	NA	+	NA	NA	12	2589.15	5.32	0.01
128	6.47	-0.18	0.03	+	+	+	+	+	NA	NA	NA	NA	12	2589.23	5.40	0.01
1488	6.62	-0.26	0.03	+	+	NA	NA	+	+	+	NA	+	13	2589.32	5.49	0.01
832	6.38	-0.16	0.03	+	+	+	+	NA	NA	+	+	NA	13	2589.79	5.96	0.01
256	6.78	-0.32	0.04	+	+	+	+	+	+	NA	NA	NA	13	2589.85	6.01	0.01
496	6.69	-0.27	0.03	+	+	NA	+	+	+	+	NA	NA	13	2589.91	6.08	0.01
448	6.69	-0.27	0.03	+	+	+	+	NA	+	+	NA	NA	13	2589.93	6.10	0.01
480	6.69	-0.27	0.03	+	+	+	NA	+	+	+	NA	NA	13	2589.94	6.10	0.01
302	5.75	0.15	NA	+	+	NA	+	NA	NA	+	NA	NA	10	2590.11	6.27	0.01
62	5.69	0.16	NA	+	+	+	+	NA	NA	NA	NA	NA	10	2590.25	6.42	0.01
8	6.98	-0.33	0.03	+	NA	NA	NA	NA	NA	NA	NA	NA	8	2590.51	6.67	0.01
1487	5.98	NA	0.02	+	+	NA	NA	+	+	+	NA	+	12	2590.67	6.83	0.01
960	6.58	-0.25	0.03	+	+	+	+	NA	+	+	+	NA	14	2590.96	7.13	0.00
384	6.50	-0.18	0.03	+	+	+	+	+	NA	+	NA	NA	13	2591.22	7.39	0.00
1520	6.65	-0.28	0.04	+	+	NA	+	+	+	+	NA	+	14	2591.39	7.56	0.00
1504	6.63	-0.27	0.04	+	+	+	NA	+	+	+	NA	+	14	2591.42	7.58	0.00
896	6.33	-0.13	0.02	+	+	+	+	+	NA	+	+	NA	14	2591.85	8.02	0.00
512	6.79	-0.32	0.04	+	+	+	+	+	+	+	NA	NA	14	2591.91	8.07	0.00
318	5.73	0.16	NA	+	+	+	+	NA	NA	+	NA	NA	11	2592.17	8.34	0.00
16	7.03	-0.33	0.03	+	+	NA	NA	NA	NA	NA	NA	NA	9	2592.31	8.48	0.00
72	7.02	-0.33	0.03	+	NA	NA	NA	+	NA	NA	NA	NA	9	2592.41	8.57	0.00
24	7.04	-0.35	0.03	+	NA	+	NA	NA	NA	NA	NA	NA	9	2592.43	8.60	0.00
1024	6.60	-0.26	0.03	+	+	+	+	+	+	+	+	NA	15	2593.06	9.23	0.00
830	5.62	0.18	NA	+	+	+	+	NA	NA	+	+	NA	12	2593.44	9.61	0.00
1536	6.69	-0.29	0.04	+	+	+	+	+	+	+	NA	+	15	2593.49	9.66	0.00
272	7.06	-0.33	0.03	+	+	NA	NA	NA	NA	+	NA	NA	10	2594.19	10.36	0.00
80	7.06	-0.33	0.03	+	+	NA	NA	+	NA	NA	NA	NA	10	2594.26	10.42	0.00
32	7.08	-0.34	0.03	+	+	+	NA	NA	NA	NA	NA	NA	10	2594.28	10.45	0.00
7	6.17	NA	0.01	+	NA	NA	NA	NA	NA	NA	NA	NA	7	2594.43	10.60	0.00
2048	6.44	-0.18	0.03	+	+	+	+	+	+	+	+	+	16	2594.43	10.60	0.00
88	6.99	-0.32	0.03	+	NA	+	NA	+	NA	NA	NA	NA	10	2594.47	10.64	0.00
336	7.10	-0.33	0.03	+	+	NA	NA	+	NA	+	NA	NA	11	2596.09	12.26	0.00
288	7.12	-0.34	0.03	+	+	+	NA	NA	NA	+	NA	NA	11	2596.13	12.29	0.00
15	6.23	NA	0.01	+	+	NA	NA	NA	NA	NA	NA	NA	8	2596.21	12.38	0.00
96	7.03	-0.32	0.03	+	+	+	NA	+	NA	NA	NA	NA	11	2596.32	12.49	0.00
71	6.20	NA	0.01	+	NA	NA	NA	+	NA	NA	NA	NA	8	2596.41	12.58	0.00
6	6.11	0.06	NA	+	NA	NA	NA	NA	NA	NA	NA	NA	7	2597.30	13.46	0.00
271	6.29	NA	0.01	+	+	NA	NA	NA	NA	+	NA	NA	9	2597.88	14.04	0.00
5	6.41	NA	NA	+	NA	NA	NA	NA	NA	NA	NA	NA	6	2597.92	14.09	0.00
352	7.06	-0.31	0.03	+	+	+	NA	+	NA	+	NA	NA	12	2598.16	14.33	0.00
79	6.25	NA	0.01	+	+	NA	NA	+	NA	NA	NA	NA	9	2598.22	14.39	0.00
14	6.18	0.06	NA	+	+	NA	NA	NA	NA	NA	NA	NA	8	2598.97	15.13	0.00
13	6.49	NA	NA	+	+	NA	NA	NA	NA	NA	NA	NA	7	2599.31	15.48	0.00
22	6.14	0.05	NA	+	NA	+	NA	NA	NA	NA	NA	NA	8	2599.31	15.48	0.00
335	6.31	NA	0.01	+	+	NA	NA	+	NA	+	NA	NA	10	2599.85	16.01	0.00
270	6.23	0.06	NA	+	+	NA	NA	NA	NA	+	NA	NA	9	2600.67	16.83	0.00
30	6.20	0.05	NA	+	+	+	NA	NA	NA	NA	NA	NA	9	2601.01	17.18	0.00
269	6.53	NA	NA	+	+	NA	NA	NA	NA	+	NA	NA	8	2601.17	17.34	0.00
286	6.26	0.05	NA	+	+	+	NA	NA	NA	+	NA	NA	10	2602.69	18.86	0.00
140	6.21	-0.29	0.03	NA	+	NA	NA	NA	+	NA	NA	NA	9	2605.95	22.12	0.00
44	6.08	-0.23	0.03	NA	+	NA	+	NA	NA	NA	NA	NA	9	2607.35	23.51	0.00
172	6.38	-0.36	0.04	NA	+	NA	+	NA	+	NA	NA	NA	10	2607.62	23.79	0.00
139	5.47	NA	0.01	NA	+	NA	NA	NA	+	NA	NA	NA	8	2608.23	24.40	0.00
4	6.55	-0.36	0.04	NA	NA	NA	NA	NA	NA	NA	NA	NA	7	2609.45	25.62	0.00
12	6.60	-0.36	0.04	NA	+	NA	NA	NA	NA	NA	NA	NA	8	2611.33	27.49	0.00
42	5.21	0.14	NA	NA	+	NA	+	NA	NA	NA	NA	NA	8	2612.32	28.48	0.00
3	5.66	NA	0.01	NA	NA	NA	NA	NA	NA	NA	NA	NA	6	2614.43	30.59	0.00
11	5.71	NA	0.01	NA	+	NA	NA	NA	NA	NA	NA	NA	7	2616.28	32.45	0.00
2	5.59	0.06	NA	NA	NA	NA	NA	NA	NA	NA	NA	NA	6	2617.69	33.86	0.00
1	5.90	NA	NA	NA	NA	NA	NA	NA	NA	NA	NA	NA	5	2618.46	34.62	0.00
10	5.66	0.06	NA	NA	+	NA	NA	NA	NA	NA	NA	NA	7	2619.44	35.61	0.00

9	5.97	NA	NA	NA	+	NA	NA	NA	NA	NA	NA	NA	6	2619.95	36.12	0.00

MONOCYTE COUNT

	I	AL	P	S	AL:P	AL:S	P:S	AL:P:S	NA	NA	NA	NA	df	AIC	delta	weight
1	0.29	NA	NA	NA	NA	NA	NA	NA	NA	NA	NA	NA	5	203.82	0.00	0.28
2	0.31	0.00	NA	NA	NA	NA	NA	NA	NA	NA	NA	NA	6	204.87	1.05	0.17
3	0.30	NA	+	NA	NA	NA	NA	NA	NA	NA	NA	NA	6	205.70	1.88	0.11
5	0.29	NA	NA	+	NA	NA	NA	NA	NA	NA	NA	NA	6	205.79	1.97	0.11
6	0.32	0.00	NA	+	NA	NA	NA	NA	NA	NA	NA	NA	7	206.74	2.91	0.07
4	0.32	0.00	+	NA	NA	NA	NA	NA	NA	NA	NA	NA	7	206.76	2.94	0.07
7	0.30	NA	+	+	NA	NA	NA	NA	NA	NA	NA	NA	7	207.67	3.85	0.04
22	0.33	-0.01	NA	+	NA	+	NA	NA	NA	NA	NA	NA	8	208.24	4.42	0.03
12	0.31	0.00	+	NA	+	NA	NA	NA	NA	NA	NA	NA	8	208.59	4.77	0.03
8	0.32	0.00	+	+	NA	NA	NA	NA	NA	NA	NA	NA	8	208.61	4.79	0.03
39	0.29	NA	+	+	NA	NA	+	NA	NA	NA	NA	NA	8	209.34	5.51	0.02
40	0.32	0.00	+	+	NA	NA	+	NA	NA	NA	NA	NA	9	210.11	6.29	0.01
24	0.34	-0.01	+	+	NA	+	NA	NA	NA	NA	NA	NA	9	210.19	6.36	0.01
16	0.31	0.00	+	+	+	NA	NA	NA	NA	NA	NA	NA	9	210.40	6.58	0.01
48	0.31	0.00	+	+	+	NA	+	NA	NA	NA	NA	NA	10	211.74	7.92	0.01
56	0.33	-0.01	+	+	NA	+	+	NA	NA	NA	NA	NA	10	211.76	7.94	0.01
32	0.33	0.00	+	+	+	+	NA	NA	NA	NA	NA	NA	10	212.02	8.20	0.00
64	0.32	0.00	+	+	+	+	+	NA	NA	NA	NA	NA	11	213.46	9.64	0.00
128	0.31	0.00	+	+	+	+	+	+	NA	NA	NA	NA	12	215.38	11.56	0.00
	I	AF	P	S	AF:P	AF:S	P:S	AF:P:S	NA	NA	NA	NA	df	AIC	delta	weight
1	0.29	NA	NA	NA	NA	NA	NA	NA	NA	NA	NA	NA	5	203.82	0.00	0.51
3	0.30	NA	+	NA	NA	NA	NA	NA	NA	NA	NA	NA	6	205.70	1.88	0.20
5	0.29	NA	NA	+	NA	NA	NA	NA	NA	NA	NA	NA	6	205.79	1.97	0.19
7	0.30	NA	+	+	NA	NA	NA	NA	NA	NA	NA	NA	7	207.67	3.85	0.07
39	0.29	NA	+	+	NA	NA	+	NA	NA	NA	NA	NA	8	209.34	5.51	0.03
2	0.33	+	NA	NA	NA	NA	NA	NA	NA	NA	NA	NA	15	218.18	14.36	0.00
4	0.34	+	+	NA	NA	NA	NA	NA	NA	NA	NA	NA	16	220.11	16.29	0.00
6	0.34	+	NA	+	NA	NA	NA	NA	NA	NA	NA	NA	16	220.19	16.36	0.00
8	0.34	+	+	+	NA	NA	NA	NA	NA	NA	NA	NA	17	222.10	18.28	0.00
40	0.33	+	+	+	NA	NA	+	NA	NA	NA	NA	NA	18	223.51	19.69	0.00
22	0.38	+	NA	+	NA	+	NA	NA	NA	NA	NA	NA	26	233.03	29.21	0.00
12	0.34	+	+	NA	+	NA	NA	NA	NA	NA	NA	NA	26	233.53	29.71	0.00
24	0.38	+	+	+	NA	+	NA	NA	NA	NA	NA	NA	27	235.18	31.36	0.00
16	0.34	+	+	+	+	NA	NA	NA	NA	NA	NA	NA	27	235.59	31.77	0.00
56	0.37	+	+	+	NA	+	+	NA	NA	NA	NA	NA	28	236.53	32.71	0.00
48	0.33	+	+	+	+	NA	+	NA	NA	NA	NA	NA	28	237.04	33.22	0.00
32	0.38	+	+	+	+	+	NA	NA	NA	NA	NA	NA	37	246.79	42.97	0.00
64	0.37	+	+	+	+	+	+	NA	NA	NA	NA	NA	38	248.04	44.22	0.00
128	0.38	+	+	+	+	+	+	+	NA	NA	NA	NA	46	259.75	55.93	0.00
	I	AT	P	S	AT:P	AT:S	P:S	AT:P:S	NA	NA	NA	NA	df	AIC	delta	weight
1	0.29	NA	NA	NA	NA	NA	NA	NA	NA	NA	NA	NA	5	203.82	0.00	0.32
2	0.31	0.00	NA	NA	NA	NA	NA	NA	NA	NA	NA	NA	6	205.38	1.56	0.15
3	0.30	NA	+	NA	NA	NA	NA	NA	NA	NA	NA	NA	6	205.70	1.88	0.12
5	0.29	NA	NA	+	NA	NA	NA	NA	NA	NA	NA	NA	6	205.79	1.97	0.12
4	0.32	0.00	+	NA	NA	NA	NA	NA	NA	NA	NA	NA	7	207.27	3.45	0.06
6	0.32	0.00	NA	+	NA	NA	NA	NA	NA	NA	NA	NA	7	207.28	3.46	0.06
7	0.30	NA	+	+	NA	NA	NA	NA	NA	NA	NA	NA	7	207.67	3.85	0.05
12	0.30	0.00	+	NA	+	NA	NA	NA	NA	NA	NA	NA	8	208.96	5.14	0.02
8	0.32	0.00	+	+	NA	NA	NA	NA	NA	NA	NA	NA	8	209.16	5.34	0.02
22	0.32	-0.01	NA	+	NA	+	NA	NA	NA	NA	NA	NA	8	209.24	5.42	0.02
39	0.29	NA	+	+	NA	NA	+	NA	NA	NA	NA	NA	8	209.34	5.51	0.02
40	0.32	0.00	+	+	NA	NA	+	NA	NA	NA	NA	NA	9	210.73	6.91	0.01
16	0.31	0.00	+	+	+	NA	NA	NA	NA	NA	NA	NA	9	210.81	6.99	0.01
24	0.33	0.00	+	+	NA	+	NA	NA	NA	NA	NA	NA	9	211.15	7.33	0.01
48	0.30	0.00	+	+	+	NA	+	NA	NA	NA	NA	NA	10	212.20	8.38	0.00
56	0.32	-0.01	+	+	NA	+	+	NA	NA	NA	NA	NA	10	212.75	8.93	0.00
32	0.31	0.00	+	+	+	+	NA	NA	NA	NA	NA	NA	10	212.83	9.01	0.00
64	0.30	0.00	+	+	+	+	+	NA	NA	NA	NA	NA	11	214.26	10.43	0.00
128	0.29	0.00	+	+	+	+	+	+	NA	NA	NA	NA	12	216.10	12.28	0.00
	I	AL	I(AL^2)	P	S	AL:P	AL:S	I(AL^2):P	I(AL^2):S	P:S	AL:P:S	I(AL^2):P:S	df	AIC	delta	weight
1	0.29	NA	NA	NA	NA	NA	NA	NA	NA	NA	NA	NA	5	203.82	0.00	0.16
2	0.31	0.00	NA	NA	NA	NA	NA	NA	NA	NA	NA	NA	6	204.87	1.05	0.09
3	0.30	NA	0.00	NA	NA	NA	NA	NA	NA	NA	NA	NA	6	205.33	1.51	0.07
5	0.30	NA	NA	+	NA	NA	NA	NA	NA	NA	NA	NA	6	205.70	1.88	0.06
4	0.36	-0.02	0.00	NA	NA	NA	NA	NA	NA	NA	NA	NA	7	205.76	1.94	0.06
9	0.29	NA	NA	NA	+	NA	NA	NA	NA	NA	NA	NA	6	205.79	1.97	0.06
10	0.32	0.00	NA	NA	+	NA	NA	NA	NA	NA	NA	NA	7	206.74	2.91	0.04
6	0.32	0.00	NA	+	NA	NA	NA	NA	NA	NA	NA	NA	7	206.76	2.94	0.04
172	0.45	-0.06	0.00	NA	+	NA	+	NA	+	NA	NA	NA	10	207.07	3.25	0.03
7	0.30	NA	0.00	+	NA	NA	NA	NA	NA	NA	NA	NA	7	207.22	3.40	0.03
11	0.31	NA	0.00	NA	+	NA	NA	NA	NA	NA	NA	NA	7	207.23	3.41	0.03
13	0.30	NA	NA	+	+	NA	NA	NA	NA	NA	NA	NA	7	207.67	3.85	0.02
12	0.36	-0.02	0.00	NA	+	NA	NA	NA	NA	NA	NA	NA	8	207.67	3.85	0.02
8	0.36	-0.02	0.00	+	NA	NA	NA	NA	NA	NA	NA	NA	8	207.69	3.87	0.02
42	0.33	-0.01	NA	NA	+	NA	+	NA	NA	NA	NA	NA	8	208.24	4.42	0.02
22	0.31	0.00	NA	+	NA	+	NA	NA	NA	NA	NA	NA	8	208.59	4.77	0.01
14	0.32	0.00	NA	+	+	NA	NA	NA	NA	NA	NA	NA	8	208.61	4.79	0.01
44	0.39	-0.03	0.00	NA	+	NA	+	NA	NA	NA	NA	NA	9	208.75	4.93	0.01
71	0.30	NA	0.00	+	NA	NA	NA	+	NA	NA	NA	NA	8	208.82	5.00	0.01
15	0.31	NA	0.00	+	+	NA	NA	NA	NA	NA	NA	NA	8	209.11	5.29	0.01
176	0.45	-0.05	0.00	+	+	NA	+	NA	+	NA	NA	NA	11	209.13	5.31	0.01
139	0.31	NA	0.00	NA	+	NA	NA	NA	+	NA	NA	NA	8	209.23	5.41	0.01
269	0.29	NA	NA	+	+	NA	NA	NA	NA	+	NA	NA	8	209.34	5.51	0.01
140	0.37	-0.03	0.00	NA	+	NA	NA	NA	+	NA	NA	NA	9	209.48	5.66	0.01
72	0.35	-0.02	0.00	+	NA	NA	NA	+	NA	NA	NA	NA	9	209.48	5.66	0.01
16	0.37	-0.02	0.00	+	+	NA	NA	NA	NA	NA	NA	NA	9	209.60	5.78	0.01
24	0.35	-0.02	0.00	+	NA	+	NA	NA	NA	NA	NA	NA	9	209.63	5.81	0.01
270	0.32	0.00	NA	+	+	NA	NA	NA	NA	+	NA	NA	9	210.11	6.29	0.01
46	0.34	-0.01	NA	+	+	NA	+	NA	NA	NA	NA	NA	9	210.19	6.36	0.01
432	0.45	-0.06	0.00	+	+	NA	+	NA	+	+	NA	NA	12	210.39	6.56	0.01
30	0.31	0.00	NA	+	+	+	NA	NA	NA	NA	NA	NA	9	210.40	6.58	0.01
79	0.30	NA	0.00	+	+	NA	NA	+	NA	NA	NA	NA	9	210.65	6.83	0.01
271	0.30	NA	0.00	+	+	NA	NA	NA	NA	+	NA	NA	9	210.68	6.86	0.01
240	0.44	-0.05	0.00	+	+	NA	+	+	+	NA	NA	NA	12	210.75	6.92	0.00
48	0.39	-0.03	0.00	+	+	NA	+	NA	NA	NA	NA	NA	10	210.75	6.93	0.00
272	0.36	-0.02	0.00	+	+	NA	NA	NA	NA	+	NA	NA	10	210.97	7.14	0.00
192	0.44	-0.05	0.00	+	+	+	+	NA	+	NA	NA	NA	12	210.98	7.16	0.00
88	0.38	-0.03	0.00	+	NA	+	NA	+	NA	NA	NA	NA	10	211.08	7.26	0.00
143	0.31	NA	0.00	+	+	NA	NA	NA	+	NA	NA	NA	9	211.13	7.31	0.00
80	0.36	-0.02	0.00	+	+	NA	NA	+	NA	NA	NA	NA	10	211.34	7.52	0.00
144	0.38	-0.02	0.00	+	+	NA	NA	NA	+	NA	NA	NA	10	211.45	7.63	0.00
32	0.36	-0.02	0.00	+	+	+	NA	NA	NA	NA	NA	NA	10	211.50	7.68	0.00
496	0.43	-0.06	0.00	+	+	NA	+	+	+	+	NA	NA	13	211.73	7.91	0.00
286	0.31	0.00	NA	+	+	+	NA	NA	NA	+	NA	NA	10	211.74	7.92	0.00
302	0.33	-0.01	NA	+	+	NA	+	NA	NA	+	NA	NA	10	211.76	7.94	0.00
335	0.30	NA	0.00	+	+	NA	NA	+	NA	+	NA	NA	10	212.01	8.19	0.00
62	0.33	0.00	NA	+	+	+	+	NA	NA	NA	NA	NA	10	212.02	8.20	0.00
448	0.43	-0.05	0.00	+	+	+	+	NA	+	+	NA	NA	13	212.05	8.23	0.00
256	0.47	-0.07	0.00	+	+	+	+	+	+	NA	NA	NA	13	212.19	8.37	0.00
304	0.39	-0.03	0.00	+	+	NA	+	NA	NA	+	NA	NA	11	212.21	8.39	0.00
336	0.35	-0.02	0.00	+	+	NA	NA	+	NA	+	NA	NA	11	212.53	8.70	0.00
112	0.38	-0.03	0.00	+	+	NA	+	+	NA	NA	NA	NA	11	212.61	8.79	0.00
207	0.30	NA	0.00	+	+	NA	NA	+	+	NA	NA	NA	10	212.70	8.88	0.00
64	0.38	-0.03	0.00	+	+	+	+	NA	NA	NA	NA	NA	11	212.72	8.90	0.00
399	0.31	NA	0.00	+	+	NA	NA	NA	+	+	NA	NA	10	212.73	8.91	0.00
288	0.35	-0.02	0.00	+	+	+	NA	NA	NA	+	NA	NA	11	212.74	8.92	0.00
400	0.37	-0.03	0.00	+	+	NA	NA	NA	+	+	NA	NA	11	212.87	9.05	0.00
96	0.38	-0.03	0.00	+	+	+	NA	+	NA	NA	NA	NA	11	212.99	9.17	0.00
512	0.47	-0.07	0.01	+	+	+	+	+	+	+	NA	NA	14	213.13	9.31	0.00
208	0.37	-0.02	0.00	+	+	NA	NA	+	+	NA	NA	NA	11	213.26	9.44	0.00
160	0.37	-0.02	0.00	+	+	+	NA	NA	+	NA	NA	NA	11	213.40	9.57	0.00
318	0.32	0.00	NA	+	+	+	+	NA	NA	+	NA	NA	11	213.46	9.64	0.00
1520	0.43	-0.06	0.00	+	+	NA	+	+	+	+	NA	+	14	213.81	9.99	0.00
368	0.38	-0.03	0.00	+	+	NA	+	+	NA	+	NA	NA	12	213.92	10.10	0.00
463	0.30	NA	0.00	+	+	NA	NA	+	+	+	NA	NA	11	214.08	10.26	0.00
320	0.38	-0.03	0.00	+	+	+	+	NA	NA	+	NA	NA	12	214.09	10.27	0.00
960	0.43	-0.05	0.00	+	+	+	+	NA	+	+	+	NA	14	214.13	10.31	0.00
352	0.38	-0.03	0.00	+	+	+	NA	+	NA	+	NA	NA	12	214.16	10.34	0.00
128	0.41	-0.04	0.00	+	+	+	+	+	NA	NA	NA	NA	12	214.33	10.51	0.00
464	0.36	-0.02	0.00	+	+	NA	NA	+	+	+	NA	NA	12	214.51	10.69	0.00
416	0.36	-0.02	0.00	+	+	+	NA	NA	+	+	NA	NA	12	214.71	10.89	0.00
224	0.39	-0.03	0.00	+	+	+	NA	+	+	NA	NA	NA	12	214.95	11.13	0.00
1024	0.47	-0.07	0.01	+	+	+	+	+	+	+	+	NA	15	215.20	11.38	0.00
1536	0.47	-0.07	0.01	+	+	+	+	+	+	+	NA	+	15	215.22	11.39	0.00
830	0.31	0.00	NA	+	+	+	+	NA	NA	+	+	NA	12	215.38	11.56	0.00
384	0.40	-0.04	0.00	+	+	+	+	+	NA	+	NA	NA	13	215.62	11.80	0.00
832	0.37	-0.03	0.00	+	+	+	+	NA	NA	+	+	NA	13	215.90	12.08	0.00
1487	0.29	NA	0.00	+	+	NA	NA	+	+	+	NA	+	12	215.98	12.16	0.00
480	0.38	-0.03	0.00	+	+	+	NA	+	+	+	NA	NA	13	216.18	12.36	0.00
1488	0.36	-0.03	0.00	+	+	NA	NA	+	+	+	NA	+	13	216.21	12.39	0.00
2048	0.48	-0.07	0.01	+	+	+	+	+	+	+	+	+	16	217.26	13.44	0.00
896	0.39	-0.04	0.00	+	+	+	+	+	NA	+	+	NA	14	217.60	13.78	0.00
1504	0.38	-0.03	0.00	+	+	+	NA	+	+	+	NA	+	14	218.06	14.24	0.00

BASOPHIL COUNT

	I	AL	P	S	AL:P	AL:S	P:S	AL:P:S	NA	NA	NA	NA	df	AIC	delta	weight
1	0.07	NA	NA	NA	NA	NA	NA	NA	NA	NA	NA	NA	5	-1011.98	0.00	0.22
5	0.08	NA	NA	+	NA	NA	NA	NA	NA	NA	NA	NA	6	-1010.87	1.10	0.13
2	0.07	0.00	NA	NA	NA	NA	NA	NA	NA	NA	NA	NA	6	-1010.63	1.35	0.11
3	0.07	NA	+	NA	NA	NA	NA	NA	NA	NA	NA	NA	6	-1010.52	1.46	0.11
12	0.08	0.00	+	NA	+	NA	NA	NA	NA	NA	NA	NA	8	-1010.02	1.96	0.08
7	0.08	NA	+	+	NA	NA	NA	NA	NA	NA	NA	NA	7	-1009.35	2.63	0.06
6	0.07	0.00	NA	+	NA	NA	NA	NA	NA	NA	NA	NA	7	-1009.32	2.66	0.06
4	0.06	0.00	+	NA	NA	NA	NA	NA	NA	NA	NA	NA	7	-1009.17	2.80	0.05
16	0.08	0.00	+	+	+	NA	NA	NA	NA	NA	NA	NA	9	-1008.36	3.61	0.04
8	0.07	0.00	+	+	NA	NA	NA	NA	NA	NA	NA	NA	8	-1007.80	4.17	0.03
39	0.07	NA	+	+	NA	NA	+	NA	NA	NA	NA	NA	8	-1007.71	4.26	0.03
22	0.07	0.00	NA	+	NA	+	NA	NA	NA	NA	NA	NA	8	-1007.67	4.30	0.03
32	0.08	0.00	+	+	+	+	NA	NA	NA	NA	NA	NA	10	-1006.51	5.47	0.01
48	0.08	0.00	+	+	+	NA	+	NA	NA	NA	NA	NA	10	-1006.40	5.58	0.01
40	0.07	0.00	+	+	NA	NA	+	NA	NA	NA	NA	NA	9	-1006.07	5.91	0.01
24	0.07	0.00	+	+	NA	+	NA	NA	NA	NA	NA	NA	9	-1006.06	5.92	0.01
64	0.08	0.00	+	+	+	+	+	NA	NA	NA	NA	NA	11	-1004.56	7.41	0.01
56	0.06	0.00	+	+	NA	+	+	NA	NA	NA	NA	NA	10	-1004.36	7.62	0.00
128	0.08	0.00	+	+	+	+	+	+	NA	NA	NA	NA	12	-1004.07	7.91	0.00
	I	AF	P	S	AF:P	AF:S	P:S	AF:P:S	NA	NA	NA	NA	df	AIC	delta	weight
1	0.07	NA	NA	NA	NA	NA	NA	NA	NA	NA	NA	NA	5	-1011.98	0.00	0.41
5	0.08	NA	NA	+	NA	NA	NA	NA	NA	NA	NA	NA	6	-1010.87	1.10	0.24
3	0.07	NA	+	NA	NA	NA	NA	NA	NA	NA	NA	NA	6	-1010.52	1.46	0.20
7	0.08	NA	+	+	NA	NA	NA	NA	NA	NA	NA	NA	7	-1009.35	2.63	0.11
39	0.07	NA	+	+	NA	NA	+	NA	NA	NA	NA	NA	8	-1007.71	4.26	0.05
2	0.07	+	NA	NA	NA	NA	NA	NA	NA	NA	NA	NA	15	-997.47	14.50	0.00
4	0.06	+	+	NA	NA	NA	NA	NA	NA	NA	NA	NA	16	-996.04	15.94	0.00
6	0.07	+	NA	+	NA	NA	NA	NA	NA	NA	NA	NA	16	-996.02	15.96	0.00
8	0.07	+	+	+	NA	NA	NA	NA	NA	NA	NA	NA	17	-994.51	17.46	0.00
40	0.06	+	+	+	NA	NA	+	NA	NA	NA	NA	NA	18	-992.87	19.11	0.00
12	0.07	+	+	NA	+	NA	NA	NA	NA	NA	NA	NA	26	-989.23	22.75	0.00
16	0.07	+	+	+	+	NA	NA	NA	NA	NA	NA	NA	27	-987.39	24.59	0.00
48	0.07	+	+	+	+	NA	+	NA	NA	NA	NA	NA	28	-985.33	26.64	0.00
22	0.08	+	NA	+	NA	+	NA	NA	NA	NA	NA	NA	26	-984.57	27.40	0.00
24	0.08	+	+	+	NA	+	NA	NA	NA	NA	NA	NA	27	-983.09	28.88	0.00
56	0.07	+	+	+	NA	+	+	NA	NA	NA	NA	NA	28	-981.50	30.48	0.00
32	0.08	+	+	+	+	+	NA	NA	NA	NA	NA	NA	37	-972.69	39.29	0.00
64	0.08	+	+	+	+	+	+	NA	NA	NA	NA	NA	38	-970.60	41.37	0.00
128	0.08	+	+	+	+	+	+	+	NA	NA	NA	NA	46	-961.08	50.90	0.00
	I	AT	P	S	AT:P	AT:S	P:S	AT:P:S	NA	NA	NA	NA	df	AIC	delta	weight
1	0.07	NA	NA	NA	NA	NA	NA	NA	NA	NA	NA	NA	5	-1011.98	0.00	0.19
2	0.03	0.01	NA	NA	NA	NA	NA	NA	NA	NA	NA	NA	6	-1011.20	0.77	0.13
5	0.08	NA	NA	+	NA	NA	NA	NA	NA	NA	NA	NA	6	-1010.87	1.10	0.11
3	0.07	NA	+	NA	NA	NA	NA	NA	NA	NA	NA	NA	6	-1010.52	1.46	0.09
6	0.04	0.01	NA	+	NA	NA	NA	NA	NA	NA	NA	NA	7	-1009.85	2.13	0.07
4	0.02	0.01	+	NA	NA	NA	NA	NA	NA	NA	NA	NA	7	-1009.84	2.13	0.07
12	0.07	0.00	+	NA	+	NA	NA	NA	NA	NA	NA	NA	8	-1009.70	2.28	0.06
7	0.08	NA	+	+	NA	NA	NA	NA	NA	NA	NA	NA	7	-1009.35	2.63	0.05
22	0.01	0.01	NA	+	NA	+	NA	NA	NA	NA	NA	NA	8	-1009.23	2.75	0.05
8	0.03	0.01	+	+	NA	NA	NA	NA	NA	NA	NA	NA	8	-1008.41	3.57	0.03
16	0.07	0.00	+	+	+	NA	NA	NA	NA	NA	NA	NA	9	-1008.13	3.84	0.03
39	0.07	NA	+	+	NA	NA	+	NA	NA	NA	NA	NA	8	-1007.71	4.26	0.02
24	0.00	0.01	+	+	NA	+	NA	NA	NA	NA	NA	NA	9	-1007.71	4.27	0.02
32	0.05	0.00	+	+	+	+	NA	NA	NA	NA	NA	NA	10	-1006.82	5.15	0.01
40	0.03	0.01	+	+	NA	NA	+	NA	NA	NA	NA	NA	9	-1006.73	5.25	0.01
48	0.07	0.00	+	+	+	NA	+	NA	NA	NA	NA	NA	10	-1006.26	5.72	0.01
56	0.00	0.01	+	+	NA	+	+	NA	NA	NA	NA	NA	10	-1006.21	5.77	0.01
64	0.04	0.00	+	+	+	+	+	NA	NA	NA	NA	NA	11	-1005.08	6.90	0.01
128	0.05	0.00	+	+	+	+	+	+	NA	NA	NA	NA	12	-1003.64	8.33	0.00
	I	AL	I(AL^2)	P	S	AL:P	AL:S	I(AL^2):P	I(AL^2):S	P:S	AL:P:S	I(AL^2):P:S	df	AIC	delta	weight
1	0.07	NA	NA	NA	NA	NA	NA	NA	NA	NA	NA	NA	5	-1011.98	0.00	0.11
9	0.08	NA	NA	NA	+	NA	NA	NA	NA	NA	NA	NA	6	-1010.87	1.10	0.06
3	0.07	NA	0.00	NA	NA	NA	NA	NA	NA	NA	NA	NA	6	-1010.86	1.12	0.06
2	0.07	0.00	NA	NA	NA	NA	NA	NA	NA	NA	NA	NA	6	-1010.63	1.35	0.05
5	0.07	NA	NA	+	NA	NA	NA	NA	NA	NA	NA	NA	6	-1010.52	1.46	0.05
71	0.07	NA	0.00	+	NA	NA	NA	+	NA	NA	NA	NA	8	-1010.34	1.63	0.05
22	0.08	0.00	NA	+	NA	+	NA	NA	NA	NA	NA	NA	8	-1010.02	1.96	0.04
11	0.07	NA	0.00	NA	+	NA	NA	NA	NA	NA	NA	NA	7	-1009.51	2.47	0.03
7	0.07	NA	0.00	+	NA	NA	NA	NA	NA	NA	NA	NA	7	-1009.42	2.56	0.03
13	0.08	NA	NA	+	+	NA	NA	NA	NA	NA	NA	NA	7	-1009.35	2.63	0.03
10	0.07	0.00	NA	NA	+	NA	NA	NA	NA	NA	NA	NA	7	-1009.32	2.66	0.03
6	0.06	0.00	NA	+	NA	NA	NA	NA	NA	NA	NA	NA	7	-1009.17	2.80	0.03
4	0.08	0.00	0.00	NA	NA	NA	NA	NA	NA	NA	NA	NA	7	-1008.99	2.99	0.02
72	0.08	0.00	0.00	+	NA	NA	NA	+	NA	NA	NA	NA	9	-1008.69	3.29	0.02
24	0.09	-0.01	0.00	+	NA	+	NA	NA	NA	NA	NA	NA	9	-1008.68	3.30	0.02
79	0.08	NA	0.00	+	+	NA	NA	+	NA	NA	NA	NA	9	-1008.67	3.31	0.02
139	0.07	NA	0.00	NA	+	NA	NA	NA	+	NA	NA	NA	8	-1008.61	3.36	0.02
30	0.08	0.00	NA	+	+	+	NA	NA	NA	NA	NA	NA	9	-1008.36	3.61	0.02
172	0.10	-0.01	0.00	NA	+	NA	+	NA	+	NA	NA	NA	10	-1008.07	3.91	0.02
15	0.07	NA	0.00	+	+	NA	NA	NA	NA	NA	NA	NA	8	-1008.01	3.97	0.01
14	0.07	0.00	NA	+	+	NA	NA	NA	NA	NA	NA	NA	8	-1007.80	4.17	0.01
269	0.07	NA	NA	+	+	NA	NA	NA	NA	+	NA	NA	8	-1007.71	4.26	0.01
42	0.07	0.00	NA	NA	+	NA	+	NA	NA	NA	NA	NA	8	-1007.67	4.30	0.01
12	0.08	0.00	0.00	NA	+	NA	NA	NA	NA	NA	NA	NA	8	-1007.63	4.35	0.01
8	0.07	0.00	0.00	+	NA	NA	NA	NA	NA	NA	NA	NA	8	-1007.57	4.40	0.01
207	0.07	NA	0.00	+	+	NA	NA	+	+	NA	NA	NA	10	-1007.31	4.67	0.01
80	0.09	0.00	0.00	+	+	NA	NA	+	NA	NA	NA	NA	10	-1006.99	4.99	0.01
143	0.07	NA	0.00	+	+	NA	NA	NA	+	NA	NA	NA	9	-1006.97	5.00	0.01
32	0.09	-0.01	0.00	+	+	+	NA	NA	NA	NA	NA	NA	10	-1006.95	5.02	0.01
192	0.11	-0.01	0.00	+	+	+	+	NA	+	NA	NA	NA	12	-1006.80	5.18	0.01
335	0.08	NA	0.00	+	+	NA	NA	+	NA	+	NA	NA	10	-1006.69	5.29	0.01
176	0.10	-0.01	0.00	+	+	NA	+	NA	+	NA	NA	NA	11	-1006.69	5.29	0.01
88	0.09	-0.01	0.00	+	NA	+	NA	+	NA	NA	NA	NA	10	-1006.66	5.32	0.01
240	0.10	-0.01	0.00	+	+	NA	+	+	+	NA	NA	NA	12	-1006.64	5.33	0.01
140	0.07	0.00	0.00	NA	+	NA	NA	NA	+	NA	NA	NA	9	-1006.59	5.38	0.01
62	0.08	0.00	NA	+	+	+	+	NA	NA	NA	NA	NA	10	-1006.51	5.47	0.01
286	0.08	0.00	NA	+	+	+	NA	NA	NA	+	NA	NA	10	-1006.40	5.58	0.01
271	0.07	NA	0.00	+	+	NA	NA	NA	NA	+	NA	NA	9	-1006.27	5.71	0.01
16	0.08	0.00	0.00	+	+	NA	NA	NA	NA	NA	NA	NA	9	-1006.14	5.83	0.01
270	0.07	0.00	NA	+	+	NA	NA	NA	NA	+	NA	NA	9	-1006.07	5.91	0.01
46	0.07	0.00	NA	+	+	NA	+	NA	NA	NA	NA	NA	9	-1006.06	5.92	0.01
44	0.08	0.00	0.00	NA	+	NA	+	NA	NA	NA	NA	NA	9	-1005.84	6.14	0.00
960	0.12	-0.02	0.00	+	+	+	+	NA	+	+	+	NA	14	-1005.56	6.42	0.00
160	0.09	-0.01	0.00	+	+	+	NA	NA	+	NA	NA	NA	11	-1005.46	6.52	0.00
208	0.08	0.00	0.00	+	+	NA	NA	+	+	NA	NA	NA	11	-1005.43	6.54	0.00
1520	0.11	-0.01	0.00	+	+	NA	+	+	+	+	NA	+	14	-1005.39	6.58	0.00
463	0.07	NA	0.00	+	+	NA	NA	+	+	+	NA	NA	11	-1005.39	6.59	0.00
399	0.07	NA	0.00	+	+	NA	NA	NA	+	+	NA	NA	10	-1005.34	6.64	0.00
432	0.10	-0.01	0.00	+	+	NA	+	NA	+	+	NA	NA	12	-1005.24	6.74	0.00
336	0.09	0.00	0.00	+	+	NA	NA	+	NA	+	NA	NA	11	-1005.04	6.94	0.00
448	0.11	-0.01	0.00	+	+	+	+	NA	+	+	NA	NA	13	-1005.02	6.95	0.00
288	0.09	-0.01	0.00	+	+	+	NA	NA	NA	+	NA	NA	11	-1005.01	6.97	0.00
112	0.08	0.00	0.00	+	+	NA	+	+	NA	NA	NA	NA	11	-1004.98	7.00	0.00
144	0.07	0.00	0.00	+	+	NA	NA	NA	+	NA	NA	NA	10	-1004.96	7.01	0.00
64	0.09	-0.01	0.00	+	+	+	+	NA	NA	NA	NA	NA	11	-1004.96	7.02	0.00
96	0.09	-0.01	0.00	+	+	+	NA	+	NA	NA	NA	NA	11	-1004.94	7.04	0.00
496	0.10	-0.01	0.00	+	+	NA	+	+	+	+	NA	NA	13	-1004.86	7.11	0.00
1487	0.07	NA	0.00	+	+	NA	NA	+	+	+	NA	+	12	-1004.78	7.19	0.00
256	0.11	-0.01	0.00	+	+	+	+	+	+	NA	NA	NA	13	-1004.71	7.27	0.00
318	0.08	0.00	NA	+	+	+	+	NA	NA	+	NA	NA	11	-1004.56	7.41	0.00
272	0.08	0.00	0.00	+	+	NA	NA	NA	NA	+	NA	NA	10	-1004.45	7.52	0.00
302	0.06	0.00	NA	+	+	NA	+	NA	NA	+	NA	NA	10	-1004.36	7.62	0.00
48	0.07	0.00	0.00	+	+	NA	+	NA	NA	NA	NA	NA	10	-1004.27	7.71	0.00
1536	0.12	-0.02	0.00	+	+	+	+	+	+	+	NA	+	15	-1004.14	7.83	0.00
830	0.08	0.00	NA	+	+	+	+	NA	NA	+	+	NA	12	-1004.07	7.91	0.00
1024	0.13	-0.02	0.00	+	+	+	+	+	+	+	+	NA	15	-1003.80	8.17	0.00
416	0.09	-0.01	0.00	+	+	+	NA	NA	+	+	NA	NA	12	-1003.56	8.41	0.00
464	0.08	0.00	0.00	+	+	NA	NA	+	+	+	NA	NA	12	-1003.53	8.45	0.00
224	0.08	0.00	0.00	+	+	+	NA	+	+	NA	NA	NA	12	-1003.40	8.58	0.00
400	0.07	0.00	0.00	+	+	NA	NA	NA	+	+	NA	NA	11	-1003.35	8.63	0.00
368	0.08	0.00	0.00	+	+	NA	+	+	NA	+	NA	NA	12	-1003.03	8.94	0.00
320	0.09	-0.01	0.00	+	+	+	+	NA	NA	+	NA	NA	12	-1003.02	8.95	0.00
352	0.09	-0.01	0.00	+	+	+	NA	+	NA	+	NA	NA	12	-1002.98	9.00	0.00
512	0.11	-0.02	0.00	+	+	+	+	+	+	+	NA	NA	14	-1002.93	9.04	0.00
128	0.09	0.00	0.00	+	+	+	+	+	NA	NA	NA	NA	12	-1002.92	9.05	0.00
1488	0.08	0.00	0.00	+	+	NA	NA	+	+	+	NA	+	13	-1002.78	9.19	0.00
304	0.07	0.00	0.00	+	+	NA	+	NA	NA	+	NA	NA	11	-1002.60	9.38	0.00
832	0.09	-0.01	0.00	+	+	+	+	NA	NA	+	+	NA	13	-1002.35	9.63	0.00
2048	0.12	-0.02	0.00	+	+	+	+	+	+	+	+	+	16	-1002.06	9.91	0.00
480	0.08	0.00	0.00	+	+	+	NA	+	+	+	NA	NA	13	-1001.49	10.49	0.00
1504	0.09	-0.01	0.00	+	+	+	NA	+	+	+	NA	+	14	-1000.99	10.99	0.00
384	0.09	0.00	0.00	+	+	+	+	+	NA	+	NA	NA	13	-1000.97	11.00	0.00
896	0.09	-0.01	0.00	+	+	+	+	+	NA	+	+	NA	14	-1000.26	11.71	0.00

EOSINOPHIL COUNT

	I	AL	P	S	AL:P	AL:S	P:S	AL:P:S	NA	NA	NA	NA	df	AIC	delta	weight
6	0.14	0.00	NA	+	NA	NA	NA	NA	NA	NA	NA	NA	7	-689.27	0.00	0.27
8	0.14	0.00	+	+	NA	NA	NA	NA	NA	NA	NA	NA	8	-687.61	1.67	0.12
5	0.12	NA	NA	+	NA	NA	NA	NA	NA	NA	NA	NA	6	-687.43	1.85	0.11
22	0.14	0.00	NA	+	NA	+	NA	NA	NA	NA	NA	NA	8	-687.25	2.03	0.10
40	0.14	0.00	+	+	NA	NA	+	NA	NA	NA	NA	NA	9	-686.83	2.44	0.08
16	0.13	0.00	+	+	+	NA	NA	NA	NA	NA	NA	NA	9	-686.44	2.83	0.07
7	0.11	NA	+	+	NA	NA	NA	NA	NA	NA	NA	NA	7	-685.79	3.48	0.05
24	0.14	0.00	+	+	NA	+	NA	NA	NA	NA	NA	NA	9	-685.55	3.72	0.04
39	0.12	NA	+	+	NA	NA	+	NA	NA	NA	NA	NA	8	-685.55	3.72	0.04
48	0.13	0.00	+	+	+	NA	+	NA	NA	NA	NA	NA	10	-685.36	3.91	0.04
56	0.14	0.00	+	+	NA	+	+	NA	NA	NA	NA	NA	10	-684.76	4.51	0.03
32	0.13	0.00	+	+	+	+	NA	NA	NA	NA	NA	NA	10	-684.40	4.87	0.02
64	0.13	0.00	+	+	+	+	+	NA	NA	NA	NA	NA	11	-683.30	5.98	0.01
128	0.14	0.00	+	+	+	+	+	+	NA	NA	NA	NA	12	-681.92	7.36	0.01
2	0.12	0.00	NA	NA	NA	NA	NA	NA	NA	NA	NA	NA	6	-681.57	7.70	0.01
1	0.10	NA	NA	NA	NA	NA	NA	NA	NA	NA	NA	NA	5	-681.22	8.06	0.00
4	0.11	0.00	+	NA	NA	NA	NA	NA	NA	NA	NA	NA	7	-680.09	9.18	0.00
3	0.10	NA	+	NA	NA	NA	NA	NA	NA	NA	NA	NA	6	-679.76	9.51	0.00
12	0.11	0.00	+	NA	+	NA	NA	NA	NA	NA	NA	NA	8	-678.41	10.86	0.00
	I	AF	P	S	AF:P	AF:S	P:S	AF:P:S	NA	NA	NA	NA	df	AIC	delta	weight
5	0.12	NA	NA	+	NA	NA	NA	NA	NA	NA	NA	NA	6	-687.43	0.00	0.53
7	0.11	NA	+	+	NA	NA	NA	NA	NA	NA	NA	NA	7	-685.79	1.64	0.23
39	0.12	NA	+	+	NA	NA	+	NA	NA	NA	NA	NA	8	-685.55	1.88	0.21
1	0.10	NA	NA	NA	NA	NA	NA	NA	NA	NA	NA	NA	5	-681.22	6.21	0.02
3	0.10	NA	+	NA	NA	NA	NA	NA	NA	NA	NA	NA	6	-679.76	7.66	0.01
6	0.14	+	NA	+	NA	NA	NA	NA	NA	NA	NA	NA	16	-672.79	14.63	0.00
8	0.13	+	+	+	NA	NA	NA	NA	NA	NA	NA	NA	17	-671.10	16.32	0.00
40	0.14	+	+	+	NA	NA	+	NA	NA	NA	NA	NA	18	-670.23	17.20	0.00
16	0.11	+	+	+	+	NA	NA	NA	NA	NA	NA	NA	27	-665.43	22.00	0.00
2	0.12	+	NA	NA	NA	NA	NA	NA	NA	NA	NA	NA	15	-665.10	22.32	0.00
48	0.12	+	+	+	+	NA	+	NA	NA	NA	NA	NA	28	-664.68	22.75	0.00
4	0.11	+	+	NA	NA	NA	NA	NA	NA	NA	NA	NA	16	-663.62	23.80	0.00
22	0.14	+	NA	+	NA	+	NA	NA	NA	NA	NA	NA	26	-662.69	24.73	0.00
24	0.14	+	+	+	NA	+	NA	NA	NA	NA	NA	NA	27	-660.77	26.66	0.00
56	0.15	+	+	+	NA	+	+	NA	NA	NA	NA	NA	28	-659.94	27.49	0.00
12	0.10	+	+	NA	+	NA	NA	NA	NA	NA	NA	NA	26	-657.28	30.14	0.00
32	0.12	+	+	+	+	+	NA	NA	NA	NA	NA	NA	37	-654.97	32.45	0.00
64	0.13	+	+	+	+	+	+	NA	NA	NA	NA	NA	38	-654.32	33.11	0.00
128	0.12	+	+	+	+	+	+	+	NA	NA	NA	NA	46	-639.00	48.42	0.00
	I	AT	P	S	AT:P	AT:S	P:S	AT:P:S	NA	NA	NA	NA	df	AIC	delta	weight
5	0.12	NA	NA	+	NA	NA	NA	NA	NA	NA	NA	NA	6	-687.43	0.00	0.29
6	0.19	-0.01	NA	+	NA	NA	NA	NA	NA	NA	NA	NA	7	-686.03	1.39	0.14
7	0.11	NA	+	+	NA	NA	NA	NA	NA	NA	NA	NA	7	-685.79	1.64	0.13
39	0.12	NA	+	+	NA	NA	+	NA	NA	NA	NA	NA	8	-685.55	1.88	0.11
8	0.18	-0.01	+	+	NA	NA	NA	NA	NA	NA	NA	NA	8	-684.32	3.11	0.06
22	0.18	-0.01	NA	+	NA	+	NA	NA	NA	NA	NA	NA	8	-684.06	3.36	0.05
40	0.19	-0.01	+	+	NA	NA	+	NA	NA	NA	NA	NA	9	-684.05	3.38	0.05
48	0.24	-0.01	+	+	+	NA	+	NA	NA	NA	NA	NA	10	-682.82	4.61	0.03
16	0.22	-0.01	+	+	+	NA	NA	NA	NA	NA	NA	NA	9	-682.77	4.66	0.03
24	0.17	-0.01	+	+	NA	+	NA	NA	NA	NA	NA	NA	9	-682.33	5.10	0.02
56	0.18	-0.01	+	+	NA	+	+	NA	NA	NA	NA	NA	10	-681.99	5.43	0.02
1	0.10	NA	NA	NA	NA	NA	NA	NA	NA	NA	NA	NA	5	-681.22	6.21	0.01
64	0.25	-0.01	+	+	+	+	+	NA	NA	NA	NA	NA	11	-680.77	6.65	0.01
32	0.22	-0.01	+	+	+	+	NA	NA	NA	NA	NA	NA	10	-680.70	6.72	0.01
3	0.10	NA	+	NA	NA	NA	NA	NA	NA	NA	NA	NA	6	-679.76	7.66	0.01
128	0.27	-0.02	+	+	+	+	+	+	NA	NA	NA	NA	12	-679.48	7.95	0.01
2	0.14	0.00	NA	NA	NA	NA	NA	NA	NA	NA	NA	NA	6	-679.37	8.06	0.01
4	0.13	0.00	+	NA	NA	NA	NA	NA	NA	NA	NA	NA	7	-677.86	9.56	0.00
12	0.18	-0.01	+	NA	+	NA	NA	NA	NA	NA	NA	NA	8	-676.53	10.90	0.00
	I	AL	I(AL^2)	P	S	AL:P	AL:S	I(AL^2):P	I(AL^2):S	P:S	AL:P:S	I(AL^2):P:S	df	AIC	delta	weight
10	0.14	0.00	NA	NA	+	NA	NA	NA	NA	NA	NA	NA	7	-689.27	0.00	0.12
11	0.13	NA	0.00	NA	+	NA	NA	NA	NA	NA	NA	NA	7	-688.36	0.91	0.08
12	0.16	-0.01	0.00	NA	+	NA	NA	NA	NA	NA	NA	NA	8	-687.87	1.41	0.06
14	0.14	0.00	NA	+	+	NA	NA	NA	NA	NA	NA	NA	8	-687.61	1.67	0.05
9	0.12	NA	NA	NA	+	NA	NA	NA	NA	NA	NA	NA	6	-687.43	1.85	0.05
42	0.14	0.00	NA	NA	+	NA	+	NA	NA	NA	NA	NA	8	-687.25	2.03	0.04
270	0.14	0.00	NA	+	+	NA	NA	NA	NA	+	NA	NA	9	-686.83	2.44	0.04
15	0.12	NA	0.00	+	+	NA	NA	NA	NA	NA	NA	NA	8	-686.67	2.60	0.03
30	0.13	0.00	NA	+	+	+	NA	NA	NA	NA	NA	NA	9	-686.44	2.83	0.03
139	0.13	NA	0.00	NA	+	NA	NA	NA	+	NA	NA	NA	8	-686.42	2.85	0.03
16	0.15	-0.01	0.00	+	+	NA	NA	NA	NA	NA	NA	NA	9	-686.25	3.03	0.03
271	0.13	NA	0.00	+	+	NA	NA	NA	NA	+	NA	NA	9	-686.04	3.24	0.02
140	0.15	-0.01	0.00	NA	+	NA	NA	NA	+	NA	NA	NA	9	-685.81	3.46	0.02
44	0.16	-0.01	0.00	NA	+	NA	+	NA	NA	NA	NA	NA	9	-685.81	3.47	0.02
13	0.11	NA	NA	+	+	NA	NA	NA	NA	NA	NA	NA	7	-685.79	3.48	0.02
46	0.14	0.00	NA	+	+	NA	+	NA	NA	NA	NA	NA	9	-685.55	3.72	0.02

269	0.12	NA	NA	+	+	NA	NA	NA	NA	+	NA	NA	8	-685.55	3.72	0.02
286	0.13	0.00	NA	+	+	+	NA	NA	NA	+	NA	NA	10	-685.36	3.91	0.02
272	0.15	-0.01	0.00	+	+	NA	NA	NA	NA	+	NA	NA	10	-685.34	3.94	0.02
79	0.12	NA	0.00	+	+	NA	NA	+	NA	NA	NA	NA	9	-685.01	4.26	0.01
96	0.12	0.00	0.00	+	+	+	NA	+	NA	NA	NA	NA	11	-685.01	4.27	0.01
32	0.14	-0.01	0.00	+	+	+	NA	NA	NA	NA	NA	NA	10	-684.92	4.35	0.01
302	0.14	0.00	NA	+	+	NA	+	NA	NA	+	NA	NA	10	-684.76	4.51	0.01
143	0.12	NA	0.00	+	+	NA	NA	NA	+	NA	NA	NA	9	-684.69	4.59	0.01
80	0.15	-0.01	0.00	+	+	NA	NA	+	NA	NA	NA	NA	10	-684.42	4.85	0.01
62	0.13	0.00	NA	+	+	+	+	NA	NA	NA	NA	NA	10	-684.40	4.87	0.01
48	0.15	-0.01	0.00	+	+	NA	+	NA	NA	NA	NA	NA	10	-684.18	5.09	0.01
144	0.15	-0.01	0.00	+	+	NA	NA	NA	+	NA	NA	NA	10	-684.18	5.10	0.01
335	0.13	NA	0.00	+	+	NA	NA	+	NA	+	NA	NA	10	-684.16	5.12	0.01
352	0.12	0.00	0.00	+	+	+	NA	+	NA	+	NA	NA	12	-684.02	5.26	0.01
399	0.13	NA	0.00	+	+	NA	NA	NA	+	+	NA	NA	10	-684.00	5.27	0.01
172	0.16	-0.01	0.00	NA	+	NA	+	NA	+	NA	NA	NA	10	-683.88	5.39	0.01
288	0.15	-0.01	0.00	+	+	+	NA	NA	NA	+	NA	NA	11	-683.76	5.52	0.01
336	0.15	-0.01	0.00	+	+	NA	NA	+	NA	+	NA	NA	11	-683.37	5.91	0.01
318	0.13	0.00	NA	+	+	+	+	NA	NA	+	NA	NA	11	-683.30	5.98	0.01
304	0.16	-0.01	0.00	+	+	NA	+	NA	NA	+	NA	NA	11	-683.28	5.99	0.01
400	0.16	-0.01	0.00	+	+	NA	NA	NA	+	+	NA	NA	11	-683.26	6.01	0.01
207	0.12	NA	0.00	+	+	NA	NA	+	+	NA	NA	NA	10	-683.07	6.20	0.01
128	0.12	0.00	0.00	+	+	+	+	+	NA	NA	NA	NA	12	-682.94	6.33	0.01
224	0.12	0.00	0.00	+	+	+	NA	+	+	NA	NA	NA	12	-682.93	6.35	0.01
160	0.14	-0.01	0.00	+	+	+	NA	NA	+	NA	NA	NA	11	-682.86	6.42	0.00
64	0.14	-0.01	0.00	+	+	+	+	NA	NA	NA	NA	NA	11	-682.84	6.43	0.00
208	0.15	-0.01	0.00	+	+	NA	NA	+	+	NA	NA	NA	11	-682.36	6.92	0.00
112	0.15	-0.01	0.00	+	+	NA	+	+	NA	NA	NA	NA	11	-682.35	6.92	0.00
176	0.16	-0.01	0.00	+	+	NA	+	NA	+	NA	NA	NA	11	-682.29	6.99	0.00
463	0.13	NA	0.00	+	+	NA	NA	+	+	+	NA	NA	11	-682.14	7.13	0.00
384	0.12	0.00	0.00	+	+	+	+	+	NA	+	NA	NA	13	-681.97	7.30	0.00
480	0.12	0.00	0.00	+	+	+	NA	+	+	+	NA	NA	13	-681.95	7.32	0.00
830	0.14	0.00	NA	+	+	+	+	NA	NA	+	+	NA	12	-681.92	7.36	0.00
320	0.15	-0.01	0.00	+	+	+	+	NA	NA	+	NA	NA	12	-681.68	7.60	0.00
416	0.15	-0.01	0.00	+	+	+	NA	NA	+	+	NA	NA	12	-681.68	7.60	0.00
1487	0.13	NA	0.00	+	+	NA	NA	+	+	+	NA	+	12	-681.58	7.70	0.00
2	0.12	0.00	NA	NA	NA	NA	NA	NA	NA	NA	NA	NA	6	-681.57	7.70	0.00
432	0.16	-0.01	0.00	+	+	NA	+	NA	+	+	NA	NA	12	-681.32	7.95	0.00
368	0.15	-0.01	0.00	+	+	NA	+	+	NA	+	NA	NA	12	-681.29	7.98	0.00
464	0.15	-0.01	0.00	+	+	NA	NA	+	+	+	NA	NA	12	-681.28	7.99	0.00
1	0.10	NA	NA	NA	NA	NA	NA	NA	NA	NA	NA	NA	5	-681.22	8.06	0.00
192	0.15	-0.01	0.00	+	+	+	+	NA	+	NA	NA	NA	12	-681.01	8.26	0.00
256	0.12	0.00	0.00	+	+	+	+	+	+	NA	NA	NA	13	-680.95	8.33	0.00
3	0.11	NA	0.00	NA	NA	NA	NA	NA	NA	NA	NA	NA	6	-680.81	8.46	0.00
240	0.16	-0.01	0.00	+	+	NA	+	+	+	NA	NA	NA	12	-680.50	8.77	0.00
4	0.14	-0.01	0.00	NA	NA	NA	NA	NA	NA	NA	NA	NA	7	-680.42	8.85	0.00
1488	0.15	-0.01	0.00	+	+	NA	NA	+	+	+	NA	+	13	-680.37	8.91	0.00
1504	0.13	0.00	0.00	+	+	+	NA	+	+	+	NA	+	14	-680.31	8.96	0.00
832	0.15	-0.01	0.00	+	+	+	+	NA	NA	+	+	NA	13	-680.19	9.09	0.00
6	0.11	0.00	NA	+	NA	NA	NA	NA	NA	NA	NA	NA	7	-680.09	9.18	0.00
896	0.13	0.00	0.00	+	+	+	+	+	NA	+	+	NA	14	-680.03	9.24	0.00
512	0.13	0.00	0.00	+	+	+	+	+	+	+	NA	NA	14	-679.92	9.35	0.00
448	0.15	-0.01	0.00	+	+	+	+	NA	+	+	NA	NA	13	-679.77	9.50	0.00
5	0.10	NA	NA	+	NA	NA	NA	NA	NA	NA	NA	NA	6	-679.76	9.51	0.00
496	0.16	-0.01	0.00	+	+	NA	+	+	+	+	NA	NA	13	-679.36	9.91	0.00
7	0.11	NA	0.00	+	NA	NA	NA	NA	NA	NA	NA	NA	7	-679.32	9.96	0.00
8	0.13	-0.01	0.00	+	NA	NA	NA	NA	NA	NA	NA	NA	8	-679.01	10.26	0.00
1520	0.16	-0.01	0.00	+	+	NA	+	+	+	+	NA	+	14	-678.73	10.55	0.00
960	0.16	-0.01	0.00	+	+	+	+	NA	+	+	+	NA	14	-678.44	10.83	0.00
22	0.11	0.00	NA	+	NA	+	NA	NA	NA	NA	NA	NA	8	-678.41	10.86	0.00
1536	0.13	0.00	0.00	+	+	+	+	+	+	+	NA	+	15	-678.41	10.86	0.00
1024	0.13	0.00	0.00	+	+	+	+	+	+	+	+	NA	15	-678.03	11.24	0.00
2048	0.12	0.01	0.00	+	+	+	+	+	+	+	+	+	16	-677.80	11.48	0.00
71	0.10	NA	0.00	+	NA	NA	NA	+	NA	NA	NA	NA	8	-677.37	11.90	0.00
24	0.13	-0.01	0.00	+	NA	+	NA	NA	NA	NA	NA	NA	9	-677.22	12.06	0.00
72	0.13	-0.01	0.00	+	NA	NA	NA	+	NA	NA	NA	NA	9	-676.99	12.28	0.00
88	0.11	0.00	0.00	+	NA	+	NA	+	NA	NA	NA	NA	10	-676.89	12.38	0.00

LYMPHOCYTE COUNT

	I	AL	P	S	AL:P	AL:S	P:S	AL:P:S	NA	NA	NA	NA	df	AIC	delta	weight
4	2.56	-0.04	+	NA	NA	NA	NA	NA	NA	NA	NA	NA	7	1489.61	0.00	0.17
12	2.48	-0.02	+	NA	+	NA	NA	NA	NA	NA	NA	NA	8	1489.81	0.20	0.15
16	2.53	-0.02	+	+	+	NA	NA	NA	NA	NA	NA	NA	9	1489.91	0.30	0.14
8	2.62	-0.04	+	+	NA	NA	NA	NA	NA	NA	NA	NA	8	1490.14	0.53	0.13
40	2.64	-0.04	+	+	NA	NA	+	NA	NA	NA	NA	NA	9	1490.87	1.26	0.09
48	2.56	-0.02	+	+	+	NA	+	NA	NA	NA	NA	NA	10	1491.09	1.48	0.08
32	2.49	-0.01	+	+	+	+	NA	NA	NA	NA	NA	NA	10	1491.13	1.52	0.08
24	2.58	-0.03	+	+	NA	+	NA	NA	NA	NA	NA	NA	9	1491.53	1.93	0.06
56	2.61	-0.03	+	+	NA	+	+	NA	NA	NA	NA	NA	10	1492.38	2.77	0.04
64	2.52	-0.01	+	+	+	+	+	NA	NA	NA	NA	NA	11	1492.44	2.83	0.04
128	2.50	-0.01	+	+	+	+	+	+	NA	NA	NA	NA	12	1494.41	4.80	0.01
3	2.36	NA	+	NA	NA	NA	NA	NA	NA	NA	NA	NA	6	1496.03	6.43	0.01
39	2.44	NA	+	+	NA	NA	+	NA	NA	NA	NA	NA	8	1497.32	7.72	0.00
7	2.39	NA	+	+	NA	NA	NA	NA	NA	NA	NA	NA	7	1497.42	7.81	0.00
2	2.25	-0.04	NA	NA	NA	NA	NA	NA	NA	NA	NA	NA	6	1549.96	60.35	0.00
6	2.29	-0.04	NA	+	NA	NA	NA	NA	NA	NA	NA	NA	7	1551.21	61.60	0.00
22	2.30	-0.04	NA	+	NA	+	NA	NA	NA	NA	NA	NA	8	1553.26	63.65	0.00
1	2.06	NA	NA	NA	NA	NA	NA	NA	NA	NA	NA	NA	5	1554.21	64.61	0.00
5	2.09	NA	NA	+	NA	NA	NA	NA	NA	NA	NA	NA	6	1555.77	66.17	0.00
	I	AF	P	S	AF:P	AF:S	P:S	AF:P:S	NA	NA	NA	NA	df	AIC	delta	weight
4	2.64	+	+	NA	NA	NA	NA	NA	NA	NA	NA	NA	16	1488.82	0.00	0.45
8	2.69	+	+	+	NA	NA	NA	NA	NA	NA	NA	NA	17	1489.48	0.66	0.32
40	2.72	+	+	+	NA	NA	+	NA	NA	NA	NA	NA	18	1490.48	1.66	0.19
3	2.36	NA	+	NA	NA	NA	NA	NA	NA	NA	NA	NA	6	1496.03	7.22	0.01
12	2.57	+	+	NA	+	NA	NA	NA	NA	NA	NA	NA	26	1496.98	8.16	0.01
39	2.44	NA	+	+	NA	NA	+	NA	NA	NA	NA	NA	8	1497.32	8.51	0.01
7	2.39	NA	+	+	NA	NA	NA	NA	NA	NA	NA	NA	7	1497.42	8.60	0.01
16	2.61	+	+	+	+	NA	NA	NA	NA	NA	NA	NA	27	1497.66	8.85	0.01
48	2.64	+	+	+	+	NA	+	NA	NA	NA	NA	NA	28	1499.00	10.18	0.00
24	2.69	+	+	+	NA	+	NA	NA	NA	NA	NA	NA	27	1504.90	16.08	0.00
56	2.72	+	+	+	NA	+	+	NA	NA	NA	NA	NA	28	1505.93	17.12	0.00
32	2.61	+	+	+	+	+	NA	NA	NA	NA	NA	NA	37	1516.08	27.26	0.00
64	2.64	+	+	+	+	+	+	NA	NA	NA	NA	NA	38	1517.47	28.66	0.00
128	2.72	+	+	+	+	+	+	+	NA	NA	NA	NA	46	1520.39	31.57	0.00
2	2.35	+	NA	NA	NA	NA	NA	NA	NA	NA	NA	NA	15	1548.42	59.60	0.00
6	2.38	+	NA	+	NA	NA	NA	NA	NA	NA	NA	NA	16	1549.77	60.95	0.00
1	2.06	NA	NA	NA	NA	NA	NA	NA	NA	NA	NA	NA	5	1554.21	65.40	0.00
5	2.09	NA	NA	+	NA	NA	NA	NA	NA	NA	NA	NA	6	1555.77	66.96	0.00
22	2.41	+	NA	+	NA	+	NA	NA	NA	NA	NA	NA	26	1567.05	78.23	0.00
	I	AT	P	S	AT:P	AT:S	P:S	AT:P:S	NA	NA	NA	NA	df	AIC	delta	weight
3	2.36	NA	+	NA	NA	NA	NA	NA	NA	NA	NA	NA	6	1496.03	0.00	0.28
39	2.44	NA	+	+	NA	NA	+	NA	NA	NA	NA	NA	8	1497.32	1.29	0.15
7	2.39	NA	+	+	NA	NA	NA	NA	NA	NA	NA	NA	7	1497.42	1.38	0.14
4	1.42	0.09	+	NA	NA	NA	NA	NA	NA	NA	NA	NA	7	1497.92	1.89	0.11
12	0.02	0.21	+	NA	+	NA	NA	NA	NA	NA	NA	NA	8	1499.03	2.99	0.06
40	1.70	0.07	+	+	NA	NA	+	NA	NA	NA	NA	NA	9	1499.29	3.25	0.06
8	1.59	0.07	+	+	NA	NA	NA	NA	NA	NA	NA	NA	8	1499.36	3.32	0.05
16	0.18	0.20	+	+	+	NA	NA	NA	NA	NA	NA	NA	9	1500.43	4.39	0.03
48	0.42	0.18	+	+	+	NA	+	NA	NA	NA	NA	NA	10	1500.56	4.53	0.03
56	2.54	-0.01	+	+	NA	+	+	NA	NA	NA	NA	NA	10	1500.83	4.80	0.03
24	2.28	0.01	+	+	NA	+	NA	NA	NA	NA	NA	NA	9	1501.07	5.03	0.02
32	0.70	0.15	+	+	+	+	NA	NA	NA	NA	NA	NA	10	1502.40	6.37	0.01
64	1.22	0.11	+	+	+	+	+	NA	NA	NA	NA	NA	11	1502.41	6.38	0.01
128	1.22	0.11	+	+	+	+	+	+	NA	NA	NA	NA	11	1502.41	6.38	0.01
1	2.06	NA	NA	NA	NA	NA	NA	NA	NA	NA	NA	NA	5	1554.21	58.18	0.00
2	-0.41	0.22	NA	NA	NA	NA	NA	NA	NA	NA	NA	NA	6	1555.30	59.27	0.00
5	2.09	NA	NA	+	NA	NA	NA	NA	NA	NA	NA	NA	6	1555.77	59.74	0.00
6	-0.32	0.22	NA	+	NA	NA	NA	NA	NA	NA	NA	NA	7	1556.92	60.89	0.00
22	0.51	0.14	NA	+	NA	+	NA	NA	NA	NA	NA	NA	8	1558.54	62.50	0.00
	I	AL	I(AL^2)	P	S	AL:P	AL:S	I(AL^2):P	I(AL^2):S	P:S	AL:P:S	I(AL^2):P:S	df	AIC	delta	weight
8	2.82	-0.15	0.01	+	NA	NA	NA	NA	NA	NA	NA	NA	8	1487.20	0.00	0.09
24	2.73	-0.13	0.01	+	NA	+	NA	NA	NA	NA	NA	NA	9	1487.87	0.67	0.07
16	2.86	-0.15	0.01	+	+	NA	NA	NA	NA	NA	NA	NA	9	1487.96	0.76	0.06
72	2.77	-0.14	0.01	+	NA	NA	NA	+	NA	NA	NA	NA	9	1488.01	0.81	0.06
32	2.77	-0.13	0.01	+	+	+	NA	NA	NA	NA	NA	NA	10	1488.27	1.07	0.05
80	2.81	-0.14	0.01	+	+	NA	NA	+	NA	NA	NA	NA	10	1488.47	1.27	0.05
272	2.88	-0.14	0.01	+	+	NA	NA	NA	NA	+	NA	NA	10	1488.99	1.79	0.04
6	2.56	-0.04	NA	+	NA	NA	NA	NA	NA	NA	NA	NA	7	1489.61	2.41	0.03
288	2.79	-0.13	0.01	+	+	+	NA	NA	NA	+	NA	NA	11	1489.64	2.44	0.03
22	2.48	-0.02	NA	+	NA	+	NA	NA	NA	NA	NA	NA	8	1489.81	2.61	0.02
336	2.83	-0.14	0.01	+	+	NA	NA	+	NA	+	NA	NA	11	1489.84	2.64	0.02
48	2.83	-0.14	0.01	+	+	NA	+	NA	NA	NA	NA	NA	10	1489.88	2.69	0.02
30	2.53	-0.02	NA	+	+	+	NA	NA	NA	NA	NA	NA	9	1489.91	2.71	0.02
88	2.72	-0.13	0.01	+	NA	+	NA	+	NA	NA	NA	NA	10	1489.92	2.72	0.02
144	2.84	-0.15	0.01	+	+	NA	NA	NA	+	NA	NA	NA	10	1489.94	2.74	0.02
64	2.73	-0.12	0.01	+	+	+	+	NA	NA	NA	NA	NA	11	1490.10	2.90	0.02
14	2.62	-0.04	NA	+	+	NA	NA	NA	NA	NA	NA	NA	8	1490.14	2.94	0.02
160	2.75	-0.12	0.01	+	+	+	NA	NA	+	NA	NA	NA	11	1490.15	2.95	0.02
112	2.77	-0.13	0.01	+	+	NA	+	+	NA	NA	NA	NA	11	1490.28	3.08	0.02
96	2.75	-0.12	0.01	+	+	+	NA	+	NA	NA	NA	NA	11	1490.32	3.12	0.02
208	2.78	-0.14	0.01	+	+	NA	NA	+	+	NA	NA	NA	11	1490.32	3.13	0.02
270	2.64	-0.04	NA	+	+	NA	NA	NA	NA	+	NA	NA	9	1490.87	3.67	0.01
304	2.85	-0.14	0.01	+	+	NA	+	NA	NA	+	NA	NA	11	1490.95	3.76	0.01
400	2.86	-0.14	0.01	+	+	NA	NA	NA	+	+	NA	NA	11	1491.01	3.81	0.01
286	2.56	-0.02	NA	+	+	+	NA	NA	NA	+	NA	NA	10	1491.09	3.89	0.01
62	2.49	-0.01	NA	+	+	+	+	NA	NA	NA	NA	NA	10	1491.13	3.93	0.01
320	2.76	-0.11	0.01	+	+	+	+	NA	NA	+	NA	NA	12	1491.52	4.32	0.01
46	2.58	-0.03	NA	+	+	NA	+	NA	NA	NA	NA	NA	9	1491.53	4.34	0.01
416	2.77	-0.12	0.01	+	+	+	NA	NA	+	+	NA	NA	12	1491.58	4.38	0.01
352	2.77	-0.12	0.01	+	+	+	NA	+	NA	+	NA	NA	12	1491.68	4.48	0.01
368	2.79	-0.13	0.01	+	+	NA	+	+	NA	+	NA	NA	12	1491.71	4.51	0.01
464	2.81	-0.13	0.01	+	+	NA	NA	+	+	+	NA	NA	12	1491.76	4.56	0.01
176	2.80	-0.13	0.01	+	+	NA	+	NA	+	NA	NA	NA	11	1491.88	4.68	0.01
192	2.72	-0.11	0.01	+	+	+	+	NA	+	NA	NA	NA	12	1492.15	4.96	0.01
128	2.72	-0.11	0.01	+	+	+	+	+	NA	NA	NA	NA	12	1492.16	4.97	0.01
224	2.73	-0.12	0.01	+	+	+	NA	+	+	NA	NA	NA	12	1492.22	5.02	0.01
240	2.76	-0.13	0.01	+	+	NA	+	+	+	NA	NA	NA	12	1492.35	5.15	0.01
302	2.61	-0.03	NA	+	+	NA	+	NA	NA	+	NA	NA	10	1492.38	5.18	0.01
318	2.52	-0.01	NA	+	+	+	+	NA	NA	+	NA	NA	11	1492.44	5.24	0.01
7	2.45	NA	0.00	+	NA	NA	NA	NA	NA	NA	NA	NA	7	1492.73	5.53	0.01
432	2.82	-0.12	0.01	+	+	NA	+	NA	+	+	NA	NA	12	1492.91	5.71	0.01
71	2.41	NA	0.00	+	NA	NA	NA	+	NA	NA	NA	NA	8	1492.93	5.73	0.01
79	2.46	NA	0.00	+	+	NA	NA	+	NA	NA	NA	NA	9	1493.25	6.05	0.00
832	2.74	-0.11	0.01	+	+	+	+	NA	NA	+	+	NA	13	1493.33	6.13	0.00
1488	2.81	-0.14	0.01	+	+	NA	NA	+	+	+	NA	+	13	1493.39	6.20	0.00
15	2.50	NA	0.00	+	+	NA	NA	NA	NA	NA	NA	NA	8	1493.42	6.22	0.00
448	2.74	-0.11	0.01	+	+	+	+	NA	+	+	NA	NA	13	1493.56	6.36	0.00
384	2.74	-0.11	0.01	+	+	+	+	+	NA	+	NA	NA	13	1493.58	6.38	0.00
480	2.75	-0.11	0.01	+	+	+	NA	+	+	+	NA	NA	13	1493.64	6.44	0.00
496	2.78	-0.12	0.01	+	+	NA	+	+	+	+	NA	NA	13	1493.76	6.56	0.00
271	2.53	NA	0.00	+	+	NA	NA	NA	NA	+	NA	NA	9	1493.89	6.69	0.00
256	2.70	-0.10	0.01	+	+	+	+	+	+	NA	NA	NA	13	1494.22	7.03	0.00
335	2.49	NA	0.00	+	+	NA	NA	+	NA	+	NA	NA	10	1494.24	7.04	0.00
207	2.43	NA	0.00	+	+	NA	NA	+	+	NA	NA	NA	10	1494.25	7.06	0.00
830	2.50	-0.01	NA	+	+	+	+	NA	NA	+	+	NA	12	1494.41	7.21	0.00
143	2.48	NA	0.00	+	+	NA	NA	NA	+	NA	NA	NA	9	1494.75	7.56	0.00
1504	2.73	-0.11	0.01	+	+	+	NA	+	+	+	NA	+	14	1495.06	7.87	0.00
1520	2.76	-0.12	0.01	+	+	NA	+	+	+	+	NA	+	14	1495.28	8.09	0.00
960	2.71	-0.10	0.01	+	+	+	+	NA	+	+	+	NA	14	1495.32	8.12	0.00
896	2.71	-0.10	0.01	+	+	+	+	+	NA	+	+	NA	14	1495.32	8.13	0.00
399	2.51	NA	0.00	+	+	NA	NA	NA	+	+	NA	NA	10	1495.39	8.19	0.00
463	2.46	NA	0.00	+	+	NA	NA	+	+	+	NA	NA	11	1495.45	8.25	0.00
512	2.71	-0.10	0.01	+	+	+	+	+	+	+	NA	NA	14	1495.61	8.42	0.00
5	2.36	NA	NA	+	NA	NA	NA	NA	NA	NA	NA	NA	6	1496.03	8.84	0.00
1536	2.66	-0.08	0.01	+	+	+	+	+	+	+	NA	+	15	1496.83	9.64	0.00
1024	2.66	-0.08	0.01	+	+	+	+	+	+	+	+	NA	15	1497.27	10.07	0.00
269	2.44	NA	NA	+	+	NA	NA	NA	NA	+	NA	NA	8	1497.32	10.13	0.00
13	2.39	NA	NA	+	+	NA	NA	NA	NA	NA	NA	NA	7	1497.42	10.22	0.00
1487	2.46	NA	0.00	+	+	NA	NA	+	+	+	NA	+	12	1497.44	10.24	0.00
2048	2.75	-0.12	0.01	+	+	+	+	+	+	+	+	+	16	1497.95	10.75	0.00
4	2.55	-0.17	0.01	NA	NA	NA	NA	NA	NA	NA	NA	NA	7	1546.48	59.28	0.00
12	2.58	-0.17	0.01	NA	+	NA	NA	NA	NA	NA	NA	NA	8	1547.91	60.71	0.00
44	2.63	-0.18	0.01	NA	+	NA	+	NA	NA	NA	NA	NA	9	1549.63	62.43	0.00
140	2.62	-0.17	0.01	NA	+	NA	NA	NA	+	NA	NA	NA	9	1549.64	62.45	0.00
2	2.25	-0.04	NA	NA	NA	NA	NA	NA	NA	NA	NA	NA	6	1549.96	62.77	0.00
10	2.29	-0.04	NA	NA	+	NA	NA	NA	NA	NA	NA	NA	7	1551.21	64.02	0.00
172	2.63	-0.18	0.01	NA	+	NA	+	NA	+	NA	NA	NA	10	1551.70	64.50	0.00
3	2.14	NA	0.00	NA	NA	NA	NA	NA	NA	NA	NA	NA	6	1552.97	65.77	0.00
42	2.30	-0.04	NA	NA	+	NA	+	NA	NA	NA	NA	NA	8	1553.26	66.06	0.00
1	2.06	NA	NA	NA	NA	NA	NA	NA	NA	NA	NA	NA	5	1554.21	67.02	0.00
11	2.17	NA	0.00	NA	+	NA	NA	NA	NA	NA	NA	NA	7	1554.34	67.15	0.00
9	2.09	NA	NA	NA	+	NA	NA	NA	NA	NA	NA	NA	6	1555.77	68.58	0.00
139	2.17	NA	0.00	NA	+	NA	NA	NA	+	NA	NA	NA	8	1556.40	69.20	0.00

ALPHA1-GLOBULIN

	I	AL	P	S	AL:P	AL:S	P:S	AL:P:S	NA	NA	NA	NA	df	AIC	delta	weight
40	3.13	0.02	+	+	NA	NA	+	NA	NA	NA	NA	NA	9	1095.77	0.00	0.20
56	3.09	0.03	+	+	NA	+	+	NA	NA	NA	NA	NA	10	1095.92	0.14	0.19
24	3.06	0.03	+	+	NA	+	NA	NA	NA	NA	NA	NA	9	1096.28	0.51	0.16
8	3.11	0.02	+	+	NA	NA	NA	NA	NA	NA	NA	NA	8	1096.50	0.73	0.14
48	3.14	0.02	+	+	+	NA	+	NA	NA	NA	NA	NA	10	1097.76	1.99	0.07
64	3.09	0.03	+	+	+	+	+	NA	NA	NA	NA	NA	11	1097.96	2.18	0.07
32	3.06	0.03	+	+	+	+	NA	NA	NA	NA	NA	NA	10	1098.34	2.56	0.06
16	3.11	0.02	+	+	+	NA	NA	NA	NA	NA	NA	NA	9	1098.56	2.79	0.05
128	3.11	0.03	+	+	+	+	+	+	NA	NA	NA	NA	12	1099.72	3.94	0.03
7	3.24	NA	+	+	NA	NA	NA	NA	NA	NA	NA	NA	7	1101.18	5.41	0.01
39	3.26	NA	+	+	NA	NA	+	NA	NA	NA	NA	NA	8	1101.38	5.60	0.01
6	3.04	0.02	NA	+	NA	NA	NA	NA	NA	NA	NA	NA	7	1102.32	6.54	0.01
22	3.00	0.03	NA	+	NA	+	NA	NA	NA	NA	NA	NA	8	1102.94	7.16	0.01
4	3.20	0.02	+	NA	NA	NA	NA	NA	NA	NA	NA	NA	7	1106.83	11.06	0.00
5	3.17	NA	NA	+	NA	NA	NA	NA	NA	NA	NA	NA	6	1107.15	11.38	0.00
12	3.19	0.02	+	NA	+	NA	NA	NA	NA	NA	NA	NA	8	1108.74	12.96	0.00
3	3.30	NA	+	NA	NA	NA	NA	NA	NA	NA	NA	NA	6	1109.37	13.60	0.00
2	3.13	0.02	NA	NA	NA	NA	NA	NA	NA	NA	NA	NA	6	1112.90	17.13	0.00
1	3.24	NA	NA	NA	NA	NA	NA	NA	NA	NA	NA	NA	5	1115.62	19.84	0.00
	I	AF	P	S	AF:P	AF:S	P:S	AF:P:S	NA	NA	NA	NA	df	AIC	delta	weight
40	3.26	+	+	+	NA	NA	+	NA	NA	NA	NA	NA	18	1086.32	0.00	0.48
8	3.23	+	+	+	NA	NA	NA	NA	NA	NA	NA	NA	17	1086.45	0.13	0.45
6	3.18	+	NA	+	NA	NA	NA	NA	NA	NA	NA	NA	16	1090.91	4.58	0.05
24	3.26	+	+	+	NA	+	NA	NA	NA	NA	NA	NA	27	1094.54	8.22	0.01
56	3.28	+	+	+	NA	+	+	NA	NA	NA	NA	NA	28	1094.76	8.44	0.01
22	3.21	+	NA	+	NA	+	NA	NA	NA	NA	NA	NA	26	1098.38	12.06	0.00
4	3.32	+	+	NA	NA	NA	NA	NA	NA	NA	NA	NA	16	1098.50	12.18	0.00
16	3.23	+	+	+	+	NA	NA	NA	NA	NA	NA	NA	27	1099.01	12.69	0.00
48	3.25	+	+	+	+	NA	+	NA	NA	NA	NA	NA	28	1099.05	12.72	0.00
7	3.24	NA	+	+	NA	NA	NA	NA	NA	NA	NA	NA	7	1101.18	14.86	0.00
39	3.26	NA	+	+	NA	NA	+	NA	NA	NA	NA	NA	8	1101.38	15.05	0.00
2	3.26	+	NA	NA	NA	NA	NA	NA	NA	NA	NA	NA	15	1103.44	17.12	0.00
32	3.26	+	+	+	+	+	NA	NA	NA	NA	NA	NA	37	1106.15	19.82	0.00
64	3.28	+	+	+	+	+	+	NA	NA	NA	NA	NA	38	1106.83	20.51	0.00
5	3.17	NA	NA	+	NA	NA	NA	NA	NA	NA	NA	NA	6	1107.15	20.83	0.00
3	3.30	NA	+	NA	NA	NA	NA	NA	NA	NA	NA	NA	6	1109.37	23.05	0.00
12	3.31	+	+	NA	+	NA	NA	NA	NA	NA	NA	NA	26	1112.25	25.93	0.00
1	3.24	NA	NA	NA	NA	NA	NA	NA	NA	NA	NA	NA	5	1115.62	29.29	0.00
128	3.26	+	+	+	+	+	+	+	NA	NA	NA	NA	46	1122.58	36.26	0.00
	I	AT	P	S	AT:P	AT:S	P:S	AT:P:S	NA	NA	NA	NA	df	AIC	delta	weight
40	1.92	0.14	+	+	NA	NA	+	NA	NA	NA	NA	NA	9	1088.48	0.00	0.21
8	1.90	0.14	+	+	NA	NA	NA	NA	NA	NA	NA	NA	8	1088.53	0.06	0.20
24	1.74	0.16	+	+	NA	+	NA	NA	NA	NA	NA	NA	9	1089.57	1.10	0.12
56	1.78	0.16	+	+	NA	+	+	NA	NA	NA	NA	NA	10	1089.80	1.33	0.11
16	1.73	0.16	+	+	+	NA	NA	NA	NA	NA	NA	NA	9	1090.12	1.65	0.09
48	1.79	0.16	+	+	+	NA	+	NA	NA	NA	NA	NA	10	1090.30	1.83	0.08
32	1.39	0.20	+	+	+	+	NA	NA	NA	NA	NA	NA	10	1090.49	2.02	0.08
64	1.50	0.19	+	+	+	+	+	NA	NA	NA	NA	NA	11	1091.19	2.71	0.05
128	1.53	0.19	+	+	+	+	+	+	NA	NA	NA	NA	12	1093.02	4.55	0.02
6	1.79	0.15	NA	+	NA	NA	NA	NA	NA	NA	NA	NA	7	1093.51	5.03	0.02
22	1.64	0.16	NA	+	NA	+	NA	NA	NA	NA	NA	NA	8	1094.78	6.30	0.01
4	2.15	0.12	+	NA	NA	NA	NA	NA	NA	NA	NA	NA	7	1100.12	11.65	0.00
7	3.24	NA	+	+	NA	NA	NA	NA	NA	NA	NA	NA	7	1101.18	12.71	0.00
12	1.91	0.15	+	NA	+	NA	NA	NA	NA	NA	NA	NA	8	1101.34	12.86	0.00
39	3.26	NA	+	+	NA	NA	+	NA	NA	NA	NA	NA	8	1101.38	12.90	0.00
2	2.03	0.13	NA	NA	NA	NA	NA	NA	NA	NA	NA	NA	6	1105.60	17.12	0.00
5	3.17	NA	NA	+	NA	NA	NA	NA	NA	NA	NA	NA	6	1107.15	18.68	0.00
3	3.30	NA	+	NA	NA	NA	NA	NA	NA	NA	NA	NA	6	1109.37	20.90	0.00
1	3.24	NA	NA	NA	NA	NA	NA	NA	NA	NA	NA	NA	5	1115.62	27.14	0.00
	I	AL	I(AL^2)	P	S	AL:P	AL:S	I(AL^2):P	I(AL^2):S	P:S	AL:P:S	I(AL^2):P:S	df	AIC	delta	weight
272	3.36	-0.08	0.01	+	+	NA	NA	NA	NA	+	NA	NA	10	1087.78	0.00	0.09
**16**	**3.35**	**-0.09**	**0.01**	**+**	**+**	**NA**	**NA**	**NA**	**NA**	**NA**	**NA**	**NA**	**9**	**1087.86**	**0.07**	**0.08**
144	3.31	-0.08	0.01	+	+	NA	NA	NA	+	NA	NA	NA	10	1088.70	0.92	0.06
400	3.33	-0.07	0.01	+	+	NA	NA	NA	+	+	NA	NA	11	1088.85	1.07	0.05
48	3.31	-0.08	0.01	+	+	NA	+	NA	NA	NA	NA	NA	10	1089.28	1.49	0.04
304	3.33	-0.07	0.01	+	+	NA	+	NA	NA	+	NA	NA	11	1089.35	1.56	0.04
176	3.38	-0.11	0.01	+	+	NA	+	NA	+	NA	NA	NA	11	1089.56	1.78	0.04
288	3.38	-0.09	0.01	+	+	+	NA	NA	NA	+	NA	NA	11	1089.66	1.88	0.03
432	3.40	-0.10	0.01	+	+	NA	+	NA	+	+	NA	NA	12	1089.81	2.02	0.03
336	3.37	-0.08	0.01	+	+	NA	NA	+	NA	+	NA	NA	11	1089.82	2.03	0.03
32	3.36	-0.09	0.01	+	+	+	NA	NA	NA	NA	NA	NA	10	1089.87	2.09	0.03
80	3.35	-0.09	0.01	+	+	NA	NA	+	NA	NA	NA	NA	10	1089.92	2.13	0.03
96	3.44	-0.12	0.01	+	+	+	NA	+	NA	NA	NA	NA	11	1090.66	2.88	0.02
352	3.45	-0.12	0.01	+	+	+	NA	+	NA	+	NA	NA	12	1090.68	2.89	0.02
208	3.31	-0.08	0.01	+	+	NA	NA	+	+	NA	NA	NA	11	1090.73	2.94	0.02
160	3.32	-0.08	0.01	+	+	+	NA	NA	+	NA	NA	NA	11	1090.76	2.98	0.02
416	3.34	-0.08	0.01	+	+	+	NA	NA	+	+	NA	NA	12	1090.83	3.04	0.02
464	3.33	-0.07	0.01	+	+	NA	NA	+	+	+	NA	NA	12	1090.93	3.14	0.02
224	3.40	-0.12	0.01	+	+	+	NA	+	+	NA	NA	NA	12	1091.14	3.35	0.02
399	3.14	NA	0.00	+	+	NA	NA	NA	+	+	NA	NA	10	1091.28	3.50	0.02
320	3.35	-0.08	0.01	+	+	+	+	NA	NA	+	NA	NA	12	1091.28	3.50	0.02
64	3.32	-0.08	0.01	+	+	+	+	NA	NA	NA	NA	NA	11	1091.33	3.54	0.01
112	3.31	-0.07	0.01	+	+	NA	+	+	NA	NA	NA	NA	11	1091.33	3.54	0.01
271	3.17	NA	0.00	+	+	NA	NA	NA	NA	+	NA	NA	9	1091.40	3.61	0.01
368	3.33	-0.07	0.01	+	+	NA	+	+	NA	+	NA	NA	12	1091.41	3.63	0.01
480	3.42	-0.11	0.01	+	+	+	NA	+	+	+	NA	NA	13	1091.50	3.71	0.01
240	3.38	-0.11	0.01	+	+	NA	+	+	+	NA	NA	NA	12	1091.51	3.73	0.01
256	3.49	-0.15	0.01	+	+	+	+	+	+	NA	NA	NA	13	1091.59	3.80	0.01
143	3.12	NA	0.00	+	+	NA	NA	NA	+	NA	NA	NA	9	1091.61	3.83	0.01
192	3.38	-0.11	0.01	+	+	+	+	NA	+	NA	NA	NA	12	1091.64	3.85	0.01
448	3.41	-0.10	0.01	+	+	+	+	NA	+	+	NA	NA	13	1091.85	4.06	0.01
496	3.39	-0.10	0.01	+	+	NA	+	+	+	+	NA	NA	13	1091.88	4.09	0.01
128	3.40	-0.11	0.01	+	+	+	+	+	NA	NA	NA	NA	12	1091.90	4.11	0.01
384	3.42	-0.11	0.01	+	+	+	+	+	NA	+	NA	NA	13	1092.12	4.34	0.01
512	3.50	-0.15	0.01	+	+	+	+	+	+	+	NA	NA	14	1092.15	4.36	0.01
15	3.14	NA	0.00	+	+	NA	NA	NA	NA	NA	NA	NA	8	1092.20	4.42	0.01
1488	3.33	-0.07	0.01	+	+	NA	NA	+	+	+	NA	+	13	1092.92	5.13	0.01
1504	3.43	-0.11	0.01	+	+	+	NA	+	+	+	NA	+	14	1093.17	5.38	0.01
1536	3.54	-0.16	0.01	+	+	+	+	+	+	+	NA	+	15	1093.25	5.46	0.01
12	3.29	-0.09	0.01	NA	+	NA	NA	NA	NA	NA	NA	NA	8	1093.32	5.54	0.01
832	3.35	-0.08	0.01	+	+	+	+	NA	NA	+	+	NA	13	1093.34	5.55	0.01
463	3.14	NA	0.00	+	+	NA	NA	+	+	+	NA	NA	11	1093.34	5.55	0.01
335	3.17	NA	0.00	+	+	NA	NA	+	NA	+	NA	NA	10	1093.46	5.67	0.01
207	3.11	NA	0.00	+	+	NA	NA	+	+	NA	NA	NA	10	1093.52	5.73	0.01
1024	3.54	-0.16	0.01	+	+	+	+	+	+	+	+	NA	15	1093.52	5.73	0.00
1520	3.40	-0.10	0.01	+	+	NA	+	+	+	+	NA	+	14	1093.66	5.88	0.00
960	3.42	-0.11	0.01	+	+	+	+	NA	+	+	+	NA	14	1093.76	5.98	0.00
896	3.43	-0.11	0.01	+	+	+	+	+	NA	+	+	NA	14	1093.99	6.20	0.00
79	3.14	NA	0.00	+	+	NA	NA	+	NA	NA	NA	NA	9	1094.22	6.43	0.00
140	3.26	-0.08	0.01	NA	+	NA	NA	NA	+	NA	NA	NA	9	1094.69	6.91	0.00
172	3.35	-0.12	0.01	NA	+	NA	+	NA	+	NA	NA	NA	10	1094.95	7.17	0.00
1487	3.15	NA	0.00	+	+	NA	NA	+	+	+	NA	+	12	1094.97	7.19	0.00
2048	3.51	-0.15	0.01	+	+	+	+	+	+	+	+	+	16	1095.10	7.31	0.00
44	3.27	-0.08	0.01	NA	+	NA	+	NA	NA	NA	NA	NA	9	1095.16	7.37	0.00
270	3.13	0.02	NA	+	+	NA	NA	NA	NA	+	NA	NA	9	1095.77	7.99	0.00
302	3.09	0.03	NA	+	+	NA	+	NA	NA	+	NA	NA	10	1095.92	8.13	0.00
46	3.06	0.03	NA	+	+	NA	+	NA	NA	NA	NA	NA	9	1096.28	8.49	0.00
14	3.11	0.02	NA	+	+	NA	NA	NA	NA	NA	NA	NA	8	1096.50	8.72	0.00
286	3.14	0.02	NA	+	+	+	NA	NA	NA	+	NA	NA	10	1097.76	9.97	0.00
11	3.07	NA	0.00	NA	+	NA	NA	NA	NA	NA	NA	NA	7	1097.85	10.06	0.00
318	3.09	0.03	NA	+	+	+	+	NA	NA	+	NA	NA	11	1097.96	10.17	0.00
139	3.05	NA	0.00	NA	+	NA	NA	NA	+	NA	NA	NA	8	1098.00	10.22	0.00
62	3.06	0.03	NA	+	+	+	+	NA	NA	NA	NA	NA	10	1098.34	10.55	0.00
30	3.11	0.02	NA	+	+	+	NA	NA	NA	NA	NA	NA	9	1098.56	10.77	0.00
830	3.11	0.03	NA	+	+	+	+	NA	NA	+	+	NA	12	1099.72	11.93	0.00
8	3.43	-0.08	0.01	+	NA	NA	NA	NA	NA	NA	NA	NA	8	1099.85	12.06	0.00
13	3.24	NA	NA	+	+	NA	NA	NA	NA	NA	NA	NA	7	1101.18	13.40	0.00
269	3.26	NA	NA	+	+	NA	NA	NA	NA	+	NA	NA	8	1101.38	13.59	0.00
72	3.42	-0.08	0.01	+	NA	NA	NA	+	NA	NA	NA	NA	9	1101.68	13.89	0.00
24	3.42	-0.08	0.01	+	NA	+	NA	NA	NA	NA	NA	NA	9	1101.84	14.06	0.00
10	3.04	0.02	NA	NA	+	NA	NA	NA	NA	NA	NA	NA	7	1102.32	14.53	0.00
88	3.49	-0.11	0.01	+	NA	+	NA	+	NA	NA	NA	NA	10	1102.90	15.11	0.00
42	3.00	0.03	NA	NA	+	NA	+	NA	NA	NA	NA	NA	8	1102.94	15.15	0.00
7	3.23	NA	0.00	+	NA	NA	NA	NA	NA	NA	NA	NA	7	1103.54	15.76	0.00
71	3.22	NA	0.00	+	NA	NA	NA	+	NA	NA	NA	NA	8	1105.25	17.46	0.00
4	3.37	-0.09	0.01	NA	NA	NA	NA	NA	NA	NA	NA	NA	7	1105.73	17.95	0.00
6	3.20	0.02	NA	+	NA	NA	NA	NA	NA	NA	NA	NA	7	1106.83	19.05	0.00
9	3.17	NA	NA	NA	+	NA	NA	NA	NA	NA	NA	NA	6	1107.15	19.37	0.00
22	3.19	0.02	NA	+	NA	+	NA	NA	NA	NA	NA	NA	8	1108.74	20.95	0.00
5	3.30	NA	NA	+	NA	NA	NA	NA	NA	NA	NA	NA	6	1109.37	21.59	0.00
3	3.16	NA	0.00	NA	NA	NA	NA	NA	NA	NA	NA	NA	6	1109.42	21.63	0.00
2	3.13	0.02	NA	NA	NA	NA	NA	NA	NA	NA	NA	NA	6	1112.90	25.12	0.00
1	3.24	NA	NA	NA	NA	NA	NA	NA	NA	NA	NA	NA	5	1115.62	27.83	0.00

ALPHA2-GLOBULIN

	I	AL	P	S	AL:P	AL:S	P:S	AL:P:S	NA	NA	NA	NA	df	logLik	AIC	delta	weight
5	5.88	NA	NA	+	NA	NA	NA	NA	NA	NA	NA	NA	6	-1129.41	2270.94	0.00	0.21
6	6.02	-0.03	NA	+	NA	NA	NA	NA	NA	NA	NA	NA	7	-1128.62	2271.42	0.48	0.16
22	5.95	-0.01	NA	+	NA	+	NA	NA	NA	NA	NA	NA	8	-1128.11	2272.45	1.50	0.10
1	5.79	NA	NA	NA	NA	NA	NA	NA	NA	NA	NA	NA	5	-1131.22	2272.53	1.59	0.09
7	5.85	NA	+	+	NA	NA	NA	NA	NA	NA	NA	NA	7	-1129.28	2272.73	1.78	0.09
8	6.00	-0.03	+	+	NA	NA	NA	NA	NA	NA	NA	NA	8	-1128.50	2273.23	2.29	0.07
2	5.89	-0.02	NA	NA	NA	NA	NA	NA	NA	NA	NA	NA	6	-1130.77	2273.67	2.72	0.05
3	5.76	NA	+	NA	NA	NA	NA	NA	NA	NA	NA	NA	6	-1131.05	2274.23	3.29	0.04
24	5.93	-0.01	+	+	NA	+	NA	NA	NA	NA	NA	NA	9	-1128.04	2274.36	3.42	0.04
39	5.84	NA	+	+	NA	NA	+	NA	NA	NA	NA	NA	8	-1129.26	2274.74	3.80	0.03
40	5.99	-0.03	+	+	NA	NA	+	NA	NA	NA	NA	NA	9	-1128.45	2275.17	4.23	0.03
16	5.99	-0.02	+	+	+	NA	NA	NA	NA	NA	NA	NA	9	-1128.50	2275.28	4.34	0.02
4	5.86	-0.02	+	NA	NA	NA	NA	NA	NA	NA	NA	NA	7	-1130.60	2275.38	4.43	0.02
56	5.91	-0.01	+	+	NA	+	+	NA	NA	NA	NA	NA	10	-1127.95	2276.24	5.30	0.01
32	5.92	-0.01	+	+	+	+	NA	NA	NA	NA	NA	NA	10	-1128.03	2276.41	5.46	0.01
48	5.97	-0.02	+	+	+	NA	+	NA	NA	NA	NA	NA	10	-1128.44	2277.22	6.28	0.01
12	5.88	-0.02	+	NA	+	NA	NA	NA	NA	NA	NA	NA	8	-1130.58	2277.38	6.44	0.01
64	5.89	-0.01	+	+	+	+	+	NA	NA	NA	NA	NA	11	-1127.93	2278.27	7.33	0.01
128	5.89	-0.01	+	+	+	+	+	+	NA	NA	NA	NA	12	-1127.92	2280.32	9.38	0.00
	I	AF	P	S	AF:P	AF:S	P:S	AF:P:S	NA	NA	NA	NA	df	logLik	AIC	delta	weight
5	5.88	NA	NA	+	NA	NA	NA	NA	NA	NA	NA	NA	6	-1129.41	2270.94	0.00	0.45
1	5.79	NA	NA	NA	NA	NA	NA	NA	NA	NA	NA	NA	5	-1131.22	2272.53	1.59	0.20
7	5.85	NA	+	+	NA	NA	NA	NA	NA	NA	NA	NA	7	-1129.28	2272.73	1.78	0.19
3	5.76	NA	+	NA	NA	NA	NA	NA	NA	NA	NA	NA	6	-1131.05	2274.23	3.29	0.09
39	5.84	NA	+	+	NA	NA	+	NA	NA	NA	NA	NA	8	-1129.26	2274.74	3.80	0.07
6	6.03	+	NA	+	NA	NA	NA	NA	NA	NA	NA	NA	16	-1125.17	2283.21	12.26	0.00
8	6.00	+	+	+	NA	NA	NA	NA	NA	NA	NA	NA	17	-1125.02	2285.00	14.06	0.00
2	5.92	+	NA	NA	NA	NA	NA	NA	NA	NA	NA	NA	15	-1127.35	2285.45	14.51	0.00
40	5.99	+	+	+	NA	NA	+	NA	NA	NA	NA	NA	18	-1124.94	2286.95	16.01	0.00
4	5.89	+	+	NA	NA	NA	NA	NA	NA	NA	NA	NA	16	-1127.14	2287.15	16.20	0.00
22	6.11	+	NA	+	NA	+	NA	NA	NA	NA	NA	NA	26	-1119.70	2293.66	22.72	0.00
24	6.09	+	+	+	NA	+	NA	NA	NA	NA	NA	NA	27	-1119.59	2295.61	24.67	0.00
56	6.06	+	+	+	NA	+	+	NA	NA	NA	NA	NA	28	-1119.38	2297.37	26.43	0.00
16	6.01	+	+	+	+	NA	NA	NA	NA	NA	NA	NA	27	-1120.94	2298.32	27.37	0.00
48	6.00	+	+	+	+	NA	+	NA	NA	NA	NA	NA	28	-1120.87	2300.35	29.41	0.00
12	5.91	+	+	NA	+	NA	NA	NA	NA	NA	NA	NA	26	-1123.07	2300.40	29.45	0.00
32	6.08	+	+	+	+	+	NA	NA	NA	NA	NA	NA	37	-1116.03	2310.66	39.71	0.00
64	6.05	+	+	+	+	+	+	NA	NA	NA	NA	NA	38	-1115.81	2312.45	41.51	0.00
128	6.08	+	+	+	+	+	+	+	NA	NA	NA	NA	46	-1113.44	2326.05	55.10	0.00
	I	AT	P	S	AT:P	AT:S	P:S	AT:P:S	NA	NA	NA	NA	df	logLik	AIC	delta	weight
5	5.88	NA	NA	+	NA	NA	NA	NA	NA	NA	NA	NA	6	-1129.41	2270.94	0.00	0.25
6	9.22	-0.30	NA	+	NA	NA	NA	NA	NA	NA	NA	NA	7	-1128.93	2272.04	1.09	0.15
1	5.79	NA	NA	NA	NA	NA	NA	NA	NA	NA	NA	NA	5	-1131.22	2272.53	1.59	0.11
7	5.85	NA	+	+	NA	NA	NA	NA	NA	NA	NA	NA	7	-1129.28	2272.73	1.78	0.10
8	9.12	-0.30	+	+	NA	NA	NA	NA	NA	NA	NA	NA	8	-1128.82	2273.87	2.93	0.06
2	8.46	-0.24	NA	NA	NA	NA	NA	NA	NA	NA	NA	NA	6	-1130.91	2273.95	3.01	0.06
22	8.89	-0.27	NA	+	NA	+	NA	NA	NA	NA	NA	NA	8	-1128.91	2274.03	3.09	0.05
3	5.76	NA	+	NA	NA	NA	NA	NA	NA	NA	NA	NA	6	-1131.05	2274.23	3.29	0.05
39	5.84	NA	+	+	NA	NA	+	NA	NA	NA	NA	NA	8	-1129.26	2274.74	3.80	0.04
4	8.36	-0.24	+	NA	NA	NA	NA	NA	NA	NA	NA	NA	7	-1130.76	2275.70	4.75	0.02
16	9.81	-0.36	+	+	+	NA	NA	NA	NA	NA	NA	NA	9	-1128.78	2275.85	4.91	0.02
24	8.81	-0.27	+	+	NA	+	NA	NA	NA	NA	NA	NA	9	-1128.80	2275.88	4.94	0.02
40	9.10	-0.29	+	+	NA	NA	+	NA	NA	NA	NA	NA	9	-1128.81	2275.90	4.96	0.02
12	9.18	-0.31	+	NA	+	NA	NA	NA	NA	NA	NA	NA	8	-1130.70	2277.63	6.69	0.01
32	9.49	-0.33	+	+	+	+	NA	NA	NA	NA	NA	NA	10	-1128.77	2277.89	6.95	0.01
48	9.76	-0.35	+	+	+	NA	+	NA	NA	NA	NA	NA	10	-1128.77	2277.89	6.95	0.01
56	8.77	-0.26	+	+	NA	+	+	NA	NA	NA	NA	NA	10	-1128.78	2277.91	6.96	0.01
64	9.40	-0.32	+	+	+	+	+	NA	NA	NA	NA	NA	11	-1128.76	2279.93	8.99	0.00
128	9.40	-0.32	+	+	+	+	+	+	NA	NA	NA	NA	11	-1128.76	2279.93	8.99	0.00
	I	AL	I(AL^2)	P	S	AL:P	AL:S	I(AL^2):P	I(AL^2):S	P:S	AL:P:S	I(AL^2):P:S	df	logLik	AIC	delta	weight
9	5.88	NA	NA	NA	+	NA	NA	NA	NA	NA	NA	NA	6	-1129.41	2270.94	0.00	0.10
10	6.02	-0.03	NA	NA	+	NA	NA	NA	NA	NA	NA	NA	7	-1128.62	2271.42	0.48	0.08
11	5.94	NA	0.00	NA	+	NA	NA	NA	NA	NA	NA	NA	7	-1128.91	2271.99	1.05	0.06
139	5.89	NA	0.00	NA	+	NA	NA	NA	+	NA	NA	NA	8	-1128.01	2272.24	1.30	0.05
42	5.95	-0.01	NA	NA	+	NA	+	NA	NA	NA	NA	NA	8	-1128.11	2272.45	1.50	0.05
1	5.79	NA	NA	NA	NA	NA	NA	NA	NA	NA	NA	NA	5	-1131.22	2272.53	1.59	0.05
12	6.20	-0.10	0.01	NA	+	NA	NA	NA	NA	NA	NA	NA	8	-1128.20	2272.62	1.67	0.04
13	5.85	NA	NA	+	+	NA	NA	NA	NA	NA	NA	NA	7	-1129.28	2272.73	1.78	0.04
14	6.00	-0.03	NA	+	+	NA	NA	NA	NA	NA	NA	NA	8	-1128.50	2273.23	2.29	0.03
140	6.11	-0.08	0.01	NA	+	NA	NA	NA	+	NA	NA	NA	9	-1127.55	2273.39	2.45	0.03
2	5.89	-0.02	NA	NA	NA	NA	NA	NA	NA	NA	NA	NA	6	-1130.77	2273.67	2.72	0.03
15	5.92	NA	0.00	+	+	NA	NA	NA	NA	NA	NA	NA	8	-1128.79	2273.80	2.86	0.02
44	6.10	-0.08	0.01	NA	+	NA	+	NA	NA	NA	NA	NA	9	-1127.84	2273.96	3.02	0.02
3	5.83	NA	0.00	NA	NA	NA	NA	NA	NA	NA	NA	NA	6	-1130.98	2274.10	3.16	0.02
143	5.88	NA	0.00	+	+	NA	NA	NA	+	NA	NA	NA	9	-1127.94	2274.17	3.23	0.02
5	5.76	NA	NA	+	NA	NA	NA	NA	NA	NA	NA	NA	6	-1131.05	2274.23	3.29	0.02
172	6.29	-0.15	0.01	NA	+	NA	+	NA	+	NA	NA	NA	10	-1126.96	2274.26	3.32	0.02
46	5.93	-0.01	NA	+	+	NA	+	NA	NA	NA	NA	NA	9	-1128.04	2274.36	3.42	0.02
16	6.18	-0.10	0.01	+	+	NA	NA	NA	NA	NA	NA	NA	9	-1128.06	2274.40	3.45	0.02
4	6.10	-0.11	0.01	NA	NA	NA	NA	NA	NA	NA	NA	NA	7	-1130.23	2274.63	3.68	0.02
269	5.84	NA	NA	+	+	NA	NA	NA	NA	+	NA	NA	8	-1129.26	2274.74	3.80	0.02
270	5.99	-0.03	NA	+	+	NA	NA	NA	NA	+	NA	NA	9	-1128.45	2275.17	4.23	0.01
30	5.99	-0.02	NA	+	+	+	NA	NA	NA	NA	NA	NA	9	-1128.50	2275.28	4.34	0.01
144	6.10	-0.09	0.01	+	+	NA	NA	NA	+	NA	NA	NA	10	-1127.47	2275.29	4.34	0.01
6	5.86	-0.02	NA	+	NA	NA	NA	NA	NA	NA	NA	NA	7	-1130.60	2275.38	4.43	0.01
271	5.91	NA	0.00	+	+	NA	NA	NA	NA	+	NA	NA	9	-1128.75	2275.78	4.83	0.01
7	5.80	NA	0.00	+	NA	NA	NA	NA	NA	NA	NA	NA	7	-1130.82	2275.81	4.87	0.01
48	6.09	-0.08	0.01	+	+	NA	+	NA	NA	NA	NA	NA	10	-1127.75	2275.85	4.90	0.01
79	5.92	NA	0.00	+	+	NA	NA	+	NA	NA	NA	NA	9	-1128.79	2275.86	4.91	0.01
399	5.86	NA	0.00	+	+	NA	NA	NA	+	+	NA	NA	10	-1127.86	2276.06	5.12	0.01
176	6.28	-0.16	0.01	+	+	NA	+	NA	+	NA	NA	NA	11	-1126.83	2276.07	5.13	0.01
207	5.87	NA	0.00	+	+	NA	NA	+	+	NA	NA	NA	10	-1127.93	2276.20	5.26	0.01
302	5.91	-0.01	NA	+	+	NA	+	NA	NA	+	NA	NA	10	-1127.95	2276.24	5.30	0.01
272	6.17	-0.11	0.01	+	+	NA	NA	NA	NA	+	NA	NA	10	-1127.97	2276.29	5.35	0.01
8	6.07	-0.11	0.01	+	NA	NA	NA	NA	NA	NA	NA	NA	8	-1130.04	2276.30	5.36	0.01
62	5.92	-0.01	NA	+	+	+	+	NA	NA	NA	NA	NA	10	-1128.03	2276.41	5.46	0.01
80	6.18	-0.10	0.01	+	+	NA	NA	+	NA	NA	NA	NA	10	-1128.06	2276.45	5.51	0.01
32	6.18	-0.10	0.01	+	+	+	NA	NA	NA	NA	NA	NA	10	-1128.06	2276.46	5.51	0.01
400	6.09	-0.09	0.01	+	+	NA	NA	NA	+	+	NA	NA	11	-1127.34	2277.10	6.16	0.00
286	5.97	-0.02	NA	+	+	+	NA	NA	NA	+	NA	NA	10	-1128.44	2277.22	6.28	0.00
160	6.09	-0.08	0.01	+	+	+	NA	NA	+	NA	NA	NA	11	-1127.47	2277.35	6.40	0.00
208	6.09	-0.09	0.01	+	+	NA	NA	+	+	NA	NA	NA	11	-1127.47	2277.35	6.41	0.00
22	5.88	-0.02	NA	+	NA	+	NA	NA	NA	NA	NA	NA	8	-1130.58	2277.38	6.44	0.00
304	6.08	-0.08	0.01	+	+	NA	+	NA	NA	+	NA	NA	11	-1127.64	2277.69	6.75	0.00
71	5.81	NA	0.00	+	NA	NA	NA	+	NA	NA	NA	NA	8	-1130.80	2277.82	6.88	0.00
335	5.90	NA	0.00	+	+	NA	NA	+	NA	+	NA	NA	10	-1128.75	2277.83	6.89	0.00
432	6.27	-0.16	0.01	+	+	NA	+	NA	+	+	NA	NA	12	-1126.68	2277.86	6.92	0.00
64	6.09	-0.08	0.01	+	+	+	+	NA	NA	NA	NA	NA	11	-1127.75	2277.91	6.97	0.00
112	6.09	-0.08	0.01	+	+	NA	+	+	NA	NA	NA	NA	11	-1127.75	2277.92	6.97	0.00
463	5.85	NA	0.00	+	+	NA	NA	+	+	+	NA	NA	11	-1127.82	2278.05	7.11	0.00
240	6.27	-0.16	0.01	+	+	NA	+	+	+	NA	NA	NA	12	-1126.81	2278.10	7.16	0.00
192	6.26	-0.15	0.01	+	+	+	+	NA	+	NA	NA	NA	12	-1126.81	2278.10	7.16	0.00
72	6.09	-0.11	0.01	+	NA	NA	NA	+	NA	NA	NA	NA	9	-1129.99	2278.27	7.32	0.00
318	5.89	-0.01	NA	+	+	+	+	NA	NA	+	NA	NA	11	-1127.93	2278.27	7.33	0.00
24	6.10	-0.11	0.01	+	NA	+	NA	NA	NA	NA	NA	NA	9	-1130.00	2278.28	7.34	0.00
288	6.17	-0.11	0.01	+	+	+	NA	NA	NA	+	NA	NA	11	-1127.97	2278.35	7.41	0.00
336	6.17	-0.11	0.01	+	+	NA	NA	+	NA	+	NA	NA	11	-1127.97	2278.36	7.41	0.00
96	6.14	-0.09	0.01	+	+	+	NA	+	NA	NA	NA	NA	11	-1128.03	2278.48	7.54	0.00
416	6.06	-0.09	0.01	+	+	+	NA	NA	+	+	NA	NA	12	-1127.32	2279.14	8.19	0.00
464	6.07	-0.09	0.01	+	+	NA	NA	+	+	+	NA	NA	12	-1127.33	2279.14	8.20	0.00
224	6.07	-0.08	0.01	+	+	+	NA	+	+	NA	NA	NA	12	-1127.46	2279.42	8.47	0.00
320	6.06	-0.08	0.01	+	+	+	+	NA	NA	+	NA	NA	12	-1127.63	2279.75	8.81	0.00
368	6.07	-0.08	0.01	+	+	NA	+	+	NA	+	NA	NA	12	-1127.63	2279.76	8.81	0.00
496	6.25	-0.16	0.01	+	+	NA	+	+	+	+	NA	NA	13	-1126.63	2279.84	8.89	0.00
448	6.24	-0.16	0.01	+	+	+	+	NA	+	+	NA	NA	13	-1126.64	2279.85	8.90	0.00
128	6.06	-0.07	0.00	+	+	+	+	+	NA	NA	NA	NA	12	-1127.74	2279.97	9.03	0.00
1487	5.85	NA	0.00	+	+	NA	NA	+	+	+	NA	+	12	-1127.82	2280.12	9.18	0.00
256	6.27	-0.16	0.01	+	+	+	+	+	+	NA	NA	NA	13	-1126.81	2280.18	9.24	0.00
830	5.89	-0.01	NA	+	+	+	+	NA	NA	+	+	NA	12	-1127.92	2280.32	9.38	0.00
88	6.08	-0.10	0.01	+	NA	+	NA	+	NA	NA	NA	NA	10	-1129.99	2280.33	9.38	0.00
352	6.13	-0.09	0.01	+	+	+	NA	+	NA	+	NA	NA	12	-1127.95	2280.40	9.45	0.00
1488	6.07	-0.09	0.01	+	+	NA	NA	+	+	+	NA	+	13	-1127.32	2281.21	10.27	0.00
480	6.06	-0.08	0.01	+	+	+	NA	+	+	+	NA	NA	13	-1127.32	2281.22	10.28	0.00
832	6.06	-0.08	0.01	+	+	+	+	NA	NA	+	+	NA	13	-1127.60	2281.77	10.82	0.00
384	6.05	-0.07	0.00	+	+	+	+	+	NA	+	NA	NA	13	-1127.63	2281.82	10.88	0.00
1520	6.26	-0.16	0.01	+	+	NA	+	+	+	+	NA	+	14	-1126.62	2281.89	10.95	0.00
512	6.26	-0.17	0.01	+	+	+	+	+	+	+	NA	NA	14	-1126.63	2281.92	10.98	0.00
960	6.24	-0.16	0.01	+	+	+	+	NA	+	+	+	NA	14	-1126.64	2281.93	10.99	0.00
1504	6.06	-0.08	0.01	+	+	+	NA	+	+	+	NA	+	14	-1127.32	2283.29	12.35	0.00
896	6.03	-0.07	0.00	+	+	+	+	+	NA	+	+	NA	14	-1127.58	2283.83	12.89	0.00
1536	6.28	-0.17	0.01	+	+	+	+	+	+	+	NA	+	15	-1126.61	2283.98	13.03	0.00
1024	6.27	-0.17	0.01	+	+	+	+	+	+	+	+	NA	15	-1126.63	2284.01	13.07	0.00
2048	6.20	-0.14	0.01	+	+	+	+	+	+	+	+	+	16	-1126.45	2285.76	14.82	0.00

BETA-GLOBULIN

	I	AL	P	S	AL:P	AL:S	P:S	AL:P:S	NA	NA	NA	NA	df	AIC	delta	weight
8	5.29	0.24	+	+	NA	NA	NA	NA	NA	NA	NA	NA	8	2648.81	0.00	0.23
40	5.36	0.24	+	+	NA	NA	+	NA	NA	NA	NA	NA	9	2648.91	0.10	0.22
16	5.16	0.26	+	+	+	NA	NA	NA	NA	NA	NA	NA	9	2649.70	0.89	0.15
48	5.25	0.26	+	+	+	NA	+	NA	NA	NA	NA	NA	10	2650.20	1.38	0.12
24	5.30	0.23	+	+	NA	+	NA	NA	NA	NA	NA	NA	9	2650.84	2.03	0.08
56	5.38	0.23	+	+	NA	+	+	NA	NA	NA	NA	NA	10	2650.90	2.08	0.08
32	5.17	0.26	+	+	+	+	NA	NA	NA	NA	NA	NA	10	2651.76	2.95	0.05
64	5.27	0.26	+	+	+	+	+	NA	NA	NA	NA	NA	11	2652.22	3.41	0.04
128	5.32	0.25	+	+	+	+	+	+	NA	NA	NA	NA	12	2654.02	5.21	0.02
12	5.44	0.26	+	NA	+	NA	NA	NA	NA	NA	NA	NA	8	2660.20	11.38	0.00
4	5.63	0.22	+	NA	NA	NA	NA	NA	NA	NA	NA	NA	7	2660.42	11.60	0.00
6	5.58	0.24	NA	+	NA	NA	NA	NA	NA	NA	NA	NA	7	2660.50	11.68	0.00
22	5.55	0.24	NA	+	NA	+	NA	NA	NA	NA	NA	NA	8	2662.46	13.64	0.00
2	5.90	0.22	NA	NA	NA	NA	NA	NA	NA	NA	NA	NA	6	2670.60	21.79	0.00
7	6.99	NA	+	+	NA	NA	NA	NA	NA	NA	NA	NA	7	2675.44	26.63	0.00
39	7.07	NA	+	+	NA	NA	+	NA	NA	NA	NA	NA	8	2675.99	27.18	0.00
3	7.21	NA	+	NA	NA	NA	NA	NA	NA	NA	NA	NA	6	2684.56	35.75	0.00
5	7.28	NA	NA	+	NA	NA	NA	NA	NA	NA	NA	NA	6	2688.70	39.89	0.00
1	7.49	NA	NA	NA	NA	NA	NA	NA	NA	NA	NA	NA	5	2697.02	48.21	0.00
	I	AT	P	S	AT:P	AT:S	P:S	AT:P:S	NA	NA	NA	NA	df	AIC	delta	weight
24	5.88	+	+	+	NA	+	NA	NA	NA	NA	NA	NA	27	2656.37	0.00	0.29
56	5.95	+	+	+	NA	+	+	NA	NA	NA	NA	NA	28	2656.57	0.20	0.27
8	5.72	+	+	+	NA	NA	NA	NA	NA	NA	NA	NA	17	2657.10	0.73	0.20
40	5.79	+	+	+	NA	NA	+	NA	NA	NA	NA	NA	18	2657.62	1.25	0.16
16	5.85	+	+	+	+	NA	NA	NA	NA	NA	NA	NA	27	2660.50	4.13	0.04
48	5.90	+	+	+	+	NA	+	NA	NA	NA	NA	NA	28	2661.74	5.38	0.02
32	5.98	+	+	+	+	+	NA	NA	NA	NA	NA	NA	37	2662.87	6.50	0.01
64	6.05	+	+	+	+	+	+	NA	NA	NA	NA	NA	38	2663.95	7.58	0.01
4	6.02	+	+	NA	NA	NA	NA	NA	NA	NA	NA	NA	16	2668.87	12.50	0.00
6	6.02	+	NA	+	NA	NA	NA	NA	NA	NA	NA	NA	16	2669.39	13.02	0.00
22	6.15	+	NA	+	NA	+	NA	NA	NA	NA	NA	NA	26	2670.18	13.81	0.00
12	6.10	+	+	NA	+	NA	NA	NA	NA	NA	NA	NA	26	2671.91	15.54	0.00
128	5.99	+	+	+	+	+	+	+	NA	NA	NA	NA	46	2673.92	17.55	0.00
7	6.99	NA	+	+	NA	NA	NA	NA	NA	NA	NA	NA	7	2675.44	19.07	0.00
39	7.07	NA	+	+	NA	NA	+	NA	NA	NA	NA	NA	8	2675.99	19.62	0.00
2	6.29	+	NA	NA	NA	NA	NA	NA	NA	NA	NA	NA	15	2679.52	23.16	0.00
3	7.21	NA	+	NA	NA	NA	NA	NA	NA	NA	NA	NA	6	2684.56	28.19	0.00
5	7.28	NA	NA	+	NA	NA	NA	NA	NA	NA	NA	NA	6	2688.70	32.33	0.00
1	7.49	NA	NA	NA	NA	NA	NA	NA	NA	NA	NA	NA	5	2697.02	40.66	0.00
	I	AT	P	S	AT:P	AT:S	P:S	AT:P:S	NA	NA	NA	NA	df	AIC	delta	weight
8	4.85	0.29	+	+	NA	NA	NA	NA	NA	NA	NA	NA	8	2651.52	0.00	0.21
16	4.62	0.33	+	+	+	NA	NA	NA	NA	NA	NA	NA	9	2651.53	0.01	0.21
40	4.91	0.29	+	+	NA	NA	+	NA	NA	NA	NA	NA	9	2652.06	0.54	0.16
48	4.69	0.33	+	+	+	NA	+	NA	NA	NA	NA	NA	10	2652.54	1.03	0.13
24	4.88	0.28	+	+	NA	+	NA	NA	NA	NA	NA	NA	9	2653.53	2.01	0.08
32	4.63	0.33	+	+	+	+	NA	NA	NA	NA	NA	NA	10	2653.59	2.07	0.08
56	4.95	0.28	+	+	NA	+	+	NA	NA	NA	NA	NA	10	2654.01	2.49	0.06
64	4.72	0.32	+	+	+	+	+	NA	NA	NA	NA	NA	11	2654.59	3.07	0.05
128	4.84	0.30	+	+	+	+	+	+	NA	NA	NA	NA	12	2655.67	4.15	0.03
12	4.91	0.32	+	NA	+	NA	NA	NA	NA	NA	NA	NA	8	2662.07	10.55	0.00
6	5.14	0.29	NA	+	NA	NA	NA	NA	NA	NA	NA	NA	7	2663.21	11.69	0.00
4	5.22	0.27	+	NA	NA	NA	NA	NA	NA	NA	NA	NA	7	2663.33	11.81	0.00
22	5.11	0.29	NA	+	NA	+	NA	NA	NA	NA	NA	NA	8	2665.21	13.69	0.00
2	5.50	0.27	NA	NA	NA	NA	NA	NA	NA	NA	NA	NA	6	2673.52	22.01	0.00
7	6.99	NA	+	+	NA	NA	NA	NA	NA	NA	NA	NA	7	2675.44	23.92	0.00
39	7.07	NA	+	+	NA	NA	+	NA	NA	NA	NA	NA	8	2675.99	24.47	0.00
3	7.21	NA	+	NA	NA	NA	NA	NA	NA	NA	NA	NA	6	2684.56	33.04	0.00
5	7.28	NA	NA	+	NA	NA	NA	NA	NA	NA	NA	NA	6	2688.70	37.18	0.00
1	7.49	NA	NA	NA	NA	NA	NA	NA	NA	NA	NA	NA	5	2697.02	45.50	0.00
	I	AL	I(AL^2)	P	S	AL:P	AL:S	I(AL^2):P	I(AL^2):S	P:S	AL:P:S	I(AL^2):P:S	df	AIC	delta	weight
96	6.00	-0.10	0.03	+	+	+	NA	+	NA	NA	NA	NA	11	2647.08	0.00	0.09
79	5.76	NA	0.02	+	+	NA	NA	+	NA	NA	NA	NA	9	2647.86	0.79	0.06
15	5.84	NA	0.02	+	+	NA	NA	NA	NA	NA	NA	NA	8	2647.90	0.82	0.06
352	6.04	-0.09	0.03	+	+	+	NA	+	NA	+	NA	NA	12	2648.09	1.01	0.05
271	5.91	NA	0.02	+	+	NA	NA	NA	NA	+	NA	NA	9	2648.49	1.42	0.04
14	5.29	0.24	NA	+	+	NA	NA	NA	NA	NA	NA	NA	8	2648.81	1.74	0.04
270	5.36	0.24	NA	+	+	NA	NA	NA	NA	+	NA	NA	9	2648.91	1.84	0.03
335	5.82	NA	0.02	+	+	NA	NA	+	NA	+	NA	NA	10	2648.95	1.87	0.03
80	5.49	0.11	0.01	+	+	NA	NA	+	NA	NA	NA	NA	10	2649.04	1.97	0.03
128	6.02	-0.10	0.03	+	+	+	+	+	NA	NA	NA	NA	12	2649.14	2.06	0.03
224	6.00	-0.10	0.03	+	+	+	NA	+	+	NA	NA	NA	12	2649.15	2.08	0.03
16	5.60	0.10	0.01	+	+	NA	NA	NA	NA	NA	NA	NA	9	2649.24	2.17	0.03
143	5.87	NA	0.02	+	+	NA	NA	NA	+	NA	NA	NA	9	2649.57	2.49	0.02
272	5.64	0.11	0.01	+	+	NA	NA	NA	NA	+	NA	NA	10	2649.61	2.53	0.02
30	5.16	0.26	NA	+	+	+	NA	NA	NA	NA	NA	NA	9	2649.70	2.63	0.02
207	5.78	NA	0.02	+	+	NA	NA	+	+	NA	NA	NA	10	2649.74	2.66	0.02
336	5.54	0.12	0.01	+	+	NA	NA	+	NA	+	NA	NA	11	2649.94	2.86	0.02
399	5.95	NA	0.02	+	+	NA	NA	NA	+	+	NA	NA	10	2650.00	2.92	0.02
384	6.07	-0.09	0.03	+	+	+	+	+	NA	+	NA	NA	13	2650.13	3.05	0.02
480	6.05	-0.09	0.03	+	+	+	NA	+	+	+	NA	NA	13	2650.16	3.08	0.02
286	5.25	0.26	NA	+	+	+	NA	NA	NA	+	NA	NA	10	2650.20	3.12	0.02
32	5.47	0.13	0.01	+	+	+	NA	NA	NA	NA	NA	NA	10	2650.32	3.24	0.02
1504	6.12	-0.10	0.03	+	+	+	NA	+	+	+	NA	+	14	2650.62	3.55	0.01
463	5.86	NA	0.02	+	+	NA	NA	+	+	+	NA	NA	11	2650.70	3.62	0.01
46	5.30	0.23	NA	+	+	NA	+	NA	NA	NA	NA	NA	9	2650.84	3.77	0.01
302	5.38	0.23	NA	+	+	NA	+	NA	NA	+	NA	NA	10	2650.90	3.82	0.01
288	5.53	0.14	0.01	+	+	+	NA	NA	NA	+	NA	NA	11	2651.01	3.94	0.01
112	5.54	0.09	0.01	+	+	NA	+	+	NA	NA	NA	NA	11	2651.03	3.96	0.01
208	5.52	0.10	0.01	+	+	NA	NA	+	+	NA	NA	NA	11	2651.05	3.98	0.01
256	6.10	-0.14	0.03	+	+	+	+	+	+	NA	NA	NA	13	2651.07	3.99	0.01
144	5.65	0.08	0.01	+	+	NA	NA	NA	+	NA	NA	NA	10	2651.09	4.02	0.01
48	5.67	0.08	0.01	+	+	NA	+	NA	NA	NA	NA	NA	10	2651.11	4.03	0.01
896	6.17	-0.12	0.03	+	+	+	+	+	NA	+	+	NA	14	2651.31	4.23	0.01
400	5.70	0.10	0.01	+	+	NA	NA	NA	+	+	NA	NA	11	2651.35	4.28	0.01
304	5.73	0.09	0.01	+	+	NA	+	NA	NA	+	NA	NA	11	2651.39	4.31	0.01
62	5.17	0.26	NA	+	+	+	+	NA	NA	NA	NA	NA	10	2651.76	4.68	0.01
368	5.60	0.10	0.01	+	+	NA	+	+	NA	+	NA	NA	12	2651.87	4.79	0.01
464	5.58	0.11	0.01	+	+	NA	NA	+	+	+	NA	NA	12	2651.88	4.81	0.01
512	6.13	-0.12	0.03	+	+	+	+	+	+	+	NA	NA	14	2652.14	5.06	0.01
318	5.27	0.26	NA	+	+	+	+	NA	NA	+	NA	NA	11	2652.22	5.14	0.01
1536	6.29	-0.17	0.03	+	+	+	+	+	+	+	NA	+	15	2652.25	5.17	0.01
64	5.53	0.11	0.01	+	+	+	+	NA	NA	NA	NA	NA	11	2652.26	5.18	0.01
160	5.51	0.12	0.01	+	+	+	NA	NA	+	NA	NA	NA	11	2652.27	5.19	0.01
1487	5.88	NA	0.02	+	+	NA	NA	+	+	+	NA	+	12	2652.48	5.40	0.01
2048	6.06	-0.07	0.03	+	+	+	+	+	+	+	+	+	16	2652.65	5.58	0.01
416	5.59	0.12	0.01	+	+	+	NA	NA	+	+	NA	NA	12	2652.87	5.79	0.00
320	5.61	0.11	0.01	+	+	+	+	NA	NA	+	NA	NA	12	2652.88	5.80	0.00
240	5.56	0.08	0.01	+	+	NA	+	+	+	NA	NA	NA	12	2653.09	6.02	0.00
1024	6.29	-0.17	0.03	+	+	+	+	+	+	+	+	NA	15	2653.12	6.04	0.00
176	5.65	0.08	0.01	+	+	NA	+	NA	+	NA	NA	NA	11	2653.16	6.09	0.00
432	5.69	0.10	0.01	+	+	NA	+	NA	+	+	NA	NA	12	2653.43	6.35	0.00
1488	5.58	0.12	0.01	+	+	NA	NA	+	+	+	NA	+	13	2653.45	6.37	0.00
496	5.60	0.10	0.01	+	+	NA	+	+	+	+	NA	NA	13	2653.95	6.88	0.00
830	5.32	0.25	NA	+	+	+	+	NA	NA	+	+	NA	12	2654.02	6.95	0.00
192	5.53	0.11	0.01	+	+	+	+	NA	+	NA	NA	NA	12	2654.34	7.26	0.00
832	5.63	0.11	0.01	+	+	+	+	NA	NA	+	+	NA	13	2654.82	7.75	0.00
448	5.59	0.12	0.01	+	+	+	+	NA	+	+	NA	NA	13	2654.95	7.88	0.00
1520	5.63	0.10	0.01	+	+	NA	+	+	+	+	NA	+	14	2655.46	8.38	0.00
960	5.62	0.12	0.01	+	+	+	+	NA	+	+	+	NA	14	2656.91	9.83	0.00
71	6.03	NA	0.02	+	NA	NA	NA	+	NA	NA	NA	NA	8	2658.73	11.65	0.00
88	6.20	-0.07	0.03	+	NA	+	NA	+	NA	NA	NA	NA	10	2658.73	11.66	0.00
11	6.14	NA	0.02	NA	+	NA	NA	NA	NA	NA	NA	NA	7	2659.73	12.65	0.00
72	5.74	0.12	0.01	+	NA	NA	NA	+	NA	NA	NA	NA	9	2659.75	12.67	0.00
7	6.16	NA	0.02	+	NA	NA	NA	NA	NA	NA	NA	NA	7	2660.09	13.01	0.00
22	5.44	0.26	NA	+	NA	+	NA	NA	NA	NA	NA	NA	8	2660.20	13.12	0.00
6	5.63	0.22	NA	+	NA	NA	NA	NA	NA	NA	NA	NA	7	2660.42	13.34	0.00
10	5.58	0.24	NA	NA	+	NA	NA	NA	NA	NA	NA	NA	7	2660.50	13.42	0.00
12	5.89	0.10	0.01	NA	+	NA	NA	NA	NA	NA	NA	NA	8	2661.02	13.94	0.00
24	5.69	0.15	0.01	+	NA	+	NA	NA	NA	NA	NA	NA	9	2661.30	14.22	0.00
8	5.90	0.10	0.01	+	NA	NA	NA	NA	NA	NA	NA	NA	8	2661.33	14.25	0.00
139	6.15	NA	0.02	NA	+	NA	NA	NA	+	NA	NA	NA	8	2661.75	14.67	0.00
42	5.55	0.24	NA	NA	+	NA	+	NA	NA	NA	NA	NA	8	2662.46	15.38	0.00
44	5.88	0.10	0.01	NA	+	NA	+	NA	NA	NA	NA	NA	9	2663.08	16.00	0.00
140	5.89	0.10	0.01	NA	+	NA	NA	NA	+	NA	NA	NA	9	2663.08	16.00	0.00
172	5.85	0.12	0.01	NA	+	NA	+	NA	+	NA	NA	NA	10	2665.10	18.02	0.00
3	6.44	NA	0.02	NA	NA	NA	NA	NA	NA	NA	NA	NA	6	2670.41	23.33	0.00
2	5.90	0.22	NA	NA	NA	NA	NA	NA	NA	NA	NA	NA	6	2670.60	23.53	0.00
4	6.17	0.11	0.01	NA	NA	NA	NA	NA	NA	NA	NA	NA	7	2671.58	24.50	0.00
13	6.99	NA	NA	+	+	NA	NA	NA	NA	NA	NA	NA	7	2675.44	28.36	0.00
269	7.07	NA	NA	+	+	NA	NA	NA	NA	+	NA	NA	8	2675.99	28.91	0.00
5	7.21	NA	NA	+	NA	NA	NA	NA	NA	NA	NA	NA	6	2684.56	37.48	0.00
9	7.28	NA	NA	NA	+	NA	NA	NA	NA	NA	NA	NA	6	2688.70	41.62	0.00
1	7.49	NA	NA	NA	NA	NA	NA	NA	NA	NA	NA	NA	5	2697.02	49.95	0.00

GAMMA-GLOBULIN

	I	AL	P	S	AL:P	AL:S	P:S	AL:P:S	NA	NA	NA	NA	df	AIC	delta	weight
4	13.21	0.41	+	NA	NA	NA	NA	NA	NA	NA	NA	NA	7	3642.15	0.00	0.32
12	12.87	0.48	+	NA	+	NA	NA	NA	NA	NA	NA	NA	8	3642.70	0.55	0.24
8	13.19	0.41	+	+	NA	NA	NA	NA	NA	NA	NA	NA	8	3644.19	2.04	0.11
16	12.88	0.48	+	+	+	NA	NA	NA	NA	NA	NA	NA	9	3644.75	2.60	0.09
24	12.99	0.45	+	+	NA	+	NA	NA	NA	NA	NA	NA	9	3645.44	3.30	0.06
32	12.64	0.53	+	+	+	+	NA	NA	NA	NA	NA	NA	10	3645.83	3.69	0.05
40	13.24	0.42	+	+	NA	NA	+	NA	NA	NA	NA	NA	9	3646.05	3.90	0.05
48	12.92	0.48	+	+	+	NA	+	NA	NA	NA	NA	NA	10	3646.73	4.59	0.03
56	13.04	0.46	+	+	NA	+	+	NA	NA	NA	NA	NA	10	3647.36	5.21	0.02
64	12.67	0.53	+	+	+	+	+	NA	NA	NA	NA	NA	11	3647.86	5.71	0.02
128	12.74	0.51	+	+	+	+	+	+	NA	NA	NA	NA	12	3649.82	7.67	0.01
3	15.91	NA	+	NA	NA	NA	NA	NA	NA	NA	NA	NA	6	3669.97	27.83	0.00
7	15.92	NA	+	+	NA	NA	NA	NA	NA	NA	NA	NA	7	3672.01	29.87	0.00
39	15.97	NA	+	+	NA	NA	+	NA	NA	NA	NA	NA	8	3673.99	31.84	0.00
2	15.36	0.40	NA	NA	NA	NA	NA	NA	NA	NA	NA	NA	6	3747.84	105.70	0.00
22	14.87	0.49	NA	+	NA	+	NA	NA	NA	NA	NA	NA	8	3748.65	106.50	0.00
6	15.36	0.40	NA	+	NA	NA	NA	NA	NA	NA	NA	NA	7	3749.88	107.74	0.00
1	17.72	NA	NA	NA	NA	NA	NA	NA	NA	NA	NA	NA	5	3767.53	125.38	0.00
5	17.73	NA	NA	+	NA	NA	NA	NA	NA	NA	NA	NA	6	3769.56	127.42	0.00
	**I**	**AF**	**P**	**S**	**AF:P**	**AF:S**	**P:S**	**AF:P:S**	**NA**	**NA**	**NA**	**NA**	**df**	**AIC**	**delta**	**weight**
4	14.23	+	+	NA	NA	NA	NA	NA	NA	NA	NA	NA	16	3654.50	0.00	0.67
8	14.21	+	+	+	NA	NA	NA	NA	NA	NA	NA	NA	17	3656.59	2.09	0.24
40	14.25	+	+	+	NA	NA	+	NA	NA	NA	NA	NA	18	3658.61	4.12	0.09
24	14.32	+	+	+	NA	+	NA	NA	NA	NA	NA	NA	27	3666.03	11.53	0.00
12	14.20	+	+	NA	+	NA	NA	NA	NA	NA	NA	NA	26	3666.07	11.57	0.00
56	14.36	+	+	+	NA	+	+	NA	NA	NA	NA	NA	28	3668.12	13.62	0.00
16	14.20	+	+	+	+	NA	NA	NA	NA	NA	NA	NA	27	3668.24	13.75	0.00
3	15.91	NA	+	NA	NA	NA	NA	NA	NA	NA	NA	NA	6	3669.97	15.48	0.00
48	14.21	+	+	+	+	NA	+	NA	NA	NA	NA	NA	28	3670.43	15.93	0.00
7	15.92	NA	+	+	NA	NA	NA	NA	NA	NA	NA	NA	7	3672.01	17.52	0.00
39	15.97	NA	+	+	NA	NA	+	NA	NA	NA	NA	NA	8	3673.99	19.49	0.00
32	14.27	+	+	+	+	+	NA	NA	NA	NA	NA	NA	37	3679.84	25.35	0.00
64	14.26	+	+	+	+	+	+	NA	NA	NA	NA	NA	38	3682.10	27.60	0.00
128	14.37	+	+	+	+	+	+	+	NA	NA	NA	NA	46	3697.22	42.73	0.00
2	16.28	+	NA	NA	NA	NA	NA	NA	NA	NA	NA	NA	15	3761.97	107.48	0.00
6	16.28	+	NA	+	NA	NA	NA	NA	NA	NA	NA	NA	16	3764.08	109.58	0.00
1	17.72	NA	NA	NA	NA	NA	NA	NA	NA	NA	NA	NA	5	3767.53	113.03	0.00
5	17.73	NA	NA	+	NA	NA	NA	NA	NA	NA	NA	NA	6	3769.56	115.07	0.00
22	16.19	+	NA	+	NA	+	NA	NA	NA	NA	NA	NA	26	3772.10	117.60	0.00
	**I**	**AT**	**P**	**S**	**AT:P**	**AT:S**	**P:S**	**AT:P:S**	**NA**	**NA**	**NA**	**NA**	**df**	**AIC**	**delta**	**weight**
4	12.32	0.52	+	NA	NA	NA	NA	NA	NA	NA	NA	NA	7	3640.45	0.00	0.31
12	11.81	0.61	+	NA	+	NA	NA	NA	NA	NA	NA	NA	8	3640.73	0.28	0.27
8	12.28	0.52	+	+	NA	NA	NA	NA	NA	NA	NA	NA	8	3642.47	2.02	0.11
16	11.81	0.61	+	+	+	NA	NA	NA	NA	NA	NA	NA	9	3642.78	2.34	0.10
24	12.10	0.55	+	+	NA	+	NA	NA	NA	NA	NA	NA	9	3644.16	3.71	0.05
32	11.53	0.66	+	+	+	+	NA	NA	NA	NA	NA	NA	10	3644.24	3.79	0.05
40	12.32	0.52	+	+	NA	NA	+	NA	NA	NA	NA	NA	9	3644.40	3.95	0.04
48	11.83	0.61	+	+	+	NA	+	NA	NA	NA	NA	NA	10	3644.82	4.37	0.03
56	12.14	0.56	+	+	NA	+	+	NA	NA	NA	NA	NA	10	3646.12	5.67	0.02
64	11.55	0.66	+	+	+	+	+	NA	NA	NA	NA	NA	11	3646.30	5.85	0.02
128	11.64	0.64	+	+	+	+	+	+	NA	NA	NA	NA	12	3648.28	7.83	0.01
3	15.91	NA	+	NA	NA	NA	NA	NA	NA	NA	NA	NA	6	3669.97	29.52	0.00
7	15.92	NA	+	+	NA	NA	NA	NA	NA	NA	NA	NA	7	3672.01	31.56	0.00
39	15.97	NA	+	+	NA	NA	+	NA	NA	NA	NA	NA	8	3673.99	33.54	0.00
2	14.53	0.50	NA	NA	NA	NA	NA	NA	NA	NA	NA	NA	6	3747.01	106.56	0.00
22	14.00	0.58	NA	+	NA	+	NA	NA	NA	NA	NA	NA	8	3748.90	108.45	0.00
6	14.51	0.50	NA	+	NA	NA	NA	NA	NA	NA	NA	NA	7	3749.05	108.60	0.00
1	17.72	NA	NA	NA	NA	NA	NA	NA	NA	NA	NA	NA	5	3767.53	127.08	0.00
5	17.73	NA	NA	+	NA	NA	NA	NA	NA	NA	NA	NA	6	3769.56	129.11	0.00
	**I**	**AL**	**I(AL^2)**	**P**	**S**	**AL:P**	**AL:S**	**I(AL^2):P**	**I(AL^2):S**	**P:S**	**AL:P:S**	**I(AL^2):P:S**	**df**	**AIC**	**delta**	**weight**
71	13.94	NA	0.04	+	NA	NA	NA	+	NA	NA	NA	NA	8	3641.37	0.00	0.12
7	14.17	NA	0.03	+	NA	NA	NA	NA	NA	NA	NA	NA	7	3641.62	0.25	0.10
6	13.21	0.41	NA	+	NA	NA	NA	NA	NA	NA	NA	NA	7	3642.15	0.77	0.08
22	12.87	0.48	NA	+	NA	+	NA	NA	NA	NA	NA	NA	8	3642.70	1.32	0.06
72	13.50	0.18	0.03	+	NA	NA	NA	+	NA	NA	NA	NA	9	3642.86	1.49	0.06
8	13.74	0.17	0.02	+	NA	NA	NA	NA	NA	NA	NA	NA	8	3643.15	1.78	0.05
79	13.94	NA	0.04	+	+	NA	NA	+	NA	NA	NA	NA	9	3643.43	2.06	0.04
88	14.14	-0.08	0.05	+	NA	+	NA	+	NA	NA	NA	NA	10	3643.57	2.20	0.04
15	14.13	NA	0.03	+	+	NA	NA	NA	NA	NA	NA	NA	8	3643.64	2.27	0.04
24	13.39	0.25	0.02	+	NA	+	NA	NA	NA	NA	NA	NA	9	3643.76	2.39	0.04
14	13.19	0.41	NA	+	+	NA	NA	NA	NA	NA	NA	NA	8	3644.19	2.82	0.03
30	12.88	0.48	NA	+	+	+	NA	NA	NA	NA	NA	NA	9	3644.75	3.38	0.02
80	13.50	0.18	0.03	+	+	NA	NA	+	NA	NA	NA	NA	10	3644.92	3.55	0.02
207	13.86	NA	0.04	+	+	NA	NA	+	+	NA	NA	NA	10	3645.03	3.66	0.02
16	13.71	0.17	0.02	+	+	NA	NA	NA	NA	NA	NA	NA	9	3645.18	3.81	0.02
46	12.99	0.45	NA	+	+	NA	+	NA	NA	NA	NA	NA	9	3645.44	4.07	0.02
335	13.96	NA	0.04	+	+	NA	NA	+	NA	+	NA	NA	10	3645.48	4.11	0.01
143	14.08	NA	0.03	+	+	NA	NA	NA	+	NA	NA	NA	9	3645.49	4.12	0.01
271	14.18	NA	0.03	+	+	NA	NA	NA	NA	+	NA	NA	9	3645.58	4.21	0.01
96	14.13	-0.08	0.05	+	+	+	NA	+	NA	NA	NA	NA	11	3645.64	4.27	0.01
32	13.39	0.25	0.02	+	+	+	NA	NA	NA	NA	NA	NA	10	3645.83	4.46	0.01
62	12.64	0.53	NA	+	+	+	+	NA	NA	NA	NA	NA	10	3645.83	4.46	0.01
270	13.24	0.42	NA	+	+	NA	NA	NA	NA	+	NA	NA	9	3646.05	4.68	0.01
112	13.19	0.27	0.02	+	+	NA	+	+	NA	NA	NA	NA	11	3646.24	4.87	0.01
208	13.28	0.23	0.03	+	+	NA	NA	+	+	NA	NA	NA	11	3646.26	4.89	0.01
224	13.94	-0.05	0.05	+	+	+	NA	+	+	NA	NA	NA	12	3646.70	5.32	0.01
286	12.92	0.48	NA	+	+	+	NA	NA	NA	+	NA	NA	10	3646.73	5.36	0.01
48	13.48	0.24	0.02	+	+	NA	+	NA	NA	NA	NA	NA	10	3646.77	5.40	0.01
128	13.83	0.00	0.04	+	+	+	+	+	NA	NA	NA	NA	12	3646.77	5.40	0.01
144	13.57	0.20	0.02	+	+	NA	NA	NA	+	NA	NA	NA	10	3646.87	5.50	0.01
336	13.52	0.19	0.03	+	+	NA	NA	+	NA	+	NA	NA	11	3646.96	5.59	0.01
272	13.75	0.18	0.02	+	+	NA	NA	NA	NA	+	NA	NA	10	3647.10	5.72	0.01
463	13.86	NA	0.04	+	+	NA	NA	+	+	+	NA	NA	11	3647.10	5.73	0.01
64	13.10	0.33	0.02	+	+	+	+	NA	NA	NA	NA	NA	11	3647.26	5.89	0.01
160	13.19	0.29	0.02	+	+	+	NA	NA	+	NA	NA	NA	11	3647.32	5.95	0.01
302	13.04	0.46	NA	+	+	NA	+	NA	NA	+	NA	NA	10	3647.36	5.99	0.01
399	14.13	NA	0.03	+	+	NA	NA	NA	+	+	NA	NA	10	3647.46	6.09	0.01
352	14.15	-0.07	0.05	+	+	+	NA	+	NA	+	NA	NA	12	3647.70	6.33	0.00
288	13.42	0.25	0.02	+	+	+	NA	NA	NA	+	NA	NA	11	3647.84	6.47	0.00
318	12.67	0.53	NA	+	+	+	+	NA	NA	+	NA	NA	11	3647.86	6.49	0.00
368	13.21	0.27	0.02	+	+	NA	+	+	NA	+	NA	NA	12	3648.31	6.94	0.00
240	13.22	0.25	0.02	+	+	NA	+	+	+	NA	NA	NA	12	3648.31	6.94	0.00
464	13.29	0.23	0.03	+	+	NA	NA	+	+	+	NA	NA	12	3648.32	6.95	0.00
304	13.52	0.24	0.02	+	+	NA	+	NA	NA	+	NA	NA	11	3648.72	7.34	0.00
480	13.95	-0.05	0.05	+	+	+	NA	+	+	+	NA	NA	13	3648.78	7.41	0.00
256	13.95	-0.06	0.05	+	+	+	+	+	+	NA	NA	NA	13	3648.78	7.41	0.00
176	13.40	0.27	0.01	+	+	NA	+	NA	+	NA	NA	NA	11	3648.80	7.43	0.00
400	13.61	0.21	0.02	+	+	NA	NA	NA	+	+	NA	NA	11	3648.82	7.44	0.00
384	13.84	0.00	0.04	+	+	+	+	+	NA	+	NA	NA	13	3648.85	7.48	0.00
1487	13.88	NA	0.04	+	+	NA	NA	+	+	+	NA	+	12	3649.15	7.78	0.00
320	13.13	0.33	0.02	+	+	+	+	NA	NA	+	NA	NA	12	3649.31	7.94	0.00
192	13.09	0.33	0.02	+	+	+	+	NA	+	NA	NA	NA	12	3649.34	7.97	0.00
416	13.22	0.29	0.02	+	+	+	NA	NA	+	+	NA	NA	12	3649.37	8.00	0.00
830	12.74	0.51	NA	+	+	+	+	NA	NA	+	+	NA	12	3649.82	8.45	0.00
1488	13.29	0.24	0.02	+	+	NA	NA	+	+	+	NA	+	13	3650.30	8.93	0.00
496	13.23	0.26	0.02	+	+	NA	+	+	+	+	NA	NA	13	3650.39	9.01	0.00
1504	14.03	-0.06	0.05	+	+	+	NA	+	+	+	NA	+	14	3650.40	9.03	0.00
896	13.99	-0.04	0.04	+	+	+	+	+	NA	+	+	NA	14	3650.53	9.16	0.00
432	13.44	0.28	0.01	+	+	NA	+	NA	+	+	NA	NA	12	3650.74	9.37	0.00
512	13.95	-0.06	0.05	+	+	+	+	+	+	+	NA	NA	14	3650.87	9.49	0.00
832	13.16	0.33	0.01	+	+	+	+	NA	NA	+	+	NA	13	3651.34	9.96	0.00
448	13.12	0.33	0.01	+	+	+	+	NA	+	+	NA	NA	13	3651.39	10.02	0.00
1520	13.25	0.26	0.02	+	+	NA	+	+	+	+	NA	+	14	3652.38	11.01	0.00
1024	14.19	-0.13	0.05	+	+	+	+	+	+	+	+	NA	15	3652.45	11.08	0.00
1536	14.12	-0.10	0.05	+	+	+	+	+	+	+	NA	+	15	3652.46	11.09	0.00
960	13.16	0.33	0.01	+	+	+	+	NA	+	+	+	NA	14	3653.42	12.05	0.00
2048	14.17	-0.12	0.05	+	+	+	+	+	+	+	+	+	16	3654.55	13.18	0.00
5	15.91	NA	NA	+	NA	NA	NA	NA	NA	NA	NA	NA	6	3669.97	28.60	0.00
13	15.92	NA	NA	+	+	NA	NA	NA	NA	NA	NA	NA	7	3672.01	30.64	0.00
269	15.97	NA	NA	+	+	NA	NA	NA	NA	+	NA	NA	8	3673.99	32.62	0.00
2	15.36	0.40	NA	NA	NA	NA	NA	NA	NA	NA	NA	NA	6	3747.84	106.47	0.00
3	16.31	NA	0.03	NA	NA	NA	NA	NA	NA	NA	NA	NA	6	3748.52	107.14	0.00
42	14.87	0.49	NA	NA	+	NA	+	NA	NA	NA	NA	NA	8	3748.65	107.27	0.00
4	15.68	0.26	0.01	NA	NA	NA	NA	NA	NA	NA	NA	NA	7	3749.58	108.21	0.00
10	15.36	0.40	NA	NA	+	NA	NA	NA	NA	NA	NA	NA	7	3749.88	108.51	0.00
11	16.29	NA	0.03	NA	+	NA	NA	NA	NA	NA	NA	NA	7	3750.55	109.18	0.00
44	14.98	0.44	0.00	NA	+	NA	+	NA	NA	NA	NA	NA	9	3750.67	109.30	0.00
139	16.12	NA	0.04	NA	+	NA	NA	NA	+	NA	NA	NA	8	3750.87	109.50	0.00
140	15.24	0.34	0.01	NA	+	NA	NA	NA	+	NA	NA	NA	9	3751.25	109.87	0.00
12	15.66	0.26	0.01	NA	+	NA	NA	NA	NA	NA	NA	NA	8	3751.62	110.25	0.00
172	14.81	0.51	0.00	NA	+	NA	+	NA	+	NA	NA	NA	10	3752.55	111.18	0.00
1	17.72	NA	NA	NA	NA	NA	NA	NA	NA	NA	NA	NA	5	3767.53	126.16	0.00
9	17.73	NA	NA	NA	+	NA	NA	NA	NA	NA	NA	NA	6	3769.56	128.19	0.00

HAPTOGLOBIN

	I	AL	P	S	AL:P	AL:S	P:S	AL:P:S	NA	NA	NA	NA	df	AIC	delta	weight
40	-0.04	0.06	+	+	NA	NA	+	NA	NA	NA	NA	NA	9	1852.38	0.00	0.32
56	-0.07	0.07	+	+	NA	+	+	NA	NA	NA	NA	NA	10	1854.08	1.70	0.14
48	-0.09	0.07	+	+	+	NA	+	NA	NA	NA	NA	NA	10	1854.09	1.71	0.14
8	-0.09	0.06	+	+	NA	NA	NA	NA	NA	NA	NA	NA	8	1854.65	2.27	0.10
16	-0.15	0.07	+	+	+	NA	NA	NA	NA	NA	NA	NA	9	1855.75	3.37	0.06
64	-0.12	0.08	+	+	+	+	+	NA	NA	NA	NA	NA	11	1855.78	3.40	0.06
24	-0.13	0.06	+	+	NA	+	NA	NA	NA	NA	NA	NA	9	1856.23	3.85	0.05
6	-0.01	0.06	NA	+	NA	NA	NA	NA	NA	NA	NA	NA	7	1856.69	4.31	0.04
32	-0.19	0.08	+	+	+	+	NA	NA	NA	NA	NA	NA	10	1857.23	4.85	0.03
128	-0.13	0.08	+	+	+	+	+	+	NA	NA	NA	NA	12	1857.78	5.40	0.02
22	-0.07	0.07	NA	+	NA	+	NA	NA	NA	NA	NA	NA	8	1857.83	5.45	0.02
39	0.40	NA	+	+	NA	NA	+	NA	NA	NA	NA	NA	8	1858.67	6.29	0.01
7	0.33	NA	+	+	NA	NA	NA	NA	NA	NA	NA	NA	7	1860.38	8.00	0.01
4	0.05	0.05	+	NA	NA	NA	NA	NA	NA	NA	NA	NA	7	1861.40	9.02	0.00
12	-0.04	0.07	+	NA	+	NA	NA	NA	NA	NA	NA	NA	8	1861.79	9.41	0.00
2	0.12	0.05	NA	NA	NA	NA	NA	NA	NA	NA	NA	NA	6	1862.90	10.52	0.00
5	0.42	NA	NA	+	NA	NA	NA	NA	NA	NA	NA	NA	6	1863.43	11.06	0.00
3	0.43	NA	+	NA	NA	NA	NA	NA	NA	NA	NA	NA	6	1865.99	13.61	0.00
1	0.50	NA	NA	NA	NA	NA	NA	NA	NA	NA	NA	NA	5	1868.50	16.12	0.00
	I	AF	P	S	AF:P	AF:S	P:S	AF:P:S	NA	NA	NA	NA	df	AIC	delta	weight
56	0.21	+	+	+	NA	+	+	NA	NA	NA	NA	NA	28	1850.99	0.00	0.35
24	0.17	+	+	+	NA	+	NA	NA	NA	NA	NA	NA	27	1851.54	0.55	0.26
40	0.10	+	+	+	NA	NA	+	NA	NA	NA	NA	NA	18	1852.32	1.33	0.18
8	0.05	+	+	+	NA	NA	NA	NA	NA	NA	NA	NA	17	1853.45	2.47	0.10
22	0.24	+	NA	+	NA	+	NA	NA	NA	NA	NA	NA	26	1854.50	3.52	0.06
6	0.13	+	NA	+	NA	NA	NA	NA	NA	NA	NA	NA	16	1856.34	5.35	0.02
39	0.40	NA	+	+	NA	NA	+	NA	NA	NA	NA	NA	8	1858.67	7.68	0.01
7	0.33	NA	+	+	NA	NA	NA	NA	NA	NA	NA	NA	7	1860.38	9.39	0.00
32	0.20	+	+	+	+	+	NA	NA	NA	NA	NA	NA	37	1860.61	9.62	0.00
4	0.17	+	+	NA	NA	NA	NA	NA	NA	NA	NA	NA	16	1861.19	10.20	0.00
64	0.24	+	+	+	+	+	+	NA	NA	NA	NA	NA	38	1861.23	10.25	0.00
48	0.14	+	+	+	+	NA	+	NA	NA	NA	NA	NA	28	1862.23	11.24	0.00
16	0.10	+	+	+	+	NA	NA	NA	NA	NA	NA	NA	27	1862.56	11.58	0.00
2	0.25	+	NA	NA	NA	NA	NA	NA	NA	NA	NA	NA	15	1863.40	12.41	0.00
5	0.42	NA	NA	+	NA	NA	NA	NA	NA	NA	NA	NA	6	1863.43	12.45	0.00
3	0.43	NA	+	NA	NA	NA	NA	NA	NA	NA	NA	NA	6	1865.99	15.01	0.00
128	0.20	+	+	+	+	+	+	+	NA	NA	NA	NA	46	1867.13	16.15	0.00
1	0.50	NA	NA	NA	NA	NA	NA	NA	NA	NA	NA	NA	5	1868.50	17.51	0.00
12	0.21	+	+	NA	+	NA	NA	NA	NA	NA	NA	NA	26	1870.49	19.51	0.00
	I	AT	P	S	AT:P	AT:S	P:S	AT:P:S	NA	NA	NA	NA	df	AIC	delta	weight
16	-4.24	0.48	+	+	+	NA	NA	NA	NA	NA	NA	NA	9	1835.31	0.00	0.30
48	-4.11	0.47	+	+	+	NA	+	NA	NA	NA	NA	NA	10	1835.74	0.43	0.24
32	-4.55	0.52	+	+	+	+	NA	NA	NA	NA	NA	NA	10	1836.87	1.57	0.14
64	-4.36	0.50	+	+	+	+	+	NA	NA	NA	NA	NA	11	1837.52	2.22	0.10
128	-4.23	0.49	+	+	+	+	+	+	NA	NA	NA	NA	12	1838.48	3.18	0.06
40	-3.10	0.36	+	+	NA	NA	+	NA	NA	NA	NA	NA	9	1838.73	3.43	0.05
8	-3.14	0.36	+	+	NA	NA	NA	NA	NA	NA	NA	NA	8	1839.35	4.05	0.04
56	-3.03	0.36	+	+	NA	+	+	NA	NA	NA	NA	NA	10	1840.73	5.42	0.02
24	-3.11	0.36	+	+	NA	+	NA	NA	NA	NA	NA	NA	9	1841.40	6.10	0.01
12	-4.00	0.47	+	NA	+	NA	NA	NA	NA	NA	NA	NA	8	1842.42	7.12	0.01
6	-3.00	0.36	NA	+	NA	NA	NA	NA	NA	NA	NA	NA	7	1842.59	7.28	0.01
22	-3.02	0.36	NA	+	NA	+	NA	NA	NA	NA	NA	NA	8	1844.64	9.33	0.00
4	-2.79	0.34	+	NA	NA	NA	NA	NA	NA	NA	NA	NA	7	1847.40	12.10	0.00
2	-2.67	0.33	NA	NA	NA	NA	NA	NA	NA	NA	NA	NA	6	1849.85	14.54	0.00
39	0.40	NA	+	+	NA	NA	+	NA	NA	NA	NA	NA	8	1858.67	23.36	0.00
7	0.33	NA	+	+	NA	NA	NA	NA	NA	NA	NA	NA	7	1860.38	25.07	0.00
5	0.42	NA	NA	+	NA	NA	NA	NA	NA	NA	NA	NA	6	1863.43	28.13	0.00
3	0.43	NA	+	NA	NA	NA	NA	NA	NA	NA	NA	NA	6	1865.99	30.69	0.00
1	0.50	NA	NA	NA	NA	NA	NA	NA	NA	NA	NA	NA	5	1868.50	33.19	0.00
	I	AL	I(AL^2)	P	S	AL:P	AL:S	I(AL^2):P	I(AL^2):S	P:S	AL:P:S	I(AL^2):P:S	df	AIC	delta	weight
352	0.61	-0.24	0.02	+	+	+	NA	+	NA	+	NA	NA	12	1843.60	0.00	0.12
96	0.59	-0.25	0.03	+	+	+	NA	+	NA	NA	NA	NA	11	1843.94	0.34	0.10
272	0.37	-0.12	0.01	+	+	NA	NA	NA	NA	+	NA	NA	10	1845.24	1.65	0.05
480	0.59	-0.23	0.02	+	+	+	NA	+	+	+	NA	NA	13	1845.40	1.80	0.05
224	0.56	-0.24	0.03	+	+	+	NA	+	+	NA	NA	NA	12	1845.55	1.95	0.05
384	0.60	-0.23	0.02	+	+	+	+	+	NA	+	NA	NA	13	1845.63	2.03	0.04
128	0.56	-0.24	0.03	+	+	+	+	+	NA	NA	NA	NA	12	1845.89	2.30	0.04
256	0.72	-0.31	0.03	+	+	+	+	+	+	NA	NA	NA	13	1845.91	2.32	0.04
512	0.74	-0.30	0.03	+	+	+	+	+	+	+	NA	NA	14	1846.01	2.41	0.04
336	0.32	-0.12	0.02	+	+	NA	NA	+	NA	+	NA	NA	11	1846.39	2.80	0.03
16	0.34	-0.13	0.02	+	+	NA	NA	NA	NA	NA	NA	NA	9	1846.45	2.85	0.03
80	0.29	-0.13	0.02	+	+	NA	NA	+	NA	NA	NA	NA	10	1846.91	3.31	0.02
271	0.07	NA	0.01	+	+	NA	NA	NA	NA	+	NA	NA	9	1846.99	3.40	0.02
288	0.33	-0.11	0.01	+	+	+	NA	NA	NA	+	NA	NA	11	1847.13	3.53	0.02
400	0.36	-0.12	0.01	+	+	NA	NA	NA	+	+	NA	NA	11	1847.30	3.70	0.02
304	0.37	-0.12	0.01	+	+	NA	+	NA	NA	+	NA	NA	11	1847.30	3.71	0.02

1504	0.59	-0.23	0.02	+	+	+	NA	+	+	+	NA	+	14	1847.46	3.86	0.02
896	0.60	-0.23	0.02	+	+	+	+	+	NA	+	+	NA	14	1847.72	4.12	0.02
1536	0.77	-0.30	0.03	+	+	+	+	+	+	+	NA	+	15	1847.85	4.25	0.01
335	0.04	NA	0.01	+	+	NA	NA	+	NA	+	NA	NA	10	1847.91	4.31	0.01
32	0.29	-0.12	0.02	+	+	+	NA	NA	NA	NA	NA	NA	10	1847.99	4.39	0.01
1024	0.77	-0.30	0.03	+	+	+	+	+	+	+	+	NA	15	1848.01	4.41	0.01
464	0.30	-0.11	0.02	+	+	NA	NA	+	+	+	NA	NA	12	1848.39	4.80	0.01
144	0.33	-0.13	0.02	+	+	NA	NA	NA	+	NA	NA	NA	10	1848.46	4.86	0.01
368	0.32	-0.12	0.02	+	+	NA	+	+	NA	+	NA	NA	12	1848.47	4.87	0.01
48	0.34	-0.13	0.02	+	+	NA	+	NA	NA	NA	NA	NA	10	1848.51	4.91	0.01
432	0.46	-0.16	0.02	+	+	NA	+	NA	+	+	NA	NA	12	1848.60	5.01	0.01
399	0.06	NA	0.01	+	+	NA	NA	NA	+	+	NA	NA	10	1848.79	5.19	0.01
208	0.27	-0.12	0.02	+	+	NA	NA	+	+	NA	NA	NA	11	1848.80	5.20	0.01
12	0.42	-0.13	0.02	NA	+	NA	NA	NA	NA	NA	NA	NA	8	1848.87	5.27	0.01
112	0.28	-0.12	0.02	+	+	NA	+	+	NA	NA	NA	NA	11	1848.95	5.36	0.01
79	-0.03	NA	0.01	+	+	NA	NA	+	NA	NA	NA	NA	9	1849.10	5.50	0.01
15	0.02	NA	0.01	+	+	NA	NA	NA	NA	NA	NA	NA	8	1849.12	5.52	0.01
416	0.32	-0.11	0.01	+	+	+	NA	NA	+	+	NA	NA	12	1849.17	5.58	0.01
320	0.34	-0.12	0.01	+	+	+	+	NA	NA	+	NA	NA	12	1849.20	5.61	0.01
2048	0.69	-0.27	0.03	+	+	+	+	+	+	+	+	+	16	1849.31	5.72	0.01
496	0.42	-0.16	0.02	+	+	NA	+	+	+	+	NA	NA	13	1849.48	5.88	0.01
463	0.02	NA	0.01	+	+	NA	NA	+	+	+	NA	NA	11	1849.55	5.95	0.01
176	0.44	-0.17	0.02	+	+	NA	+	NA	+	NA	NA	NA	11	1849.70	6.10	0.01
240	0.39	-0.17	0.02	+	+	NA	+	+	+	NA	NA	NA	12	1849.72	6.12	0.01
160	0.27	-0.12	0.02	+	+	+	NA	NA	+	NA	NA	NA	11	1849.96	6.36	0.01
64	0.29	-0.12	0.02	+	+	+	+	NA	NA	NA	NA	NA	11	1850.05	6.45	0.00
1488	0.31	-0.12	0.02	+	+	NA	NA	+	+	+	NA	+	13	1850.28	6.69	0.00
448	0.42	-0.15	0.02	+	+	+	+	NA	+	+	NA	NA	13	1850.41	6.81	0.00
207	-0.05	NA	0.01	+	+	NA	NA	+	+	NA	NA	NA	10	1850.44	6.84	0.00
140	0.39	-0.12	0.02	NA	+	NA	NA	NA	+	NA	NA	NA	9	1850.69	7.09	0.00
143	0.00	NA	0.01	+	+	NA	NA	NA	+	NA	NA	NA	9	1850.75	7.15	0.00
44	0.39	-0.12	0.02	NA	+	NA	+	NA	NA	NA	NA	NA	9	1850.84	7.24	0.00
832	0.32	-0.11	0.02	+	+	+	+	NA	NA	+	+	NA	13	1850.87	7.27	0.00
192	0.38	-0.16	0.02	+	+	+	+	NA	+	NA	NA	NA	12	1851.06	7.46	0.00
11	0.10	NA	0.01	NA	+	NA	NA	NA	NA	NA	NA	NA	7	1851.29	7.69	0.00
1520	0.41	-0.16	0.02	+	+	NA	+	+	+	+	NA	+	14	1851.52	7.92	0.00
1487	0.01	NA	0.01	+	+	NA	NA	+	+	+	NA	+	12	1851.61	8.01	0.00
88	0.66	-0.23	0.02	+	NA	+	NA	+	NA	NA	NA	NA	10	1852.19	8.59	0.00
172	0.48	-0.16	0.02	NA	+	NA	+	NA	+	NA	NA	NA	10	1852.19	8.60	0.00
960	0.40	-0.15	0.02	+	+	+	+	NA	+	+	+	NA	14	1852.27	8.67	0.00
270	-0.04	0.06	NA	+	+	NA	NA	NA	NA	+	NA	NA	9	1852.38	8.78	0.00
139	0.07	NA	0.01	NA	+	NA	NA	NA	+	NA	NA	NA	8	1852.53	8.94	0.00
72	0.39	-0.12	0.02	+	NA	NA	NA	+	NA	NA	NA	NA	9	1854.05	10.46	0.00
302	-0.07	0.07	NA	+	+	NA	+	NA	NA	+	NA	NA	10	1854.08	10.48	0.00
286	-0.09	0.07	NA	+	+	+	NA	NA	NA	+	NA	NA	10	1854.09	10.49	0.00
14	-0.09	0.06	NA	+	+	NA	NA	NA	NA	NA	NA	NA	8	1854.65	11.05	0.00
8	0.47	-0.13	0.01	+	NA	NA	NA	NA	NA	NA	NA	NA	8	1854.69	11.10	0.00
24	0.38	-0.11	0.01	+	NA	+	NA	NA	NA	NA	NA	NA	9	1855.43	11.84	0.00
30	-0.15	0.07	NA	+	+	+	NA	NA	NA	NA	NA	NA	9	1855.75	12.16	0.00
318	-0.12	0.08	NA	+	+	+	+	NA	NA	+	NA	NA	11	1855.78	12.18	0.00
71	0.09	NA	0.01	+	NA	NA	NA	+	NA	NA	NA	NA	8	1855.78	12.18	0.00
46	-0.13	0.06	NA	+	+	NA	+	NA	NA	NA	NA	NA	9	1856.23	12.64	0.00
4	0.54	-0.13	0.01	NA	NA	NA	NA	NA	NA	NA	NA	NA	7	1856.44	12.85	0.00
10	-0.01	0.06	NA	NA	+	NA	NA	NA	NA	NA	NA	NA	7	1856.69	13.09	0.00
7	0.15	NA	0.00	+	NA	NA	NA	NA	NA	NA	NA	NA	7	1856.86	13.27	0.00
62	-0.19	0.08	NA	+	+	+	+	NA	NA	NA	NA	NA	10	1857.23	13.64	0.00
830	-0.13	0.08	NA	+	+	+	+	NA	NA	+	+	NA	12	1857.78	14.18	0.00
42	-0.07	0.07	NA	NA	+	NA	+	NA	NA	NA	NA	NA	8	1857.83	14.23	0.00
3	0.22	NA	0.00	NA	NA	NA	NA	NA	NA	NA	NA	NA	6	1858.44	14.84	0.00
269	0.40	NA	NA	+	+	NA	NA	NA	NA	+	NA	NA	8	1858.67	15.07	0.00
13	0.33	NA	NA	+	+	NA	NA	NA	NA	NA	NA	NA	7	1860.38	16.78	0.00
6	0.05	0.05	NA	+	NA	NA	NA	NA	NA	NA	NA	NA	7	1861.40	17.80	0.00
22	-0.04	0.07	NA	+	NA	+	NA	NA	NA	NA	NA	NA	8	1861.79	18.20	0.00
2	0.12	0.05	NA	NA	NA	NA	NA	NA	NA	NA	NA	NA	6	1862.90	19.30	0.00
9	0.42	NA	NA	NA	+	NA	NA	NA	NA	NA	NA	NA	6	1863.43	19.84	0.00
5	0.43	NA	NA	+	NA	NA	NA	NA	NA	NA	NA	NA	6	1865.99	22.40	0.00
1	0.50	NA	NA	NA	NA	NA	NA	NA	NA	NA	NA	NA	5	1868.50	24.90	0.00

HEMAGGLUTINATION SCORE

	I	AL	P	S	AL:P	AL:S	P:S	AL:P:S	NA	NA	NA	NA	df	AIC	delta	weight
12	3.78	0.05	+	NA	+	NA	NA	NA	NA	NA	NA	NA	8	2042.86	0.00	0.20
1	4.06	NA	NA	NA	NA	NA	NA	NA	NA	NA	NA	NA	5	2043.22	0.36	0.17
16	3.75	0.05	+	+	+	NA	NA	NA	NA	NA	NA	NA	9	2044.41	1.55	0.09
5	4.02	NA	NA	+	NA	NA	NA	NA	NA	NA	NA	NA	6	2044.46	1.61	0.09
2	3.99	0.01	NA	NA	NA	NA	NA	NA	NA	NA	NA	NA	6	2044.67	1.81	0.08
3	4.05	NA	+	NA	NA	NA	NA	NA	NA	NA	NA	NA	6	2045.13	2.28	0.06
6	3.93	0.02	NA	+	NA	NA	NA	NA	NA	NA	NA	NA	7	2045.71	2.86	0.05
48	3.78	0.05	+	+	+	NA	+	NA	NA	NA	NA	NA	10	2045.79	2.94	0.05
7	4.01	NA	+	+	NA	NA	NA	NA	NA	NA	NA	NA	7	2046.37	3.52	0.03
32	3.76	0.05	+	+	+	+	NA	NA	NA	NA	NA	NA	10	2046.44	3.59	0.03
4	3.97	0.01	+	NA	NA	NA	NA	NA	NA	NA	NA	NA	7	2046.59	3.73	0.03
39	4.05	NA	+	+	NA	NA	+	NA	NA	NA	NA	NA	8	2047.33	4.47	0.02
8	3.92	0.02	+	+	NA	NA	NA	NA	NA	NA	NA	NA	8	2047.62	4.76	0.02
22	3.95	0.01	NA	+	NA	+	NA	NA	NA	NA	NA	NA	8	2047.68	4.83	0.02
64	3.80	0.05	+	+	+	+	+	NA	NA	NA	NA	NA	11	2047.80	4.95	0.02
40	3.95	0.02	+	+	NA	NA	+	NA	NA	NA	NA	NA	9	2048.32	5.47	0.01
128	3.84	0.04	+	+	+	+	+	+	NA	NA	NA	NA	12	2049.26	6.40	0.01
24	3.94	0.01	+	+	NA	+	NA	NA	NA	NA	NA	NA	9	2049.57	6.71	0.01
56	3.98	0.01	+	+	NA	+	+	NA	NA	NA	NA	NA	10	2050.21	7.35	0.01
	I	AF	P	S	AF:P	AF:S	P:S	AF:P:S	NA	NA	NA	NA	df	AIC	delta	weight
1	4.06	NA	NA	NA	NA	NA	NA	NA	NA	NA	NA	NA	5	2043.22	0.00	0.44
5	4.02	NA	NA	+	NA	NA	NA	NA	NA	NA	NA	NA	6	2044.46	1.24	0.24
3	4.05	NA	+	NA	NA	NA	NA	NA	NA	NA	NA	NA	6	2045.13	1.91	0.17
7	4.01	NA	+	+	NA	NA	NA	NA	NA	NA	NA	NA	7	2046.37	3.15	0.09
39	4.05	NA	+	+	NA	NA	+	NA	NA	NA	NA	NA	8	2047.33	4.11	0.06
2	4.07	+	NA	NA	NA	NA	NA	NA	NA	NA	NA	NA	15	2055.79	12.57	0.00
6	4.02	+	NA	+	NA	NA	NA	NA	NA	NA	NA	NA	16	2056.81	13.59	0.00
12	3.89	+	+	NA	+	NA	NA	NA	NA	NA	NA	NA	26	2057.06	13.84	0.00
4	4.04	+	+	NA	NA	NA	NA	NA	NA	NA	NA	NA	16	2057.68	14.46	0.00
8	4.00	+	+	+	NA	NA	NA	NA	NA	NA	NA	NA	17	2058.68	15.46	0.00
16	3.86	+	+	+	+	NA	NA	NA	NA	NA	NA	NA	27	2058.75	15.54	0.00
40	4.04	+	+	+	NA	NA	+	NA	NA	NA	NA	NA	18	2059.08	15.86	0.00
48	3.89	+	+	+	+	NA	+	NA	NA	NA	NA	NA	28	2059.90	16.68	0.00
22	3.97	+	NA	+	NA	+	NA	NA	NA	NA	NA	NA	26	2072.64	29.42	0.00
24	3.95	+	+	+	NA	+	NA	NA	NA	NA	NA	NA	27	2074.57	31.35	0.00
56	3.99	+	+	+	NA	+	+	NA	NA	NA	NA	NA	28	2074.75	31.53	0.00
32	3.82	+	+	+	+	+	NA	NA	NA	NA	NA	NA	37	2077.67	34.45	0.00
64	3.86	+	+	+	+	+	+	NA	NA	NA	NA	NA	38	2079.01	35.79	0.00
128	3.95	+	+	+	+	+	+	+	NA	NA	NA	NA	46	2091.70	48.48	0.00
	I	AT	P	S	AT:P	AT:S	P:S	AT:P:S	NA	NA	NA	NA	df	AIC	delta	weight
**12**	**2.43**	**0.19**	**+**	**NA**	**+**	**NA**	**NA**	**NA**	**NA**	**NA**	**NA**	**NA**	**8**	**2038.76**	**0.00**	**0.37**
16	2.36	0.20	+	+	+	NA	NA	NA	NA	NA	NA	NA	9	2040.07	1.31	0.19
32	2.60	0.17	+	+	+	+	NA	NA	NA	NA	NA	NA	10	2041.61	2.86	0.09
48	2.41	0.19	+	+	+	NA	+	NA	NA	NA	NA	NA	10	2041.64	2.89	0.09
64	2.70	0.16	+	+	+	+	+	NA	NA	NA	NA	NA	11	2043.00	4.25	0.04
1	4.06	NA	NA	NA	NA	NA	NA	NA	NA	NA	NA	NA	5	2043.22	4.46	0.04
2	3.53	0.06	NA	NA	NA	NA	NA	NA	NA	NA	NA	NA	6	2043.84	5.08	0.03
5	4.02	NA	NA	+	NA	NA	NA	NA	NA	NA	NA	NA	6	2044.46	5.71	0.02
22	3.76	0.03	NA	+	NA	+	NA	NA	NA	NA	NA	NA	8	2044.65	5.89	0.02
6	3.42	0.07	NA	+	NA	NA	NA	NA	NA	NA	NA	NA	7	2044.76	6.01	0.02
128	2.69	0.16	+	+	+	+	+	+	NA	NA	NA	NA	12	2045.06	6.31	0.02
3	4.05	NA	+	NA	NA	NA	NA	NA	NA	NA	NA	NA	6	2045.13	6.38	0.02
4	3.51	0.06	+	NA	NA	NA	NA	NA	NA	NA	NA	NA	7	2045.71	6.95	0.01
7	4.01	NA	+	+	NA	NA	NA	NA	NA	NA	NA	NA	7	2046.37	7.62	0.01
24	3.73	0.03	+	+	NA	+	NA	NA	NA	NA	NA	NA	9	2046.43	7.67	0.01
8	3.39	0.07	+	+	NA	NA	NA	NA	NA	NA	NA	NA	8	2046.62	7.86	0.01
56	3.79	0.03	+	+	NA	+	+	NA	NA	NA	NA	NA	10	2046.85	8.09	0.01
39	4.05	NA	+	+	NA	NA	+	NA	NA	NA	NA	NA	8	2047.33	8.57	0.01
40	3.42	0.07	+	+	NA	NA	+	NA	NA	NA	NA	NA	9	2047.46	8.70	0.00
	I	AL	I(AL^2)	P	S	AL:P	AL:S	I(AL^2):P	I(AL^2):S	P:S	AL:P:S	I(AL^2):P:S	df	AIC	delta	weight
71	3.90	NA	0.00	+	NA	NA	NA	+	NA	NA	NA	NA	8	2041.95	0.00	0.12
22	3.78	0.05	NA	+	NA	+	NA	NA	NA	NA	NA	NA	8	2042.86	0.91	0.08
1	4.06	NA	NA	NA	NA	NA	NA	NA	NA	NA	NA	NA	5	2043.22	1.27	0.06
79	3.86	NA	0.00	+	+	NA	NA	+	NA	NA	NA	NA	9	2043.45	1.50	0.06
72	3.94	-0.02	0.01	+	NA	NA	NA	+	NA	NA	NA	NA	9	2043.95	2.00	0.04
30	3.75	0.05	NA	+	+	+	NA	NA	NA	NA	NA	NA	9	2044.41	2.46	0.03
9	4.02	NA	NA	NA	+	NA	NA	NA	NA	NA	NA	NA	6	2044.46	2.52	0.03
3	4.02	NA	0.00	NA	NA	NA	NA	NA	NA	NA	NA	NA	6	2044.49	2.54	0.03
2	3.99	0.01	NA	NA	NA	NA	NA	NA	NA	NA	NA	NA	6	2044.67	2.72	0.03
24	3.86	0.02	0.00	+	NA	+	NA	NA	NA	NA	NA	NA	9	2044.71	2.76	0.03
335	3.89	NA	0.00	+	+	NA	NA	+	NA	+	NA	NA	10	2044.93	2.98	0.03
5	4.05	NA	NA	+	NA	NA	NA	NA	NA	NA	NA	NA	6	2045.13	3.19	0.02
207	3.88	NA	0.00	+	+	NA	NA	+	+	NA	NA	NA	10	2045.31	3.36	0.02
80	3.91	-0.02	0.01	+	+	NA	NA	+	NA	NA	NA	NA	10	2045.44	3.50	0.02
11	3.96	NA	0.00	NA	+	NA	NA	NA	NA	NA	NA	NA	7	2045.47	3.53	0.02
10	3.93	0.02	NA	NA	+	NA	NA	NA	NA	NA	NA	NA	7	2045.71	3.77	0.02
286	3.78	0.05	NA	+	+	+	NA	NA	NA	+	NA	NA	10	2045.79	3.84	0.02
88	4.00	-0.04	0.01	+	NA	+	NA	+	NA	NA	NA	NA	10	2045.87	3.92	0.02
32	3.83	0.01	0.00	+	+	+	NA	NA	NA	NA	NA	NA	10	2046.21	4.26	0.01
13	4.01	NA	NA	+	+	NA	NA	NA	NA	NA	NA	NA	7	2046.37	4.43	0.01
7	4.00	NA	0.00	+	NA	NA	NA	NA	NA	NA	NA	NA	7	2046.40	4.45	0.01
4	4.08	-0.02	0.00	NA	NA	NA	NA	NA	NA	NA	NA	NA	7	2046.41	4.47	0.01
62	3.76	0.05	NA	+	+	+	+	NA	NA	NA	NA	NA	10	2046.44	4.50	0.01
6	3.97	0.01	NA	+	NA	NA	NA	NA	NA	NA	NA	NA	7	2046.59	4.64	0.01
463	3.91	NA	0.00	+	+	NA	NA	+	+	+	NA	NA	11	2046.69	4.75	0.01
336	3.93	-0.02	0.01	+	+	NA	NA	+	NA	+	NA	NA	11	2046.96	5.01	0.01
139	3.99	NA	0.00	NA	+	NA	NA	NA	+	NA	NA	NA	8	2046.98	5.04	0.01
208	3.95	-0.03	0.01	+	+	NA	NA	+	+	NA	NA	NA	11	2047.23	5.29	0.01
269	4.05	NA	NA	+	+	NA	NA	NA	NA	+	NA	NA	8	2047.33	5.38	0.01
96	3.98	-0.05	0.01	+	+	+	NA	+	NA	NA	NA	NA	11	2047.34	5.39	0.01
15	3.95	NA	0.00	+	+	NA	NA	NA	NA	NA	NA	NA	8	2047.37	5.42	0.01
12	4.03	-0.03	0.00	NA	+	NA	NA	NA	NA	NA	NA	NA	8	2047.38	5.44	0.01
112	3.93	-0.03	0.01	+	+	NA	+	+	NA	NA	NA	NA	11	2047.47	5.52	0.01
14	3.92	0.02	NA	+	+	NA	NA	NA	NA	NA	NA	NA	8	2047.62	5.67	0.01
288	3.86	0.02	0.00	+	+	+	NA	NA	NA	+	NA	NA	11	2047.64	5.70	0.01
42	3.95	0.01	NA	NA	+	NA	+	NA	NA	NA	NA	NA	8	2047.68	5.74	0.01
240	3.79	0.04	0.00	+	+	NA	+	+	+	NA	NA	NA	12	2047.80	5.85	0.01
318	3.80	0.05	NA	+	+	+	+	NA	NA	+	NA	NA	11	2047.80	5.86	0.01
160	3.88	0.00	0.00	+	+	+	NA	NA	+	NA	NA	NA	11	2047.90	5.95	0.01
271	3.99	NA	0.00	+	+	NA	NA	NA	NA	+	NA	NA	9	2048.09	6.14	0.01
64	3.86	0.01	0.00	+	+	+	+	NA	NA	NA	NA	NA	11	2048.20	6.25	0.01
192	3.72	0.07	0.00	+	+	+	+	NA	+	NA	NA	NA	12	2048.29	6.34	0.01
8	4.06	-0.03	0.00	+	NA	NA	NA	NA	NA	NA	NA	NA	8	2048.32	6.37	0.00
270	3.95	0.02	NA	+	+	NA	NA	NA	NA	+	NA	NA	9	2048.32	6.38	0.00
172	3.88	0.04	0.00	NA	+	NA	+	NA	+	NA	NA	NA	10	2048.44	6.49	0.00
1487	3.92	NA	0.00	+	+	NA	NA	+	+	+	NA	+	12	2048.58	6.64	0.00
464	3.97	-0.02	0.01	+	+	NA	NA	+	+	+	NA	NA	12	2048.66	6.72	0.00
140	4.09	-0.04	0.00	NA	+	NA	NA	NA	+	NA	NA	NA	9	2048.76	6.81	0.00
143	3.97	NA	0.00	+	+	NA	NA	NA	+	NA	NA	NA	9	2048.83	6.88	0.00
352	3.99	-0.04	0.01	+	+	+	NA	+	NA	+	NA	NA	12	2048.88	6.93	0.00
368	3.96	-0.02	0.01	+	+	NA	+	+	NA	+	NA	NA	12	2048.96	7.01	0.00
496	3.81	0.04	0.00	+	+	NA	+	+	+	+	NA	NA	13	2049.09	7.15	0.00
224	4.00	-0.05	0.01	+	+	+	NA	+	+	NA	NA	NA	12	2049.18	7.24	0.00
416	3.91	0.00	0.00	+	+	+	NA	NA	+	+	NA	NA	12	2049.22	7.28	0.00
830	3.84	0.04	NA	+	+	+	+	NA	NA	+	+	NA	12	2049.26	7.31	0.00
44	4.07	-0.04	0.00	NA	+	NA	+	NA	NA	NA	NA	NA	9	2049.26	7.31	0.00
16	4.02	-0.03	0.00	+	+	NA	NA	NA	NA	NA	NA	NA	9	2049.27	7.32	0.00
399	4.01	NA	0.00	+	+	NA	NA	NA	+	+	NA	NA	10	2049.36	7.41	0.00
128	3.99	-0.05	0.01	+	+	+	+	+	NA	NA	NA	NA	12	2049.38	7.44	0.00
448	3.75	0.08	0.00	+	+	+	+	NA	+	+	NA	NA	13	2049.48	7.53	0.00
46	3.94	0.01	NA	+	+	NA	+	NA	NA	NA	NA	NA	9	2049.57	7.62	0.00
320	3.90	0.01	0.00	+	+	+	+	NA	NA	+	NA	NA	12	2049.60	7.65	0.00
256	3.83	0.02	0.00	+	+	+	+	+	+	NA	NA	NA	13	2049.83	7.88	0.00
272	4.04	-0.02	0.00	+	+	NA	NA	NA	NA	+	NA	NA	10	2050.07	8.12	0.00
302	3.98	0.01	NA	+	+	NA	+	NA	NA	+	NA	NA	10	2050.21	8.26	0.00
176	3.87	0.04	0.00	+	+	NA	+	NA	+	NA	NA	NA	11	2050.37	8.42	0.00
144	4.07	-0.04	0.00	+	+	NA	NA	NA	+	NA	NA	NA	10	2050.58	8.63	0.00
1488	3.97	-0.02	0.00	+	+	NA	NA	+	+	+	NA	+	13	2050.60	8.65	0.00
480	4.02	-0.04	0.01	+	+	+	NA	+	+	+	NA	NA	13	2050.64	8.70	0.00
2048	4.10	-0.07	0.01	+	+	+	+	+	+	+	+	+	16	2050.64	8.70	0.00
432	3.89	0.05	0.00	+	+	NA	+	NA	+	+	NA	NA	12	2050.84	8.90	0.00
384	4.01	-0.05	0.01	+	+	+	+	+	NA	+	NA	NA	13	2050.91	8.96	0.00
48	4.06	-0.04	0.00	+	+	NA	+	NA	NA	NA	NA	NA	10	2051.11	9.16	0.00
512	3.85	0.03	0.00	+	+	+	+	+	+	+	NA	NA	14	2051.15	9.20	0.00
832	3.92	0.01	0.00	+	+	+	+	NA	NA	+	+	NA	13	2051.16	9.21	0.00
1520	3.82	0.04	0.00	+	+	NA	+	+	+	+	NA	+	14	2051.17	9.23	0.00
400	4.10	-0.04	0.00	+	+	NA	NA	NA	+	+	NA	NA	11	2051.21	9.26	0.00
960	3.77	0.07	0.00	+	+	+	+	NA	+	+	+	NA	14	2051.36	9.42	0.00
304	4.09	-0.04	0.00	+	+	NA	+	NA	NA	+	NA	NA	11	2051.83	9.89	0.00
896	4.08	-0.06	0.01	+	+	+	+	+	NA	+	+	NA	14	2052.03	10.09	0.00
1504	4.04	-0.05	0.01	+	+	+	NA	+	+	+	NA	+	14	2052.50	10.55	0.00
1024	3.91	0.01	0.00	+	+	+	+	+	+	+	+	NA	15	2052.79	10.84	0.00
1536	3.86	0.03	0.00	+	+	+	+	+	+	+	NA	+	15	2053.21	11.27	0.00

HEMOLYSIS SCORE

	I	AL	P	S	AL:P	AL:S	P:S	AL:P:S	NA	NA	NA	NA	df	AIC	delta	weight
4	2.20	0.02	+	NA	NA	NA	NA	NA	NA	NA	NA	NA	7	1767.08	0.00	0.19
3	2.30	NA	+	NA	NA	NA	NA	NA	NA	NA	NA	NA	6	1767.34	0.26	0.16
40	2.23	0.02	+	+	NA	NA	+	NA	NA	NA	NA	NA	9	1768.04	0.96	0.11
12	2.17	0.03	+	NA	+	NA	NA	NA	NA	NA	NA	NA	8	1768.97	1.89	0.07
39	2.36	NA	+	+	NA	NA	+	NA	NA	NA	NA	NA	8	1768.99	1.91	0.07
8	2.19	0.02	+	+	NA	NA	NA	NA	NA	NA	NA	NA	8	1769.13	2.04	0.07
7	2.31	NA	+	+	NA	NA	NA	NA	NA	NA	NA	NA	7	1769.36	2.28	0.06
56	2.26	0.02	+	+	NA	+	+	NA	NA	NA	NA	NA	10	1769.85	2.77	0.05
48	2.23	0.03	+	+	+	NA	+	NA	NA	NA	NA	NA	10	1770.09	3.01	0.04
2	2.11	0.02	NA	NA	NA	NA	NA	NA	NA	NA	NA	NA	6	1770.43	3.35	0.03
1	2.22	NA	NA	NA	NA	NA	NA	NA	NA	NA	NA	NA	5	1770.71	3.63	0.03
16	2.17	0.03	+	+	+	NA	NA	NA	NA	NA	NA	NA	9	1771.02	3.94	0.03
24	2.21	0.02	+	+	NA	+	NA	NA	NA	NA	NA	NA	9	1771.05	3.97	0.03
64	2.25	0.02	+	+	+	+	+	NA	NA	NA	NA	NA	11	1771.91	4.83	0.02
6	2.10	0.02	NA	+	NA	NA	NA	NA	NA	NA	NA	NA	7	1772.44	5.36	0.01
5	2.22	NA	NA	+	NA	NA	NA	NA	NA	NA	NA	NA	6	1772.75	5.67	0.01
32	2.19	0.02	+	+	+	+	NA	NA	NA	NA	NA	NA	10	1772.98	5.89	0.01
128	2.22	0.03	+	+	+	+	+	+	NA	NA	NA	NA	12	1773.42	6.33	0.01
22	2.14	0.01	NA	+	NA	+	NA	NA	NA	NA	NA	NA	8	1774.08	7.00	0.01
	I	AF	P	S	AF:P	AF:S	P:S	AF:P:S	NA	NA	NA	NA	df	AIC	delta	weight
3	2.30	NA	+	NA	NA	NA	NA	NA	NA	NA	NA	NA	6	1767.34	0.00	0.48
39	2.36	NA	+	+	NA	NA	+	NA	NA	NA	NA	NA	8	1768.99	1.65	0.21
7	2.31	NA	+	+	NA	NA	NA	NA	NA	NA	NA	NA	7	1769.36	2.02	0.17
1	2.22	NA	NA	NA	NA	NA	NA	NA	NA	NA	NA	NA	5	1770.71	3.37	0.09
5	2.22	NA	NA	+	NA	NA	NA	NA	NA	NA	NA	NA	6	1772.75	5.41	0.03
4	2.28	+	+	NA	NA	NA	NA	NA	NA	NA	NA	NA	16	1776.94	9.60	0.00
40	2.33	+	+	+	NA	NA	+	NA	NA	NA	NA	NA	18	1777.80	10.45	0.00
8	2.28	+	+	+	NA	NA	NA	NA	NA	NA	NA	NA	17	1779.05	11.70	0.00
2	2.21	+	NA	NA	NA	NA	NA	NA	NA	NA	NA	NA	15	1779.20	11.86	0.00
12	2.21	+	+	NA	+	NA	NA	NA	NA	NA	NA	NA	26	1779.95	12.61	0.00
48	2.27	+	+	+	+	NA	+	NA	NA	NA	NA	NA	28	1780.63	13.28	0.00
6	2.20	+	NA	+	NA	NA	NA	NA	NA	NA	NA	NA	16	1781.29	13.95	0.00
16	2.21	+	+	+	+	NA	NA	NA	NA	NA	NA	NA	27	1782.12	14.78	0.00
64	2.32	+	+	+	+	+	+	NA	NA	NA	NA	NA	38	1785.35	18.01	0.00
32	2.27	+	+	+	+	+	NA	NA	NA	NA	NA	NA	37	1785.98	18.64	0.00
56	2.39	+	+	+	NA	+	+	NA	NA	NA	NA	NA	28	1787.25	19.91	0.00
24	2.34	+	+	+	NA	+	NA	NA	NA	NA	NA	NA	27	1788.32	20.98	0.00
22	2.28	+	NA	+	NA	+	NA	NA	NA	NA	NA	NA	26	1790.37	23.02	0.00
128	2.34	+	+	+	+	+	+	+	NA	NA	NA	NA	46	1796.50	29.16	0.00
	I	AT	P	S	AT:P	AT:S	P:S	AT:P:S	NA	NA	NA	NA	df	AIC	delta	weight
12	-1.52	0.38	+	NA	+	NA	NA	NA	NA	NA	NA	NA	8	1761.33	0.00	0.41
16	-1.52	0.38	+	+	+	NA	NA	NA	NA	NA	NA	NA	9	1763.38	2.05	0.15
48	-1.38	0.37	+	+	+	NA	+	NA	NA	NA	NA	NA	10	1763.71	2.38	0.12
32	-1.31	0.36	+	+	+	+	NA	NA	NA	NA	NA	NA	10	1765.37	4.04	0.05
64	-0.99	0.33	+	+	+	+	+	NA	NA	NA	NA	NA	11	1765.55	4.22	0.05
128	-0.99	0.33	+	+	+	+	+	+	NA	NA	NA	NA	11	1765.55	4.22	0.05
4	0.42	0.19	+	NA	NA	NA	NA	NA	NA	NA	NA	NA	7	1765.83	4.50	0.04
3	2.30	NA	+	NA	NA	NA	NA	NA	NA	NA	NA	NA	6	1767.34	6.01	0.02
40	0.44	0.19	+	+	NA	NA	+	NA	NA	NA	NA	NA	9	1767.40	6.07	0.02
56	1.05	0.13	+	+	NA	+	+	NA	NA	NA	NA	NA	10	1767.78	6.45	0.02
8	0.41	0.19	+	+	NA	NA	NA	NA	NA	NA	NA	NA	8	1767.87	6.54	0.02
24	0.93	0.14	+	+	NA	+	NA	NA	NA	NA	NA	NA	9	1768.68	7.35	0.01
2	0.21	0.20	NA	NA	NA	NA	NA	NA	NA	NA	NA	NA	6	1768.70	7.37	0.01
39	2.36	NA	+	+	NA	NA	+	NA	NA	NA	NA	NA	8	1768.99	7.66	0.01
7	2.31	NA	+	+	NA	NA	NA	NA	NA	NA	NA	NA	7	1769.36	8.03	0.01
1	2.22	NA	NA	NA	NA	NA	NA	NA	NA	NA	NA	NA	5	1770.71	9.38	0.00
6	0.19	0.20	NA	+	NA	NA	NA	NA	NA	NA	NA	NA	7	1770.72	9.39	0.00
22	0.76	0.14	NA	+	NA	+	NA	NA	NA	NA	NA	NA	8	1771.28	9.95	0.00
5	2.22	NA	NA	+	NA	NA	NA	NA	NA	NA	NA	NA	6	1772.75	11.42	0.00
	I	AL	I(AL^2)	P	S	AL:P	AL:S	I(AL^2):P	I(AL^2):S	P:S	AL:P:S	I(AL^2):P:S	df	AIC	delta	weight
7	2.24	NA	0.00	+	NA	NA	NA	NA	NA	NA	NA	NA	7	1766.29	0.00	0.10
6	2.20	0.02	NA	+	NA	NA	NA	NA	NA	NA	NA	NA	7	1767.08	0.79	0.06
271	2.28	NA	0.00	+	+	NA	NA	NA	NA	+	NA	NA	9	1767.27	0.97	0.06
5	2.30	NA	NA	+	NA	NA	NA	NA	NA	NA	NA	NA	6	1767.34	1.05	0.06
8	2.35	-0.05	0.01	+	NA	NA	NA	NA	NA	NA	NA	NA	8	1767.67	1.38	0.05
71	2.22	NA	0.00	+	NA	NA	NA	+	NA	NA	NA	NA	8	1768.03	1.74	0.04
270	2.23	0.02	NA	+	+	NA	NA	NA	NA	+	NA	NA	9	1768.04	1.75	0.04
15	2.23	NA	0.00	+	+	NA	NA	NA	NA	NA	NA	NA	8	1768.32	2.03	0.03
399	2.30	NA	0.00	+	+	NA	NA	NA	+	+	NA	NA	10	1768.89	2.60	0.03
272	2.37	-0.04	0.00	+	+	NA	NA	NA	NA	+	NA	NA	10	1768.91	2.62	0.03
22	2.17	0.03	NA	+	NA	+	NA	NA	NA	NA	NA	NA	8	1768.97	2.68	0.03
269	2.36	NA	NA	+	+	NA	NA	NA	NA	+	NA	NA	8	1768.99	2.70	0.02
14	2.19	0.02	NA	+	+	NA	NA	NA	NA	NA	NA	NA	8	1769.13	2.83	0.02
335	2.27	NA	0.00	+	+	NA	NA	+	NA	+	NA	NA	10	1769.27	2.98	0.02
13	2.31	NA	NA	+	+	NA	NA	NA	NA	NA	NA	NA	7	1769.36	3.07	0.02
72	2.33	-0.05	0.01	+	NA	NA	NA	+	NA	NA	NA	NA	9	1769.47	3.18	0.02
3	2.15	NA	0.00	NA	NA	NA	NA	NA	NA	NA	NA	NA	6	1769.57	3.28	0.02
24	2.33	-0.04	0.01	+	NA	+	NA	NA	NA	NA	NA	NA	9	1769.62	3.32	0.02
16	2.35	-0.05	0.01	+	+	NA	NA	NA	NA	NA	NA	NA	9	1769.71	3.41	0.02
302	2.26	0.02	NA	+	+	NA	+	NA	NA	+	NA	NA	10	1769.85	3.56	0.02
79	2.21	NA	0.00	+	+	NA	NA	+	NA	NA	NA	NA	9	1770.09	3.80	0.01
286	2.23	0.03	NA	+	+	+	NA	NA	NA	+	NA	NA	10	1770.09	3.80	0.01
143	2.24	NA	0.00	+	+	NA	NA	NA	+	NA	NA	NA	9	1770.14	3.85	0.01
400	2.41	-0.05	0.01	+	+	NA	NA	NA	+	+	NA	NA	11	1770.33	4.04	0.01
304	2.43	-0.05	0.01	+	+	NA	+	NA	NA	+	NA	NA	11	1770.41	4.12	0.01
2	2.11	0.02	NA	NA	NA	NA	NA	NA	NA	NA	NA	NA	6	1770.43	4.14	0.01
1	2.22	NA	NA	NA	NA	NA	NA	NA	NA	NA	NA	NA	5	1770.71	4.42	0.01
4	2.28	-0.05	0.01	NA	NA	NA	NA	NA	NA	NA	NA	NA	7	1770.81	4.52	0.01
336	2.36	-0.04	0.01	+	+	NA	NA	+	NA	+	NA	NA	11	1770.93	4.64	0.01
463	2.29	NA	0.00	+	+	NA	NA	+	+	+	NA	NA	11	1770.94	4.65	0.01
288	2.37	-0.04	0.00	+	+	+	NA	NA	NA	+	NA	NA	11	1770.98	4.68	0.01
30	2.17	0.03	NA	+	+	+	NA	NA	NA	NA	NA	NA	9	1771.02	4.73	0.01
46	2.21	0.02	NA	+	+	NA	+	NA	NA	NA	NA	NA	9	1771.05	4.76	0.01
88	2.42	-0.08	0.01	+	NA	+	NA	+	NA	NA	NA	NA	10	1771.08	4.79	0.01
144	2.38	-0.06	0.01	+	+	NA	NA	NA	+	NA	NA	NA	10	1771.31	5.02	0.01
48	2.40	-0.06	0.01	+	+	NA	+	NA	NA	NA	NA	NA	10	1771.36	5.07	0.01
80	2.33	-0.05	0.01	+	+	NA	NA	+	NA	NA	NA	NA	10	1771.53	5.24	0.01
11	2.14	NA	0.00	NA	+	NA	NA	NA	NA	NA	NA	NA	7	1771.56	5.27	0.01
32	2.32	-0.04	0.01	+	+	+	NA	NA	NA	NA	NA	NA	10	1771.67	5.38	0.01
1487	2.27	NA	0.00	+	+	NA	NA	+	+	+	NA	+	12	1771.75	5.46	0.01
318	2.25	0.02	NA	+	+	+	+	NA	NA	+	NA	NA	11	1771.91	5.62	0.01
207	2.23	NA	0.00	+	+	NA	NA	+	+	NA	NA	NA	10	1771.97	5.68	0.01
432	2.40	-0.04	0.00	+	+	NA	+	NA	+	+	NA	NA	12	1772.39	6.10	0.00
464	2.41	-0.05	0.01	+	+	NA	NA	+	+	+	NA	NA	12	1772.40	6.11	0.00
416	2.42	-0.05	0.01	+	+	+	NA	NA	+	+	NA	NA	12	1772.40	6.11	0.00
10	2.10	0.02	NA	NA	+	NA	NA	NA	NA	NA	NA	NA	7	1772.44	6.15	0.00
368	2.42	-0.05	0.01	+	+	NA	+	+	NA	+	NA	NA	12	1772.48	6.18	0.00
320	2.43	-0.05	0.01	+	+	+	+	NA	NA	+	NA	NA	12	1772.49	6.20	0.00
352	2.44	-0.07	0.01	+	+	+	NA	+	NA	+	NA	NA	12	1772.61	6.32	0.00
9	2.22	NA	NA	NA	+	NA	NA	NA	NA	NA	NA	NA	6	1772.75	6.46	0.00
12	2.27	-0.05	0.01	NA	+	NA	NA	NA	NA	NA	NA	NA	8	1772.80	6.51	0.00
1488	2.41	-0.06	0.01	+	+	NA	NA	+	+	+	NA	+	13	1772.86	6.57	0.00
62	2.19	0.02	NA	+	+	+	+	NA	NA	NA	NA	NA	10	1772.98	6.69	0.00
139	2.16	NA	0.00	NA	+	NA	NA	NA	+	NA	NA	NA	8	1773.08	6.79	0.00
96	2.42	-0.08	0.01	+	+	+	NA	+	NA	NA	NA	NA	11	1773.13	6.84	0.00
208	2.36	-0.05	0.01	+	+	NA	NA	+	+	NA	NA	NA	11	1773.23	6.93	0.00
112	2.38	-0.06	0.01	+	+	NA	+	+	NA	NA	NA	NA	11	1773.25	6.96	0.00
160	2.36	-0.05	0.01	+	+	+	NA	NA	+	NA	NA	NA	11	1773.32	7.03	0.00
64	2.38	-0.06	0.01	+	+	+	+	NA	NA	NA	NA	NA	11	1773.36	7.07	0.00
176	2.37	-0.05	0.01	+	+	NA	+	NA	+	NA	NA	NA	11	1773.37	7.08	0.00
830	2.22	0.03	NA	+	+	+	+	NA	NA	+	+	NA	12	1773.42	7.12	0.00
832	2.41	-0.05	0.01	+	+	+	+	NA	NA	+	+	NA	13	1773.73	7.44	0.00
44	2.35	-0.07	0.01	NA	+	NA	+	NA	NA	NA	NA	NA	9	1773.98	7.69	0.00
140	2.32	-0.06	0.01	NA	+	NA	NA	NA	+	NA	NA	NA	9	1773.98	7.69	0.00
42	2.14	0.01	NA	NA	+	NA	+	NA	NA	NA	NA	NA	8	1774.08	7.79	0.00
480	2.48	-0.07	0.01	+	+	+	NA	+	+	+	NA	NA	13	1774.18	7.89	0.00
384	2.49	-0.08	0.01	+	+	+	+	+	NA	+	NA	NA	13	1774.23	7.94	0.00
448	2.40	-0.04	0.00	+	+	+	+	NA	+	+	NA	NA	13	1774.46	8.17	0.00
496	2.40	-0.04	0.00	+	+	NA	+	+	+	+	NA	NA	13	1774.47	8.17	0.00
1520	2.37	-0.04	0.01	+	+	NA	+	+	+	+	NA	+	14	1774.78	8.49	0.00
1504	2.44	-0.07	0.01	+	+	+	NA	+	+	+	NA	+	14	1774.90	8.61	0.00
224	2.44	-0.08	0.01	+	+	+	NA	+	+	NA	NA	NA	12	1774.92	8.63	0.00
128	2.45	-0.09	0.01	+	+	+	+	+	NA	NA	NA	NA	12	1774.92	8.63	0.00
240	2.36	-0.05	0.01	+	+	NA	+	+	+	NA	NA	NA	12	1775.30	9.01	0.00
192	2.36	-0.05	0.01	+	+	+	+	NA	+	NA	NA	NA	12	1775.40	9.11	0.00
960	2.36	-0.03	0.00	+	+	+	+	NA	+	+	+	NA	14	1775.56	9.27	0.00
896	2.45	-0.07	0.01	+	+	+	+	+	NA	+	+	NA	14	1775.69	9.40	0.00
172	2.34	-0.07	0.01	NA	+	NA	+	NA	+	NA	NA	NA	10	1776.03	9.74	0.00
512	2.47	-0.07	0.01	+	+	+	+	+	+	+	NA	NA	14	1776.26	9.97	0.00
1536	2.39	-0.05	0.01	+	+	+	+	+	+	+	NA	+	15	1776.86	10.57	0.00
256	2.44	-0.09	0.01	+	+	+	+	+	+	NA	NA	NA	13	1777.00	10.71	0.00
2048	2.52	-0.10	0.01	+	+	+	+	+	+	+	+	+	16	1777.21	10.92	0.00
1024	2.40	-0.05	0.01	+	+	+	+	+	+	+	+	NA	15	1777.59	11.30	0.00

Table S6. Set of models fitted to assess senescence patterns of 4 parasitic traits. We tested the effect of population ("P"), sex ("S), 4 age functions (linear "AL", factor "AF", threshold "AT" and quadratic "I(AL^2)") with all two and three-way interactions between them. "I" is the Intercept, "df" is the number of parameters, "delta" is the difference of AIC between the candidate model and the model having the lowest AIC, and "weight" the AIC weight of each model. We selected the model with the lowest AIC, and when some models had very similar explanatory power (delta<2), we selected the one which included fewer terms (shown in bold).

COCCIDIA ABUNDANCE

	I	AL	P	S	AL:P	AL:S	P:S	AL:P:S	NA	NA	NA	NA	df	AIC	D	weight
**3**	**1811.06**	**NA**	**+**	**NA**	**NA**	**NA**	**NA**	**NA**	**NA**	**NA**	**NA**	**NA**	**6**	**10681.60**	**0.00**	**0.17**
1	2560.37	NA	NA	NA	NA	NA	NA	NA	NA	NA	NA	NA	5	10681.95	0.36	0.14
7	2371.13	NA	+	+	NA	NA	NA	NA	NA	NA	NA	NA	7	10682.31	0.71	0.12
5	3123.42	NA	NA	+	NA	NA	NA	NA	NA	NA	NA	NA	6	10682.62	1.02	0.10
12	2343.35	-90.07	+	NA	+	NA	NA	NA	NA	NA	NA	NA	8	10683.45	1.85	0.07
4	1969.59	-24.94	+	NA	NA	NA	NA	NA	NA	NA	NA	NA	7	10683.55	1.95	0.06
2	2705.98	-22.52	NA	NA	NA	NA	NA	NA	NA	NA	NA	NA	6	10683.92	2.32	0.05
39	2112.87	NA	+	+	NA	NA	+	NA	NA	NA	NA	NA	8	10684.10	2.50	0.05
8	2535.34	-25.66	+	+	NA	NA	NA	NA	NA	NA	NA	NA	8	10684.27	2.67	0.05
16	2877.10	-89.39	+	+	+	NA	NA	NA	NA	NA	NA	NA	9	10684.28	2.68	0.04
6	3275.03	-23.28	NA	+	NA	NA	NA	NA	NA	NA	NA	NA	7	10684.58	2.98	0.04
40	2278.91	-26.48	+	+	NA	NA	+	NA	NA	NA	NA	NA	9	10686.06	4.46	0.02
48	2652.07	-89.15	+	+	+	NA	+	NA	NA	NA	NA	NA	10	10686.15	4.55	0.02
24	2459.91	-10.33	+	+	NA	+	NA	NA	NA	NA	NA	NA	9	10686.23	4.63	0.02
32	2850.06	-83.97	+	+	+	+	NA	NA	NA	NA	NA	NA	10	10686.34	4.74	0.02
22	3183.74	-5.93	NA	+	NA	+	NA	NA	NA	NA	NA	NA	8	10686.50	4.91	0.01
56	2200.22	-10.86	+	+	NA	+	+	NA	NA	NA	NA	NA	10	10688.02	6.42	0.01
64	2621.37	-83.21	+	+	+	+	+	NA	NA	NA	NA	NA	11	10688.22	6.62	0.01
128	2700.64	-99.13	+	+	+	+	+	+	NA	NA	NA	NA	12	10690.16	8.57	0.00
	I	AF	P	S	AF:P	AF:S	P:S	AF:P:S	NA	NA	NA	NA	df	AIC	delta	weight
3	1811.06	NA	+	NA	NA	NA	NA	NA	NA	NA	NA	NA	6	10681.60	0.00	0.29
1	2560.37	NA	NA	NA	NA	NA	NA	NA	NA	NA	NA	NA	5	10681.95	0.36	0.24
7	2371.13	NA	+	+	NA	NA	NA	NA	NA	NA	NA	NA	7	10682.31	0.71	0.20
5	3123.42	NA	NA	+	NA	NA	NA	NA	NA	NA	NA	NA	6	10682.62	1.02	0.18
39	2112.87	NA	+	+	NA	NA	+	NA	NA	NA	NA	NA	8	10684.10	2.50	0.08
4	2184.63	+	+	NA	NA	NA	NA	NA	NA	NA	NA	NA	16	10696.10	14.50	0.00
2	2932.06	+	NA	NA	NA	NA	NA	NA	NA	NA	NA	NA	15	10696.46	14.86	0.00
8	2729.48	+	+	+	NA	NA	NA	NA	NA	NA	NA	NA	17	10696.97	15.37	0.00
6	3480.50	+	NA	+	NA	NA	NA	NA	NA	NA	NA	NA	16	10697.28	15.68	0.00
40	2479.55	+	+	+	NA	NA	+	NA	NA	NA	NA	NA	18	10698.85	17.25	0.00
12	2635.16	+	+	NA	+	NA	NA	NA	NA	NA	NA	NA	26	10708.46	26.86	0.00
16	3160.29	+	+	+	+	NA	NA	NA	NA	NA	NA	NA	27	10709.46	27.86	0.00
48	2948.43	+	+	+	+	NA	+	NA	NA	NA	NA	NA	28	10711.50	29.90	0.00
24	3081.15	+	+	+	NA	+	NA	NA	NA	NA	NA	NA	27	10711.79	30.19	0.00
22	3820.39	+	NA	+	NA	+	NA	NA	NA	NA	NA	NA	26	10712.01	30.41	0.00
56	2814.96	+	+	+	NA	+	+	NA	NA	NA	NA	NA	28	10713.72	32.12	0.00
32	3517.49	+	+	+	+	+	NA	NA	NA	NA	NA	NA	37	10726.48	44.88	0.00
64	3293.00	+	+	+	+	+	+	NA	NA	NA	NA	NA	38	10728.59	46.99	0.00
128	3608.61	+	+	+	+	+	+	+	NA	NA	NA	NA	46	10742.07	60.47	0.00
	I	AT	P	S	AT:P	AT:S	P:S	AT: P:S	NA	NA	NA	NA	df	AIC	delta	weight
3	1811.06	NA	+	NA	NA	NA	NA	NA	NA	NA	NA	NA	6	10681.60	0.00	0.19
1	2560.37	NA	NA	NA	NA	NA	NA	NA	NA	NA	NA	NA	5	10681.95	0.36	0.16
7	2371.13	NA	+	+	NA	NA	NA	NA	NA	NA	NA	NA	7	10682.31	0.71	0.13
5	3123.42	NA	NA	+	NA	NA	NA	NA	NA	NA	NA	NA	6	10682.62	1.02	0.11
4	1573.95	24.86	+	NA	NA	NA	NA	NA	NA	NA	NA	NA	7	10683.64	2.04	0.07
12	4545.80	-290.02	+	NA	+	NA	NA	NA	NA	NA	NA	NA	8	10683.91	2.31	0.06
2	2341.49	22.99	NA	NA	NA	NA	NA	NA	NA	NA	NA	NA	6	10683.99	2.39	0.06
39	2112.87	NA	+	+	NA	NA	+	NA	NA	NA	NA	NA	8	10684.10	2.50	0.05
16	5156.73	-296.13	+	+	+	NA	NA	NA	NA	NA	NA	NA	9	10684.66	3.06	0.04
6	2973.73	15.64	NA	+	NA	NA	NA	NA	NA	NA	NA	NA	7	10684.66	3.07	0.04
24	1134.95	135.11	+	+	NA	+	NA	NA	NA	NA	NA	NA	9	10685.70	4.10	0.02
22	1904.69	133.05	NA	+	NA	+	NA	NA	NA	NA	NA	NA	8	10685.99	4.40	0.02
48	4890.73	-292.75	+	+	+	NA	+	NA	NA	NA	NA	NA	10	10686.51	4.91	0.02
32	4576.05	-233.27	+	+	+	+	NA	NA	NA	NA	NA	NA	10	10686.69	5.09	0.01
56	859.46	136.15	+	+	NA	+	+	NA	NA	NA	NA	NA	10	10687.49	5.89	0.01
64	4244.86	-223.39	+	+	+	+	+	NA	NA	NA	NA	NA	11	10688.54	6.94	0.01
128	4173.13	-215.71	+	+	+	+	+	+	NA	NA	NA	NA	12	10690.63	9.03	0.00
8	-806.68	166.04	+	+	NA	NA	NA	NA	NA	NA	NA	NA	8	10708.52	26.92	0.00
40	-1264.69	164.54	+	+	NA	NA	+	NA	NA	NA	NA	NA	9	10709.85	28.25	0.00
	I	AL	I(AL^2)	P	S	AL:P	AL:S	I(AL^2):P	I(AL^2):S	P:S	AL:P:S	I(AL^2):P:S	df	AIC	delta	weight
5	1811.06	NA	NA	+	NA	NA	NA	NA	NA	NA	NA	NA	6	10681.60	0.00	0.10
1	2560.37	NA	NA	NA	NA	NA	NA	NA	NA	NA	NA	NA	5	10681.95	0.36	0.08
13	2371.13	NA	NA	+	+	NA	NA	NA	NA	NA	NA	NA	7	10682.31	0.71	0.07
9	3123.42	NA	NA	NA	+	NA	NA	NA	NA	NA	NA	NA	6	10682.62	1.02	0.06
71	2112.37	NA	-6.73	+	NA	NA	NA	+	NA	NA	NA	NA	8	10683.27	1.67	0.04
22	2343.35	-90.07	NA	+	NA	+	NA	NA	NA	NA	NA	NA	8	10683.45	1.85	0.04
6	1969.59	-24.94	NA	+	NA	NA	NA	NA	NA	NA	NA	NA	7	10683.55	1.95	0.04
7	1841.60	NA	-0.58	+	NA	NA	NA	NA	NA	NA	NA	NA	7	10683.64	2.05	0.04
2	2705.98	-22.52	NA	NA	NA	NA	NA	NA	NA	NA	NA	NA	6	10683.92	2.32	0.03
3	2582.86	NA	-0.42	NA	NA	NA	NA	NA	NA	NA	NA	NA	6	10683.99	2.40	0.03
79	2651.70	NA	-6.77	+	+	NA	NA	+	NA	NA	NA	NA	9	10684.09	2.49	0.03
269	2112.87	NA	NA	+	+	NA	NA	NA	NA	+	NA	NA	8	10684.10	2.50	0.03
14	2535.34	-25.66	NA	+	+	NA	NA	NA	NA	NA	NA	NA	8	10684.27	2.67	0.03
30	2877.10	-89.39	NA	+	+	+	NA	NA	NA	NA	NA	NA	9	10684.28	2.68	0.03
15	2410.33	NA	-0.72	+	+	NA	NA	NA	NA	NA	NA	NA	8	10684.36	2.76	0.03
10	3275.03	-23.28	NA	NA	+	NA	NA	NA	NA	NA	NA	NA	7	10684.58	2.98	0.02
11	3154.79	NA	-0.57	NA	+	NA	NA	NA	NA	NA	NA	NA	7	10684.66	3.06	0.02
72	2295.09	-72.19	-1.48	+	NA	NA	NA	+	NA	NA	NA	NA	9	10685.20	3.60	0.02
8	2110.13	-107.47	7.26	+	NA	NA	NA	NA	NA	NA	NA	NA	8	10685.39	3.79	0.02
24	2440.96	-150.00	5.37	+	NA	+	NA	NA	NA	NA	NA	NA	9	10685.40	3.80	0.01
4	2844.48	-102.11	7.00	NA	NA	NA	NA	NA	NA	NA	NA	NA	7	10685.76	4.16	0.01
143	2345.85	NA	1.51	+	+	NA	NA	NA	+	NA	NA	NA	9	10685.96	4.37	0.01
335	2434.43	NA	-6.73	+	+	NA	NA	+	NA	+	NA	NA	10	10685.97	4.37	0.01
80	2820.02	-67.89	-1.82	+	+	NA	NA	+	NA	NA	NA	NA	10	10686.04	4.45	0.01
270	2278.91	-26.48	NA	+	+	NA	NA	NA	NA	+	NA	NA	9	10686.06	4.46	0.01
207	2614.62	NA	-5.59	+	+	NA	NA	+	+	NA	NA	NA	10	10686.09	4.50	0.01
16	2659.09	-102.13	6.73	+	+	NA	NA	NA	NA	NA	NA	NA	9	10686.14	4.54	0.01
286	2652.07	-89.15	NA	+	+	+	NA	NA	NA	+	NA	NA	10	10686.15	4.55	0.01
271	2154.20	NA	-0.78	+	+	NA	NA	NA	NA	+	NA	NA	9	10686.16	4.56	0.01
139	3077.85	NA	1.77	NA	+	NA	NA	NA	+	NA	NA	NA	8	10686.21	4.61	0.01
46	2459.91	-10.33	NA	+	+	NA	+	NA	NA	NA	NA	NA	9	10686.23	4.63	0.01
32	2962.02	-144.11	4.91	+	+	+	NA	NA	NA	NA	NA	NA	10	10686.25	4.65	0.01
62	2850.06	-83.97	NA	+	+	+	+	NA	NA	NA	NA	NA	10	10686.34	4.74	0.01
12	3396.82	-96.79	6.47	NA	+	NA	NA	NA	NA	NA	NA	NA	8	10686.46	4.87	0.01
42	3183.74	-5.93	NA	NA	+	NA	+	NA	NA	NA	NA	NA	8	10686.50	4.91	0.01
88	2317.61	-82.36	-0.66	+	NA	+	NA	+	NA	NA	NA	NA	10	10687.27	5.67	0.01
399	2083.93	NA	1.48	+	+	NA	NA	NA	+	+	NA	NA	10	10687.76	6.16	0.00
144	2556.01	-83.16	7.33	+	+	NA	NA	NA	+	NA	NA	NA	10	10687.86	6.26	0.00
336	2603.01	-69.62	-1.64	+	+	NA	NA	+	NA	+	NA	NA	11	10687.93	6.33	0.00
272	2402.36	-103.28	6.76	+	+	NA	NA	NA	NA	+	NA	NA	10	10687.94	6.34	0.00
302	2200.22	-10.86	NA	+	+	NA	+	NA	NA	+	NA	NA	10	10688.02	6.42	0.00
208	2774.24	-62.06	-1.25	+	+	NA	NA	+	+	NA	NA	NA	11	10688.08	6.48	0.00
140	3277.24	-76.59	7.11	NA	+	NA	NA	NA	+	NA	NA	NA	9	10688.12	6.53	0.00
288	2736.59	-144.51	4.96	+	+	+	NA	NA	NA	+	NA	NA	11	10688.13	6.53	0.00
96	2837.40	-75.86	-1.18	+	+	+	NA	+	NA	NA	NA	NA	11	10688.13	6.53	0.00
112	2829.22	-70.16	-1.77	+	+	NA	+	+	NA	NA	NA	NA	11	10688.13	6.53	0.00
48	2587.03	-81.82	5.99	+	+	NA	+	NA	NA	NA	NA	NA	10	10688.16	6.56	0.00
318	2621.37	-83.21	NA	+	+	+	+	NA	NA	+	NA	NA	11	10688.22	6.62	0.00
64	2947.96	-140.37	4.79	+	+	+	+	NA	NA	NA	NA	NA	11	10688.33	6.73	0.00
44	3305.88	-72.64	5.59	NA	+	NA	+	NA	NA	NA	NA	NA	9	10688.44	6.85	0.00
176	2923.79	-233.40	18.79	+	+	NA	+	NA	+	NA	NA	NA	11	10689.01	7.41	0.00
172	3639.52	-221.07	18.14	NA	+	NA	+	NA	+	NA	NA	NA	10	10689.34	7.74	0.00
192	3179.11	-255.78	15.22	+	+	+	+	NA	+	NA	NA	NA	12	10689.64	8.04	0.00
400	2294.19	-84.08	7.37	+	+	NA	NA	NA	+	+	NA	NA	11	10689.65	8.06	0.00
240	3030.93	-177.72	8.27	+	+	NA	+	+	+	NA	NA	NA	12	10689.66	8.06	0.00
304	2327.23	-82.46	6.00	+	+	NA	+	NA	NA	+	NA	NA	11	10689.96	8.36	0.00
464	2550.93	-63.45	-1.03	+	+	NA	NA	+	+	+	NA	NA	12	10689.97	8.37	0.00
352	2621.00	-77.95	-0.97	+	+	+	NA	+	NA	+	NA	NA	12	10690.02	8.42	0.00
1487	2409.13	NA	-5.98	+	+	NA	NA	+	+	+	NA	+	12	10690.05	8.45	0.00
416	2657.02	-131.29	5.40	+	+	+	NA	NA	+	+	NA	NA	12	10690.10	8.50	0.00
830	2700.64	-99.13	NA	+	+	+	+	NA	NA	+	+	NA	12	10690.16	8.57	0.00
224	2800.86	-75.26	-0.12	+	+	+	NA	+	+	NA	NA	NA	12	10690.17	8.57	0.00
320	2719.14	-139.99	4.82	+	+	+	+	NA	NA	+	NA	NA	12	10690.22	8.62	0.00
128	2844.46	-77.45	-1.16	+	+	+	+	+	NA	NA	NA	NA	12	10690.22	8.62	0.00
432	2660.91	-234.93	18.87	+	+	NA	+	NA	+	+	NA	NA	12	10690.81	9.21	0.00
448	2944.45	-256.66	15.37	+	+	+	+	NA	+	+	NA	NA	13	10691.52	9.92	0.00
496	2804.29	-180.56	8.61	+	+	NA	+	+	+	+	NA	NA	13	10691.54	9.94	0.00
256	3116.41	-219.39	11.82	+	+	+	+	+	+	NA	NA	NA	13	10691.70	10.11	0.00
480	2578.43	-77.34	0.16	+	+	+	NA	+	+	+	NA	NA	13	10692.06	10.46	0.00
1488	2553.53	-60.17	-1.56	+	+	NA	NA	+	+	+	NA	+	13	10692.06	10.47	0.00
384	2625.20	-78.86	-0.96	+	+	+	+	+	NA	+	NA	NA	13	10692.12	10.52	0.00
832	2769.92	-142.87	3.87	+	+	+	+	NA	NA	+	+	NA	13	10692.21	10.61	0.00
960	3023.82	-266.84	14.70	+	+	+	+	NA	+	+	+	NA	14	10693.44	11.84	0.00
512	2890.49	-223.42	12.26	+	+	+	+	+	+	+	NA	NA	14	10693.59	12.00	0.00
1520	2821.56	-177.80	7.77	+	+	NA	+	+	+	+	NA	+	14	10693.61	12.01	0.00
1504	2591.04	-77.35	-0.18	+	+	+	NA	+	+	+	NA	+	14	10694.16	12.56	0.00
896	2674.27	-88.89	-0.90	+	+	+	+	+	NA	+	+	NA	14	10694.18	12.58	0.00
1024	3003.27	-255.65	13.71	+	+	+	+	+	+	+	+	NA	15	10695.55	13.95	0.00
1536	2948.94	-235.59	12.39	+	+	+	+	+	+	+	NA	+	15	10695.63	14.03	0.00
2048	3062.76	-288.10	16.60	+	+	+	+	+	+	+	+	+	16	10697.58	15.98	0.00
463	226.54	NA	1.17	+	+	NA	NA	+	+	+	NA	NA	11	10713.08	31.49	0.00
160	1017.47	-142.82	11.59	+	+	+	NA	NA	+	NA	NA	NA	11	10713.42	31.82	0.00

368	570.09	-119.22	8.43	+	+	NA	+	+	NA	+	NA	NA	12	10714.88	33.28	0.00

PROTOSTRONGYLIDS ABUNDANCE

	I	AL	P	S	AL:P	AL:S	P:S	AL:P:S	NA	NA	NA	NA	df	AIC	delta	weight
40	-0.40	0.35	+	+	NA	NA	+	NA	NA	NA	NA	NA	9	2925.97	0.00	0.19
6	-0.51	0.33	NA	+	NA	NA	NA	NA	NA	NA	NA	NA	7	2926.31	0.34	0.16
2	-0.26	0.33	NA	NA	NA	NA	NA	NA	NA	NA	NA	NA	6	2926.79	0.82	0.12
56	-0.15	0.31	+	+	NA	+	+	NA	NA	NA	NA	NA	10	2927.21	1.24	0.10
22	-0.31	0.30	NA	+	NA	+	NA	NA	NA	NA	NA	NA	8	2927.85	1.88	0.07
48	-0.55	0.37	+	+	+	NA	+	NA	NA	NA	NA	NA	10	2927.92	1.95	0.07
8	-0.55	0.33	+	+	NA	NA	NA	NA	NA	NA	NA	NA	8	2928.18	2.21	0.06
4	-0.28	0.33	+	NA	NA	NA	NA	NA	NA	NA	NA	NA	7	2928.78	2.81	0.05
128	-0.61	0.39	+	+	+	+	+	+	NA	NA	NA	NA	12	2929.20	3.22	0.04
64	-0.24	0.32	+	+	+	+	+	NA	NA	NA	NA	NA	11	2929.27	3.29	0.04
24	-0.34	0.29	+	+	NA	+	NA	NA	NA	NA	NA	NA	9	2929.65	3.68	0.03
16	-0.83	0.37	+	+	+	NA	NA	NA	NA	NA	NA	NA	9	2929.76	3.79	0.03
12	-0.65	0.39	+	NA	+	NA	NA	NA	NA	NA	NA	NA	8	2930.09	4.12	0.02
32	-0.60	0.33	+	+	+	+	NA	NA	NA	NA	NA	NA	10	2931.40	5.43	0.01
5	1.77	NA	NA	+	NA	NA	NA	NA	NA	NA	NA	NA	6	2932.73	6.76	0.01
1	2.00	NA	NA	NA	NA	NA	NA	NA	NA	NA	NA	NA	5	2933.19	7.21	0.01
39	1.91	NA	+	+	NA	NA	+	NA	NA	NA	NA	NA	8	2933.19	7.22	0.01
7	1.64	NA	+	+	NA	NA	NA	NA	NA	NA	NA	NA	7	2934.21	8.24	0.00
3	1.92	NA	+	NA	NA	NA	NA	NA	NA	NA	NA	NA	6	2934.92	8.95	0.00
	I	AF	P	S	AF:P	AF:S	P:S	AF:P:S	NA	NA	NA	NA	df	AIC	delta	weight
128	0.23	+	+	+	+	+	+	+	NA	NA	NA	NA	46	2926.74	0.00	0.65
40	0.02	+	+	+	NA	NA	+	NA	NA	NA	NA	NA	18	2930.90	4.16	0.08
6	-0.23	+	NA	+	NA	NA	NA	NA	NA	NA	NA	NA	16	2931.27	4.53	0.07
5	1.77	NA	NA	+	NA	NA	NA	NA	NA	NA	NA	NA	6	2932.73	5.99	0.03
2	0.13	+	NA	NA	NA	NA	NA	NA	NA	NA	NA	NA	15	2933.12	6.38	0.03
1	2.00	NA	NA	NA	NA	NA	NA	NA	NA	NA	NA	NA	5	2933.19	6.44	0.03
39	1.91	NA	+	+	NA	NA	+	NA	NA	NA	NA	NA	8	2933.19	6.45	0.03
8	-0.27	+	+	+	NA	NA	NA	NA	NA	NA	NA	NA	17	2933.37	6.62	0.02
7	1.64	NA	+	+	NA	NA	NA	NA	NA	NA	NA	NA	7	2934.21	7.47	0.02
3	1.92	NA	+	NA	NA	NA	NA	NA	NA	NA	NA	NA	6	2934.92	8.18	0.01
48	0.03	+	+	+	+	NA	+	NA	NA	NA	NA	NA	28	2934.96	8.22	0.01
4	0.13	+	+	NA	NA	NA	NA	NA	NA	NA	NA	NA	16	2935.26	8.51	0.01
16	-0.21	+	+	+	+	NA	NA	NA	NA	NA	NA	NA	27	2935.53	8.79	0.01
12	0.18	+	+	NA	+	NA	NA	NA	NA	NA	NA	NA	26	2937.39	10.65	0.00
56	0.41	+	+	+	NA	+	+	NA	NA	NA	NA	NA	28	2940.74	14.00	0.00
22	0.18	+	NA	+	NA	+	NA	NA	NA	NA	NA	NA	26	2940.81	14.07	0.00
64	0.42	+	+	+	+	+	+	NA	NA	NA	NA	NA	38	2941.20	14.46	0.00
32	0.17	+	+	+	+	+	NA	NA	NA	NA	NA	NA	37	2941.46	14.72	0.00
24	0.13	+	+	+	NA	+	NA	NA	NA	NA	NA	NA	27	2942.92	16.17	0.00
	I	AT	P	S	AT:P	AT:S	P:S	AT:P:S	NA	NA	NA	NA	df	AIC	delta	weight
128	-24.67	2.76	+	+	+	+	+	+	NA	NA	NA	NA	12	2892.40	0.00	1.00
48	-20.27	2.29	+	+	+	NA	+	NA	NA	NA	NA	NA	10	2920.04	27.64	0.00
16	-21.00	2.34	+	+	+	NA	NA	NA	NA	NA	NA	NA	9	2920.75	28.35	0.00
64	-22.30	2.50	+	+	+	+	+	NA	NA	NA	NA	NA	11	2921.23	28.83	0.00
32	-23.42	2.60	+	+	+	+	NA	NA	NA	NA	NA	NA	10	2921.43	29.03	0.00
12	-19.98	2.26	+	NA	+	NA	NA	NA	NA	NA	NA	NA	8	2921.50	29.10	0.00
40	-14.02	1.61	+	+	NA	NA	+	NA	NA	NA	NA	NA	9	2924.96	32.56	0.00
6	-13.97	1.58	NA	+	NA	NA	NA	NA	NA	NA	NA	NA	7	2925.38	32.98	0.00
2	-12.84	1.49	NA	NA	NA	NA	NA	NA	NA	NA	NA	NA	6	2926.30	33.89	0.00
56	-14.06	1.62	+	+	NA	+	+	NA	NA	NA	NA	NA	10	2927.04	34.64	0.00
8	-14.13	1.59	+	+	NA	NA	NA	NA	NA	NA	NA	NA	8	2927.28	34.88	0.00
22	-14.30	1.61	NA	+	NA	+	NA	NA	NA	NA	NA	NA	8	2927.39	34.99	0.00
4	-12.90	1.49	+	NA	NA	NA	NA	NA	NA	NA	NA	NA	7	2928.31	35.91	0.00
24	-14.41	1.62	+	+	NA	+	NA	NA	NA	NA	NA	NA	9	2929.31	36.91	0.00
5	1.77	NA	NA	+	NA	NA	NA	NA	NA	NA	NA	NA	6	2932.73	40.33	0.00
1	2.00	NA	NA	NA	NA	NA	NA	NA	NA	NA	NA	NA	5	2933.19	40.78	0.00
39	1.91	NA	+	+	NA	NA	+	NA	NA	NA	NA	NA	8	2933.19	40.79	0.00
7	1.64	NA	+	+	NA	NA	NA	NA	NA	NA	NA	NA	7	2934.21	41.81	0.00
3	1.92	NA	+	NA	NA	NA	NA	NA	NA	NA	NA	NA	6	2934.92	42.52	0.00
	I	AL	I(AL^2)	P	S	AL:P	AL:S	I(AL^2):P	I(AL^2):S	P:S	AL:P:S	I(AL^2):P:S	df	AIC	delta	weight
271	0.17	NA	0.03	+	+	NA	NA	NA	NA	+	NA	NA	9	2924.60	0.00	0.06
11	0.02	NA	0.03	NA	+	NA	NA	NA	NA	NA	NA	NA	7	2924.99	0.39	0.05
352	1.64	-0.84	0.10	+	+	+	NA	+	NA	+	NA	NA	12	2925.52	0.92	0.04
1488	0.58	-0.50	0.08	+	+	NA	NA	+	+	+	NA	+	13	2925.69	1.09	0.03
3	0.32	NA	0.03	NA	NA	NA	NA	NA	NA	NA	NA	NA	6	2925.90	1.30	0.03
1487	-0.23	NA	0.04	+	+	NA	NA	+	+	+	NA	+	12	2925.92	1.32	0.03
399	0.29	NA	0.03	+	+	NA	NA	NA	+	+	NA	NA	10	2925.97	1.37	0.03
270	-0.40	0.35	NA	+	+	NA	NA	NA	NA	+	NA	NA	9	2925.97	1.37	0.03
335	-0.41	NA	0.04	+	+	NA	NA	+	NA	+	NA	NA	10	2926.05	1.45	0.03
512	2.82	-1.34	0.14	+	+	+	+	+	+	+	NA	NA	14	2926.28	1.68	0.02
10	-0.51	0.33	NA	NA	+	NA	NA	NA	NA	NA	NA	NA	7	2926.31	1.71	0.02
1504	1.31	-0.79	0.10	+	+	+	NA	+	+	+	NA	+	14	2926.46	1.86	0.02
1520	1.23	-0.76	0.10	+	+	NA	+	+	+	+	NA	+	14	2926.50	1.90	0.02
96	1.46	-0.90	0.10	+	+	+	NA	+	NA	NA	NA	NA	11	2926.57	1.97	0.02
1536	2.33	-1.20	0.13	+	+	+	+	+	+	+	NA	+	15	2926.57	1.97	0.02
139	0.11	NA	0.03	NA	+	NA	NA	NA	+	NA	NA	NA	8	2926.64	2.04	0.02
272	0.31	-0.07	0.04	+	+	NA	NA	NA	NA	+	NA	NA	10	2926.65	2.05	0.02
336	0.49	-0.37	0.06	+	+	NA	NA	+	NA	+	NA	NA	11	2926.65	2.05	0.02
2	-0.26	0.33	NA	NA	NA	NA	NA	NA	NA	NA	NA	NA	6	2926.79	2.19	0.02
15	-0.03	NA	0.03	+	+	NA	NA	NA	NA	NA	NA	NA	8	2926.85	2.25	0.02
256	2.65	-1.40	0.15	+	+	+	+	+	+	NA	NA	NA	13	2926.95	2.35	0.02
12	0.20	-0.08	0.04	NA	+	NA	NA	NA	NA	NA	NA	NA	8	2927.00	2.40	0.02
1024	2.31	-1.18	0.13	+	+	+	+	+	+	+	+	NA	15	2927.20	2.60	0.02
896	1.34	-0.77	0.10	+	+	+	+	+	NA	+	+	NA	14	2927.21	2.61	0.02
302	-0.15	0.31	NA	+	+	NA	+	NA	NA	+	NA	NA	10	2927.21	2.61	0.02
88	1.63	-0.82	0.10	+	NA	+	NA	+	NA	NA	NA	NA	10	2927.47	2.87	0.01
384	1.81	-0.88	0.10	+	+	+	+	+	NA	+	NA	NA	13	2927.47	2.87	0.01
304	0.73	-0.18	0.04	+	+	NA	+	NA	NA	+	NA	NA	11	2927.49	2.89	0.01
480	1.63	-0.84	0.10	+	+	+	NA	+	+	+	NA	NA	13	2927.62	3.02	0.01
79	-0.76	NA	0.04	+	+	NA	NA	+	NA	NA	NA	NA	9	2927.64	3.04	0.01
832	0.38	-0.36	0.07	+	+	+	+	NA	NA	+	+	NA	13	2927.70	3.10	0.01
2048	2.79	-1.38	0.15	+	+	+	+	+	+	+	+	+	16	2927.84	3.24	0.01
42	-0.31	0.30	NA	NA	+	NA	+	NA	NA	NA	NA	NA	8	2927.85	3.25	0.01
7	0.30	NA	0.03	+	NA	NA	NA	NA	NA	NA	NA	NA	7	2927.89	3.29	0.01
286	-0.55	0.37	NA	+	+	+	NA	NA	NA	+	NA	NA	10	2927.92	3.32	0.01
4	0.33	0.00	0.03	NA	NA	NA	NA	NA	NA	NA	NA	NA	7	2927.96	3.36	0.01
400	0.52	-0.11	0.04	+	+	NA	NA	NA	+	+	NA	NA	11	2927.98	3.37	0.01
71	-0.11	NA	0.04	+	NA	NA	NA	+	NA	NA	NA	NA	8	2928.11	3.51	0.01
463	-0.44	NA	0.04	+	+	NA	NA	+	+	+	NA	NA	11	2928.12	3.51	0.01
14	-0.55	0.33	NA	+	+	NA	NA	NA	NA	NA	NA	NA	8	2928.18	3.58	0.01
72	0.52	-0.38	0.06	+	NA	NA	NA	+	NA	NA	NA	NA	9	2928.23	3.62	0.01
44	0.55	-0.18	0.04	NA	+	NA	+	NA	NA	NA	NA	NA	9	2928.23	3.63	0.01
496	1.44	-0.76	0.09	+	+	NA	+	+	+	+	NA	NA	13	2928.27	3.67	0.01
288	0.35	-0.28	0.05	+	+	+	NA	NA	NA	+	NA	NA	11	2928.40	3.80	0.01
143	0.06	NA	0.03	+	+	NA	NA	NA	+	NA	NA	NA	9	2928.45	3.85	0.01
224	1.38	-0.88	0.11	+	+	+	NA	+	+	NA	NA	NA	12	2928.60	4.00	0.01
140	0.37	-0.11	0.04	NA	+	NA	NA	NA	+	NA	NA	NA	9	2928.62	4.02	0.01
128	1.54	-0.92	0.11	+	+	+	+	+	NA	NA	NA	NA	12	2928.63	4.02	0.01
432	1.22	-0.38	0.06	+	+	NA	+	NA	+	+	NA	NA	12	2928.77	4.17	0.01
6	-0.28	0.33	NA	+	NA	NA	NA	NA	NA	NA	NA	NA	7	2928.78	4.18	0.01
960	0.86	-0.56	0.08	+	+	+	+	NA	+	+	+	NA	14	2928.83	4.23	0.01
16	0.16	-0.09	0.04	+	+	NA	NA	NA	NA	NA	NA	NA	9	2928.86	4.26	0.01
368	0.56	-0.21	0.05	+	+	NA	+	+	NA	+	NA	NA	12	2929.14	4.54	0.01
830	-0.61	0.39	NA	+	+	+	+	NA	NA	+	+	NA	12	2929.20	4.59	0.01
240	1.20	-0.80	0.10	+	+	NA	+	+	+	NA	NA	NA	12	2929.23	4.63	0.01
318	-0.24	0.32	NA	+	+	+	+	NA	NA	+	NA	NA	11	2929.27	4.66	0.01
80	-0.04	-0.17	0.05	+	+	NA	NA	+	NA	NA	NA	NA	10	2929.38	4.78	0.01
172	1.06	-0.38	0.06	NA	+	NA	+	NA	+	NA	NA	NA	10	2929.44	4.83	0.01
207	-0.82	NA	0.04	+	+	NA	NA	+	+	NA	NA	NA	10	2929.56	4.96	0.00
464	0.36	-0.14	0.04	+	+	NA	NA	+	+	+	NA	NA	12	2929.60	5.00	0.00
46	-0.34	0.29	NA	+	+	NA	+	NA	NA	NA	NA	NA	9	2929.65	5.05	0.00
30	-0.83	0.37	NA	+	+	+	NA	NA	NA	NA	NA	NA	9	2929.76	5.16	0.00
8	0.31	0.00	0.03	+	NA	NA	NA	NA	NA	NA	NA	NA	8	2929.96	5.36	0.00
48	0.52	-0.19	0.04	+	+	NA	+	NA	NA	NA	NA	NA	10	2929.99	5.39	0.00
22	-0.65	0.39	NA	+	NA	+	NA	NA	NA	NA	NA	NA	8	2930.09	5.49	0.00
320	0.66	-0.36	0.06	+	+	+	+	NA	NA	+	NA	NA	12	2930.12	5.52	0.00
32	-0.11	-0.08	0.04	+	+	+	NA	NA	NA	NA	NA	NA	10	2930.15	5.55	0.00
448	1.29	-0.64	0.08	+	+	+	+	NA	+	+	NA	NA	13	2930.33	5.72	0.00
144	0.33	-0.12	0.04	+	+	NA	NA	NA	+	NA	NA	NA	10	2930.42	5.82	0.00
416	0.45	-0.30	0.05	+	+	+	NA	NA	+	+	NA	NA	12	2930.43	5.83	0.00
112	0.27	-0.23	0.05	+	+	NA	+	+	NA	NA	NA	NA	11	2930.89	6.29	0.00
24	-0.01	0.00	0.03	+	NA	+	NA	NA	NA	NA	NA	NA	9	2930.94	6.34	0.00
176	1.04	-0.40	0.06	+	+	NA	+	NA	+	NA	NA	NA	11	2931.16	6.56	0.00
208	0.09	-0.18	0.05	+	+	NA	NA	+	+	NA	NA	NA	11	2931.26	6.66	0.00
62	-0.60	0.33	NA	+	+	+	+	NA	NA	NA	NA	NA	10	2931.40	6.80	0.00
64	0.27	-0.36	0.06	+	+	+	+	NA	NA	NA	NA	NA	11	2931.96	7.36	0.00
160	0.09	-0.30	0.06	+	+	+	NA	NA	+	NA	NA	NA	11	2932.14	7.54	0.00
192	0.78	-0.41	0.06	+	+	+	+	NA	+	NA	NA	NA	12	2932.45	7.85	0.00
9	1.77	NA	NA	NA	+	NA	NA	NA	NA	NA	NA	NA	6	2932.73	8.13	0.00
1	2.00	NA	NA	NA	NA	NA	NA	NA	NA	NA	NA	NA	5	2933.19	8.59	0.00
269	1.91	NA	NA	+	+	NA	NA	NA	NA	+	NA	NA	8	2933.19	8.59	0.00
13	1.64	NA	NA	+	+	NA	NA	NA	NA	NA	NA	NA	7	2934.21	9.61	0.00
5	1.92	NA	NA	+	NA	NA	NA	NA	NA	NA	NA	NA	6	2934.92	10.32	0.00

TRICHURIS SP. ABUNDANCE

	I	AL	P	S	AL:P	AL:S	P:S	AL:P:S	NA	NA	NA	NA	df	AIC	delta	weight
128	-9.33	2.87	+	+	+	+	+	+	NA	NA	NA	NA	12	6920.35	0.00	0.65
64	7.41	-0.22	+	+	+	+	+	NA	NA	NA	NA	NA	11	6922.09	1.73	0.27
48	-20.43	4.85	+	+	+	NA	+	NA	NA	NA	NA	NA	10	6926.29	5.94	0.03
32	-13.49	0.74	+	+	+	+	NA	NA	NA	NA	NA	NA	10	6927.50	7.15	0.02
56	-31.77	6.89	+	+	NA	+	+	NA	NA	NA	NA	NA	10	6929.15	8.79	0.01
16	-36.07	5.12	+	+	+	NA	NA	NA	NA	NA	NA	NA	9	6930.19	9.84	0.00
40	-51.84	10.54	+	+	NA	NA	+	NA	NA	NA	NA	NA	9	6931.50	11.15	0.00
24	-42.90	6.63	+	+	NA	+	NA	NA	NA	NA	NA	NA	9	6931.71	11.35	0.00
8	-60.23	9.96	+	+	NA	NA	NA	NA	NA	NA	NA	NA	8	6933.26	12.91	0.00
12	-13.21	4.84	+	NA	+	NA	NA	NA	NA	NA	NA	NA	8	6938.45	18.09	0.00
4	-35.43	9.02	+	NA	NA	NA	NA	NA	NA	NA	NA	NA	7	6939.82	19.47	0.00
39	11.96	NA	+	+	NA	NA	+	NA	NA	NA	NA	NA	8	6941.67	21.32	0.00
7	1.89	NA	+	+	NA	NA	NA	NA	NA	NA	NA	NA	7	6942.40	22.04	0.00
3	18.50	NA	+	NA	NA	NA	NA	NA	NA	NA	NA	NA	6	6947.08	26.72	0.00
6	-11.34	9.44	NA	+	NA	NA	NA	NA	NA	NA	NA	NA	7	6968.65	48.30	0.00
22	8.49	5.99	NA	+	NA	+	NA	NA	NA	NA	NA	NA	8	6968.66	48.31	0.00
2	7.34	8.75	NA	NA	NA	NA	NA	NA	NA	NA	NA	NA	6	6971.55	51.19	0.00
5	44.40	NA	NA	+	NA	NA	NA	NA	NA	NA	NA	NA	6	6973.10	52.74	0.00
1	58.44	NA	NA	NA	NA	NA	NA	NA	NA	NA	NA	NA	5	6975.46	55.10	0.00
	I	AF	P	S	AF:P	AF:S	P:S	AF:P:S	NA	NA	NA	NA	df	AIC	delta	weight
128	5.75	+	+	+	+	+	+	+	NA	NA	NA	NA	46	6935.50	0.00	0.65
40	-27.14	+	+	+	NA	NA	+	NA	NA	NA	NA	NA	18	6939.44	3.94	0.09
48	-1.60	+	+	+	+	NA	+	NA	NA	NA	NA	NA	28	6940.44	4.93	0.06
8	-36.18	+	+	+	NA	NA	NA	NA	NA	NA	NA	NA	17	6940.78	5.28	0.05
64	19.01	+	+	+	+	+	+	NA	NA	NA	NA	NA	38	6941.06	5.56	0.04
56	-6.78	+	+	+	NA	+	+	NA	NA	NA	NA	NA	28	6941.42	5.92	0.03
39	11.96	NA	+	+	NA	NA	+	NA	NA	NA	NA	NA	8	6941.67	6.17	0.03
7	1.89	NA	+	+	NA	NA	NA	NA	NA	NA	NA	NA	7	6942.40	6.89	0.02
16	-13.60	+	+	+	+	NA	NA	NA	NA	NA	NA	NA	27	6943.06	7.56	0.01
24	-17.73	+	+	+	NA	+	NA	NA	NA	NA	NA	NA	27	6943.56	8.06	0.01
32	3.98	+	+	+	+	+	NA	NA	NA	NA	NA	NA	37	6945.39	9.89	0.00
3	18.50	NA	+	NA	NA	NA	NA	NA	NA	NA	NA	NA	6	6947.08	11.57	0.00
4	-15.45	+	+	NA	NA	NA	NA	NA	NA	NA	NA	NA	16	6947.45	11.94	0.00
12	5.13	+	+	NA	+	NA	NA	NA	NA	NA	NA	NA	26	6950.19	14.69	0.00
6	3.88	+	NA	+	NA	NA	NA	NA	NA	NA	NA	NA	16	6972.81	37.31	0.00
5	44.40	NA	NA	+	NA	NA	NA	NA	NA	NA	NA	NA	6	6973.10	37.59	0.00
22	21.55	+	NA	+	NA	+	NA	NA	NA	NA	NA	NA	26	6975.45	39.95	0.00
1	58.44	NA	NA	NA	NA	NA	NA	NA	NA	NA	NA	NA	5	6975.46	39.95	0.00
2	20.42	+	NA	NA	NA	NA	NA	NA	NA	NA	NA	NA	15	6975.87	40.37	0.00
	I	AT	P	S	AT:P	AT:S	P:S	AT:P:S	NA	NA	NA	NA	df	AIC	delta	weight
128	-20.85	4.19	+	+	+	+	+	+	NA	NA	NA	NA	12	6911.47	0.00	0.97
64	18.73	-1.94	+	+	+	+	+	NA	NA	NA	NA	NA	11	6918.79	7.32	0.03
32	-8.36	-0.14	+	+	+	+	NA	NA	NA	NA	NA	NA	10	6924.02	12.54	0.00
48	-41.64	7.32	+	+	+	NA	+	NA	NA	NA	NA	NA	10	6926.69	15.22	0.00
56	-55.63	9.46	+	+	NA	+	+	NA	NA	NA	NA	NA	10	6927.23	15.76	0.00
24	-66.32	9.30	+	+	NA	+	NA	NA	NA	NA	NA	NA	9	6929.22	17.74	0.00
16	-59.39	8.01	+	+	+	NA	NA	NA	NA	NA	NA	NA	9	6929.90	18.42	0.00
40	-92.48	15.11	+	+	NA	NA	+	NA	NA	NA	NA	NA	9	6931.45	19.97	0.00
8	-99.66	14.57	+	+	NA	NA	NA	NA	NA	NA	NA	NA	8	6932.64	21.17	0.00
12	-32.43	7.11	+	NA	+	NA	NA	NA	NA	NA	NA	NA	8	6938.25	26.78	0.00
4	-69.73	13.02	+	NA	NA	NA	NA	NA	NA	NA	NA	NA	7	6939.65	28.18	0.00
39	11.96	NA	+	+	NA	NA	+	NA	NA	NA	NA	NA	8	6941.67	30.20	0.00
7	1.89	NA	+	+	NA	NA	NA	NA	NA	NA	NA	NA	7	6942.40	30.92	0.00
3	18.50	NA	+	NA	NA	NA	NA	NA	NA	NA	NA	NA	6	6947.08	35.60	0.00
22	-12.86	8.21	NA	+	NA	+	NA	NA	NA	NA	NA	NA	8	6966.46	54.98	0.00
6	-56.26	14.76	NA	+	NA	NA	NA	NA	NA	NA	NA	NA	7	6968.20	56.73	0.00
2	-31.60	13.33	NA	NA	NA	NA	NA	NA	NA	NA	NA	NA	6	6971.35	59.87	0.00
5	44.40	NA	NA	+	NA	NA	NA	NA	NA	NA	NA	NA	6	6973.10	61.62	0.00
1	58.44	NA	NA	NA	NA	NA	NA	NA	NA	NA	NA	NA	5	6975.46	63.98	0.00
	I	AL	I(AL^2)	P	S	AL:P	AL:S	I(AL^2):P	I(AL^2):S	P:S	AL:P:S	I(AL^2):P:S	df	AIC	delta	weight
1487	-2.74	NA	0.23	+	+	NA	NA	+	+	+	NA	+	12	6914.86	0.00	0.24
1520	-36.13	13.03	-0.73	+	+	NA	+	+	+	+	NA	+	14	6915.11	0.25	0.21
2048	7.93	-4.14	0.54	+	+	+	+	+	+	+	+	+	16	6915.38	0.51	0.19
1488	4.21	-2.70	0.43	+	+	NA	NA	+	+	+	NA	+	13	6916.90	2.04	0.09
1536	-27.52	9.69	-0.49	+	+	+	+	+	+	+	NA	+	15	6917.13	2.26	0.08
960	-24.44	9.25	-0.50	+	+	+	+	NA	+	+	+	NA	14	6918.00	3.14	0.05
1504	19.07	-8.46	0.86	+	+	+	NA	+	+	+	NA	+	14	6918.58	3.72	0.04
1024	-30.58	11.77	-0.70	+	+	+	+	+	+	+	+	NA	15	6920.06	5.19	0.02
830	-9.33	2.87	NA	+	+	+	+	NA	NA	+	+	NA	12	6920.35	5.49	0.02
463	8.93	NA	-0.07	+	+	NA	NA	+	+	+	NA	NA	11	6920.92	6.06	0.01
832	17.18	-8.05	0.85	+	+	+	+	NA	NA	+	+	NA	13	6921.44	6.58	0.01
416	21.13	-4.65	0.27	+	+	+	NA	NA	+	+	NA	NA	12	6921.51	6.65	0.01
318	7.41	-0.22	NA	+	+	+	+	NA	NA	+	NA	NA	11	6922.09	7.23	0.01
448	-3.05	5.11	-0.48	+	+	+	+	NA	+	+	NA	NA	13	6922.14	7.28	0.01
464	3.41	2.10	-0.22	+	+	NA	NA	+	+	+	NA	NA	12	6922.97	8.11	0.00
480	34.09	-10.38	0.74	+	+	+	NA	+	+	+	NA	NA	13	6923.15	8.29	0.00
496	-22.17	12.52	-1.03	+	+	NA	+	+	+	+	NA	NA	13	6923.46	8.60	0.00
896	22.88	-10.39	1.03	+	+	+	+	+	NA	+	+	NA	14	6923.48	8.61	0.00
320	27.92	-8.49	0.63	+	+	+	+	NA	NA	+	NA	NA	12	6923.62	8.75	0.00
512	8.54	0.03	-0.06	+	+	+	+	+	+	+	NA	NA	14	6923.97	9.10	0.00
384	43.37	-15.25	1.18	+	+	+	+	+	NA	+	NA	NA	13	6925.06	10.20	0.00
368	10.45	-1.97	0.17	+	+	NA	+	+	NA	+	NA	NA	12	6925.17	10.31	0.00
286	-20.43	4.85	NA	+	+	+	NA	NA	NA	+	NA	NA	10	6926.29	11.43	0.00
207	-10.45	NA	0.02	+	+	NA	NA	+	+	NA	NA	NA	10	6926.47	11.61	0.00
160	2.70	-4.87	0.36	+	+	+	NA	NA	+	NA	NA	NA	11	6927.20	12.33	0.00
62	-13.49	0.74	NA	+	+	+	+	NA	NA	NA	NA	NA	10	6927.50	12.64	0.00
399	-13.12	NA	0.48	+	+	NA	NA	NA	+	+	NA	NA	10	6927.89	13.03	0.00
192	-21.54	4.89	-0.38	+	+	+	+	NA	+	NA	NA	NA	12	6927.91	13.05	0.00
288	-16.92	3.35	0.12	+	+	+	NA	NA	NA	+	NA	NA	11	6928.35	13.49	0.00
224	20.39	-12.49	0.99	+	+	+	NA	+	+	NA	NA	NA	12	6928.49	13.63	0.00
208	-12.59	0.80	-0.03	+	+	NA	NA	+	+	NA	NA	NA	11	6928.55	13.69	0.00
335	-10.15	NA	0.41	+	+	NA	NA	+	NA	+	NA	NA	10	6928.61	13.75	0.00
64	8.92	-8.24	0.68	+	+	+	+	NA	NA	NA	NA	NA	11	6928.93	14.06	0.00
302	-31.77	6.89	NA	+	+	NA	+	NA	NA	+	NA	NA	10	6929.15	14.28	0.00
240	-38.12	11.20	-0.84	+	+	NA	+	+	+	NA	NA	NA	12	6929.16	14.29	0.00
352	5.48	-6.13	0.87	+	+	+	NA	+	NA	+	NA	NA	12	6929.30	14.43	0.00
256	-4.54	-2.40	0.21	+	+	+	+	+	+	NA	NA	NA	13	6929.46	14.60	0.00
400	-27.52	5.63	0.07	+	+	NA	NA	NA	+	+	NA	NA	11	6929.70	14.83	0.00
336	-33.05	9.14	-0.28	+	+	NA	NA	+	NA	+	NA	NA	11	6929.93	15.07	0.00
128	28.81	-16.72	1.37	+	+	+	+	+	NA	NA	NA	NA	12	6930.00	15.14	0.00
30	-36.07	5.12	NA	+	+	+	NA	NA	NA	NA	NA	NA	9	6930.19	15.33	0.00
143	-24.98	NA	0.47	+	+	NA	NA	NA	+	NA	NA	NA	9	6930.32	15.46	0.00
112	-6.15	-2.75	0.31	+	+	NA	+	+	NA	NA	NA	NA	11	6930.33	15.47	0.00
432	-47.83	13.62	-0.53	+	+	NA	+	NA	+	+	NA	NA	12	6930.90	16.04	0.00
304	-20.25	2.12	0.37	+	+	NA	+	NA	NA	+	NA	NA	11	6931.04	16.17	0.00
270	-51.84	10.54	NA	+	+	NA	NA	NA	NA	+	NA	NA	9	6931.50	16.64	0.00
46	-42.90	6.63	NA	+	+	NA	+	NA	NA	NA	NA	NA	9	6931.71	16.84	0.00
79	-24.84	NA	0.44	+	+	NA	NA	+	NA	NA	NA	NA	9	6931.98	17.12	0.00
32	-29.48	2.35	0.22	+	+	+	NA	NA	NA	NA	NA	NA	10	6932.20	17.33	0.00
144	-35.27	3.98	0.18	+	+	NA	NA	NA	+	NA	NA	NA	10	6932.26	17.40	0.00
271	-25.82	NA	0.81	+	+	NA	NA	NA	NA	+	NA	NA	9	6932.58	17.72	0.00
96	-3.43	-8.47	1.08	+	+	+	NA	+	NA	NA	NA	NA	11	6932.78	17.92	0.00
14	-60.23	9.96	NA	+	+	NA	NA	NA	NA	NA	NA	NA	8	6933.26	18.40	0.00
176	-56.70	12.45	-0.46	+	+	NA	+	NA	+	NA	NA	NA	11	6933.38	18.52	0.00
48	-28.71	0.80	0.45	+	+	NA	+	NA	NA	NA	NA	NA	10	6933.49	18.63	0.00
80	-43.35	7.26	-0.11	+	+	NA	NA	+	NA	NA	NA	NA	10	6933.58	18.71	0.00
272	-52.67	10.90	-0.03	+	+	NA	NA	NA	NA	+	NA	NA	10	6933.58	18.72	0.00
15	-35.42	NA	0.77	+	+	NA	NA	NA	NA	NA	NA	NA	8	6934.00	19.14	0.00
16	-58.08	9.04	0.07	+	+	NA	NA	NA	NA	NA	NA	NA	9	6935.33	20.46	0.00
22	-13.21	4.84	NA	+	NA	+	NA	NA	NA	NA	NA	NA	8	6938.45	23.59	0.00
6	-35.43	9.02	NA	+	NA	NA	NA	NA	NA	NA	NA	NA	7	6939.82	24.96	0.00
71	-2.29	NA	0.41	+	NA	NA	NA	+	NA	NA	NA	NA	8	6940.16	25.29	0.00
24	-13.48	4.95	-0.01	+	NA	+	NA	NA	NA	NA	NA	NA	9	6940.52	25.65	0.00
7	-12.52	NA	0.69	+	NA	NA	NA	NA	NA	NA	NA	NA	7	6940.75	25.88	0.00
88	10.55	-4.98	0.78	+	NA	+	NA	+	NA	NA	NA	NA	10	6941.37	26.51	0.00
72	-25.42	9.00	-0.27	+	NA	NA	NA	+	NA	NA	NA	NA	9	6941.51	26.64	0.00
269	11.96	NA	NA	+	+	NA	NA	NA	NA	+	NA	NA	8	6941.67	26.81	0.00
8	-38.50	10.31	-0.11	+	NA	NA	NA	NA	NA	NA	NA	NA	8	6941.86	27.00	0.00
13	1.89	NA	NA	+	+	NA	NA	NA	NA	NA	NA	NA	7	6942.40	27.53	0.00
5	18.50	NA	NA	+	NA	NA	NA	NA	NA	NA	NA	NA	6	6947.08	32.22	0.00
139	27.00	NA	0.36	NA	+	NA	NA	NA	+	NA	NA	NA	8	6967.84	52.98	0.00
172	-36.70	26.26	-1.67	NA	+	NA	+	NA	+	NA	NA	NA	10	6968.36	53.49	0.00
10	-11.34	9.44	NA	NA	+	NA	NA	NA	NA	NA	NA	NA	7	6968.65	53.79	0.00
42	8.49	5.99	NA	NA	+	NA	+	NA	NA	NA	NA	NA	8	6968.66	53.80	0.00
140	1.34	10.76	-0.47	NA	+	NA	NA	NA	+	NA	NA	NA	9	6969.15	54.29	0.00
11	9.48	NA	0.77	NA	+	NA	NA	NA	NA	NA	NA	NA	7	6969.65	54.79	0.00
12	-18.91	13.31	-0.34	NA	+	NA	NA	NA	NA	NA	NA	NA	8	6970.62	55.75	0.00
44	3.50	8.37	-0.20	NA	+	NA	+	NA	NA	NA	NA	NA	9	6970.69	55.83	0.00
2	7.34	8.75	NA	NA	NA	NA	NA	NA	NA	NA	NA	NA	6	6971.55	56.68	0.00
3	27.55	NA	0.70	NA	NA	NA	NA	NA	NA	NA	NA	NA	6	6972.58	57.72	0.00
9	44.40	NA	NA	NA	+	NA	NA	NA	NA	NA	NA	NA	6	6973.10	58.23	0.00
4	-3.77	14.35	-0.49	NA	NA	NA	NA	NA	NA	NA	NA	NA	7	6973.40	58.54	0.00
1	58.44	NA	NA	NA	NA	NA	NA	NA	NA	NA	NA	NA	5	6975.46	60.59	0.00

GATTRO-INTESTINAL STRONGYLES ABUNDANCE

	I	AL	P	S	AL:P	AL:S	P:S	AL:P:S	NA	NA	NA	NA	df	AIC	delta	weight
6	2.25	4.62	NA	+	NA	NA	NA	NA	NA	NA	NA	NA	7	5983.54	0.00	0.19
16	6.01	3.03	+	+	+	NA	NA	NA	NA	NA	NA	NA	9	5984.04	0.50	0.15
8	-1.69	4.70	+	+	NA	NA	NA	NA	NA	NA	NA	NA	8	5984.90	1.37	0.09
48	2.10	3.03	+	+	+	NA	+	NA	NA	NA	NA	NA	10	5984.95	1.42	0.09
40	-5.56	4.52	+	+	NA	NA	+	NA	NA	NA	NA	NA	9	5985.29	1.75	0.08
2	11.39	4.26	NA	NA	NA	NA	NA	NA	NA	NA	NA	NA	6	5985.39	1.85	0.07
22	2.99	4.49	NA	+	NA	+	NA	NA	NA	NA	NA	NA	8	5985.58	2.04	0.07
32	8.41	2.56	+	+	+	+	NA	NA	NA	NA	NA	NA	10	5985.90	2.37	0.06
12	15.45	2.87	+	NA	+	NA	NA	NA	NA	NA	NA	NA	8	5986.83	3.29	0.04
64	4.31	2.61	+	+	+	+	+	NA	NA	NA	NA	NA	11	5986.87	3.33	0.04
4	8.10	4.31	+	NA	NA	NA	NA	NA	NA	NA	NA	NA	7	5986.90	3.37	0.03
24	-0.69	4.50	+	+	NA	+	NA	NA	NA	NA	NA	NA	9	5986.92	3.39	0.03
56	-4.72	4.36	+	+	NA	+	+	NA	NA	NA	NA	NA	10	5987.34	3.80	0.03
128	7.43	2.02	+	+	+	+	+	+	NA	NA	NA	NA	12	5988.39	4.85	0.02
5	29.13	NA	NA	+	NA	NA	NA	NA	NA	NA	NA	NA	6	5989.97	6.44	0.01
1	35.32	NA	NA	NA	NA	NA	NA	NA	NA	NA	NA	NA	5	5991.03	7.50	0.00
39	20.82	NA	+	+	NA	NA	+	NA	NA	NA	NA	NA	8	5991.31	7.77	0.00
7	26.85	NA	+	+	NA	NA	NA	NA	NA	NA	NA	NA	7	5991.73	8.19	0.00
3	33.28	NA	+	NA	NA	NA	NA	NA	NA	NA	NA	NA	6	5992.83	9.30	0.00
	**I**	**AF**	**P**	**S**	**AF:P**	**AF:S**	**P:S**	**AF:P:S**	**NA**	**NA**	**NA**	**NA**	**df**	**AIC**	**delta**	**weight**
6	17.95	+	NA	+	NA	NA	NA	NA	NA	NA	NA	NA	16	5981.87	0.00	0.29
16	23.27	+	+	+	+	NA	NA	NA	NA	NA	NA	NA	27	5983.03	1.15	0.16
8	13.98	+	+	+	NA	NA	NA	NA	NA	NA	NA	NA	17	5983.17	1.30	0.15
40	9.40	+	+	+	NA	NA	+	NA	NA	NA	NA	NA	18	5983.49	1.62	0.13
48	18.79	+	+	+	+	NA	+	NA	NA	NA	NA	NA	28	5983.65	1.78	0.12
2	27.02	+	NA	NA	NA	NA	NA	NA	NA	NA	NA	NA	15	5984.66	2.79	0.07
4	23.67	+	+	NA	NA	NA	NA	NA	NA	NA	NA	NA	16	5986.15	4.27	0.03
12	32.48	+	+	NA	+	NA	NA	NA	NA	NA	NA	NA	26	5986.25	4.38	0.03
5	29.13	NA	NA	+	NA	NA	NA	NA	NA	NA	NA	NA	6	5989.97	8.10	0.01
1	35.32	NA	NA	NA	NA	NA	NA	NA	NA	NA	NA	NA	5	5991.03	9.16	0.00
39	20.82	NA	+	+	NA	NA	+	NA	NA	NA	NA	NA	8	5991.31	9.44	0.00
7	26.85	NA	+	+	NA	NA	NA	NA	NA	NA	NA	NA	7	5991.73	9.86	0.00
22	19.24	+	NA	+	NA	+	NA	NA	NA	NA	NA	NA	26	5991.90	10.02	0.00
3	33.28	NA	+	NA	NA	NA	NA	NA	NA	NA	NA	NA	6	5992.83	10.96	0.00
24	14.87	+	+	+	NA	+	NA	NA	NA	NA	NA	NA	27	5992.86	10.98	0.00
56	11.09	+	+	+	NA	+	+	NA	NA	NA	NA	NA	28	5993.79	11.91	0.00
32	24.95	+	+	+	+	+	NA	NA	NA	NA	NA	NA	37	5999.25	17.37	0.00
64	21.30	+	+	+	+	+	+	NA	NA	NA	NA	NA	38	6000.53	18.65	0.00
128	22.64	+	+	+	+	+	+	+	NA	NA	NA	NA	46	6011.27	29.39	0.00
	**I**	**AT**	**P**	**S**	**AT:P**	**AT:S**	**P:S**	**AT:P:S**	**NA**	**NA**	**NA**	**NA**	**df**	**AIC**	**delta**	**weight**
6	-40.44	9.57	NA	+	NA	NA	NA	NA	NA	NA	NA	NA	7	5977.06	0.00	0.21
22	-29.66	8.03	NA	+	NA	+	NA	NA	NA	NA	NA	NA	8	5977.80	0.73	0.14
8	-44.34	9.64	+	+	NA	NA	NA	NA	NA	NA	NA	NA	8	5978.45	1.38	0.10
40	-48.48	9.47	+	+	NA	NA	+	NA	NA	NA	NA	NA	9	5978.54	1.48	0.10
24	-33.37	8.03	+	+	NA	+	NA	NA	NA	NA	NA	NA	9	5979.05	1.99	0.08
56	-37.81	7.93	+	+	NA	+	+	NA	NA	NA	NA	NA	10	5979.26	2.20	0.07
2	-28.32	8.92	NA	NA	NA	NA	NA	NA	NA	NA	NA	NA	6	5979.37	2.31	0.07
32	-17.81	5.70	+	+	+	+	NA	NA	NA	NA	NA	NA	10	5980.06	3.00	0.05
16	-37.26	8.57	+	+	+	NA	NA	NA	NA	NA	NA	NA	9	5980.14	3.08	0.04
48	-43.36	8.74	+	+	+	NA	+	NA	NA	NA	NA	NA	10	5980.44	3.38	0.04
64	-24.72	6.05	+	+	+	+	+	NA	NA	NA	NA	NA	11	5980.66	3.60	0.03
4	-31.56	8.97	+	NA	NA	NA	NA	NA	NA	NA	NA	NA	7	5980.90	3.84	0.03
128	-16.74	4.90	+	+	+	+	+	+	NA	NA	NA	NA	12	5981.71	4.65	0.02
12	-26.54	8.23	+	NA	+	NA	NA	NA	NA	NA	NA	NA	8	5982.79	5.73	0.01
5	29.13	NA	NA	+	NA	NA	NA	NA	NA	NA	NA	NA	6	5989.97	12.91	0.00
1	35.32	NA	NA	NA	NA	NA	NA	NA	NA	NA	NA	NA	5	5991.03	13.97	0.00
39	20.82	NA	+	+	NA	NA	+	NA	NA	NA	NA	NA	8	5991.31	14.25	0.00
7	26.85	NA	+	+	NA	NA	NA	NA	NA	NA	NA	NA	7	5991.73	14.67	0.00
3	33.28	NA	+	NA	NA	NA	NA	NA	NA	NA	NA	NA	6	5992.83	15.77	0.00
	**I**	**AL**	**I(AL^2)**	**P**	**S**	**AL:P**	**AL:S**	**I(AL^2):P**	**I(AL^2):S**	**P:S**	**AL:P:S**	**I(AL^2):P:S**	**df**	**AIC**	**delta**	**weight**
**96**	**45.63**	**-15.30**	**1.55**	**+**	**+**	**+**	**NA**	**+**	**NA**	**NA**	**NA**	**NA**	**11**	**5974.56**	**0.00**	**0.09**
352	41.66	-15.71	1.58	+	+	+	NA	+	NA	+	NA	NA	12	5974.94	0.38	0.08
224	49.41	-15.63	1.48	+	+	+	NA	+	+	NA	NA	NA	12	5975.40	0.84	0.06
480	45.36	-15.98	1.52	+	+	+	NA	+	+	+	NA	NA	13	5976.01	1.45	0.05
128	49.93	-16.33	1.57	+	+	+	+	+	NA	NA	NA	NA	12	5976.07	1.51	0.04
256	39.82	-11.38	1.13	+	+	+	+	+	+	NA	NA	NA	13	5976.25	1.70	0.04
384	45.63	-16.61	1.60	+	+	+	+	+	NA	+	NA	NA	13	5976.58	2.02	0.03
192	26.17	-4.86	0.56	+	+	+	+	NA	+	NA	NA	NA	12	5976.96	2.40	0.03
512	36.40	-11.92	1.18	+	+	+	+	+	+	+	NA	NA	14	5976.98	2.42	0.03
12	25.62	-6.35	0.93	NA	+	NA	NA	NA	NA	NA	NA	NA	8	5977.05	2.49	0.03
160	36.85	-9.49	0.93	+	+	+	NA	NA	+	NA	NA	NA	11	5977.10	2.54	0.03
1504	47.25	-16.01	1.47	+	+	+	NA	+	+	+	NA	+	14	5977.11	2.56	0.03
32	29.53	-8.05	0.94	+	+	+	NA	NA	NA	NA	NA	NA	10	5977.39	2.83	0.02
11	9.96	NA	0.45	NA	+	NA	NA	NA	NA	NA	NA	NA	7	5977.58	3.03	0.02
140	30.28	-7.16	0.92	NA	+	NA	NA	NA	+	NA	NA	NA	9	5977.90	3.34	0.02
448	22.50	-4.99	0.58	+	+	+	+	NA	+	+	NA	NA	13	5978.01	3.45	0.02
416	32.81	-9.54	0.94	+	+	+	NA	NA	+	+	NA	NA	12	5978.06	3.51	0.02
896	48.79	-17.30	1.61	+	+	+	+	+	NA	+	+	NA	14	5978.09	3.54	0.02
288	25.48	-8.17	0.95	+	+	+	NA	NA	NA	+	NA	NA	11	5978.12	3.56	0.02
88	54.56	-14.97	1.51	+	NA	+	NA	+	NA	NA	NA	NA	10	5978.13	3.57	0.02
172	19.85	-2.90	0.59	NA	+	NA	+	NA	+	NA	NA	NA	10	5978.19	3.64	0.02
16	21.63	-6.35	0.94	+	+	NA	NA	NA	NA	NA	NA	NA	9	5978.29	3.73	0.01
64	36.46	-9.92	1.01	+	+	+	+	NA	NA	NA	NA	NA	11	5978.36	3.81	0.01
272	17.67	-6.67	0.95	+	+	NA	NA	NA	NA	+	NA	NA	10	5978.48	3.92	0.01
1536	39.42	-12.62	1.20	+	+	+	+	+	+	+	NA	+	15	5978.49	3.93	0.01
44	29.88	-7.44	0.98	NA	+	NA	+	NA	NA	NA	NA	NA	9	5978.65	4.09	0.01
15	5.98	NA	0.45	+	+	NA	NA	NA	NA	NA	NA	NA	8	5978.82	4.26	0.01
1024	39.34	-12.78	1.23	+	+	+	+	+	+	+	+	NA	15	5978.82	4.27	0.01
144	26.22	-7.23	0.93	+	+	NA	NA	NA	+	NA	NA	NA	10	5978.94	4.38	0.01
240	19.80	-2.65	0.42	+	+	NA	+	+	+	NA	NA	NA	12	5978.97	4.41	0.01
139	11.84	NA	0.40	NA	+	NA	NA	NA	+	NA	NA	NA	8	5979.03	4.47	0.01
208	30.76	-7.52	0.82	+	+	NA	NA	+	+	NA	NA	NA	11	5979.18	4.62	0.01
320	32.20	-9.92	1.01	+	+	+	+	NA	NA	+	NA	NA	12	5979.23	4.68	0.01
400	22.19	-7.50	0.94	+	+	NA	NA	NA	+	+	NA	NA	11	5979.26	4.71	0.01
271	1.58	NA	0.44	+	+	NA	NA	NA	NA	+	NA	NA	9	5979.27	4.71	0.01
80	24.09	-6.37	0.86	+	+	NA	NA	+	NA	NA	NA	NA	10	5979.28	4.73	0.01
176	16.35	-3.10	0.61	+	+	NA	+	NA	+	NA	NA	NA	11	5979.35	4.79	0.01
4	34.53	-6.33	0.90	NA	NA	NA	NA	NA	NA	NA	NA	NA	7	5979.54	4.98	0.01
432	12.51	-3.41	0.63	+	+	NA	+	NA	+	+	NA	NA	12	5979.72	5.16	0.01
48	26.19	-7.61	0.99	+	+	NA	+	NA	NA	NA	NA	NA	10	5979.76	5.20	0.01
79	8.36	NA	0.37	+	+	NA	NA	+	NA	NA	NA	NA	9	5979.82	5.27	0.01
336	20.08	-6.66	0.88	+	+	NA	NA	+	NA	+	NA	NA	11	5979.83	5.27	0.01
2048	33.61	-10.20	1.02	+	+	+	+	+	+	+	+	+	16	5979.90	5.35	0.01
496	16.26	-2.97	0.46	+	+	NA	+	+	+	+	NA	NA	13	5979.91	5.35	0.01
464	26.71	-7.71	0.85	+	+	NA	NA	+	+	+	NA	NA	12	5980.00	5.44	0.01
304	22.02	-7.84	1.00	+	+	NA	+	NA	NA	+	NA	NA	11	5980.03	5.47	0.01
3	18.91	NA	0.42	NA	NA	NA	NA	NA	NA	NA	NA	NA	6	5980.06	5.50	0.01
960	22.56	-5.00	0.58	+	+	+	+	NA	+	+	+	NA	14	5980.12	5.56	0.01
143	7.72	NA	0.40	+	+	NA	NA	NA	+	NA	NA	NA	9	5980.12	5.57	0.01
112	30.38	-8.00	0.91	+	+	NA	+	+	NA	NA	NA	NA	11	5980.39	5.84	0.01
335	4.02	NA	0.38	+	+	NA	NA	+	NA	+	NA	NA	10	5980.60	6.04	0.00
207	11.30	NA	0.28	+	+	NA	NA	+	+	NA	NA	NA	10	5980.60	6.04	0.00
399	3.35	NA	0.39	+	+	NA	NA	NA	+	+	NA	NA	10	5980.68	6.13	0.00
24	38.48	-7.74	0.90	+	NA	+	NA	NA	NA	NA	NA	NA	9	5980.92	6.37	0.00
8	31.18	-6.32	0.90	+	NA	NA	NA	NA	NA	NA	NA	NA	8	5980.97	6.41	0.00
368	26.08	-8.15	0.93	+	+	NA	+	+	NA	+	NA	NA	12	5981.09	6.54	0.00
832	32.83	-9.97	1.00	+	+	+	+	NA	NA	+	+	NA	13	5981.27	6.72	0.00
7	15.56	NA	0.42	+	NA	NA	NA	NA	NA	NA	NA	NA	7	5981.48	6.92	0.00
463	7.08	NA	0.29	+	+	NA	NA	+	+	+	NA	NA	11	5981.59	7.04	0.00
1488	26.69	-7.42	0.81	+	+	NA	NA	+	+	+	NA	+	13	5981.97	7.41	0.00
1520	16.39	-2.94	0.45	+	+	NA	+	+	+	+	NA	+	14	5982.01	7.45	0.00
72	33.37	-6.34	0.84	+	NA	NA	NA	+	NA	NA	NA	NA	9	5982.42	7.87	0.00
71	17.71	NA	0.36	+	NA	NA	NA	+	NA	NA	NA	NA	8	5982.93	8.37	0.00
1487	8.55	NA	0.25	+	+	NA	NA	+	+	+	NA	+	12	5983.14	8.58	0.00
10	2.25	4.62	NA	NA	+	NA	NA	NA	NA	NA	NA	NA	7	5983.54	8.98	0.00
30	6.01	3.03	NA	+	+	+	NA	NA	NA	NA	NA	NA	9	5984.04	9.48	0.00
14	-1.69	4.70	NA	+	+	NA	NA	NA	NA	NA	NA	NA	8	5984.90	10.35	0.00
286	2.10	3.03	NA	+	+	+	NA	NA	NA	+	NA	NA	10	5984.95	10.40	0.00
270	-5.56	4.52	NA	+	+	NA	NA	NA	NA	+	NA	NA	9	5985.29	10.73	0.00
2	11.39	4.26	NA	NA	NA	NA	NA	NA	NA	NA	NA	NA	6	5985.39	10.83	0.00
42	2.99	4.49	NA	NA	+	NA	+	NA	NA	NA	NA	NA	8	5985.58	11.02	0.00
62	8.41	2.56	NA	+	+	+	+	NA	NA	NA	NA	NA	10	5985.90	11.35	0.00
22	15.45	2.87	NA	+	NA	+	NA	NA	NA	NA	NA	NA	8	5986.83	12.27	0.00
318	4.31	2.61	NA	+	+	+	+	NA	NA	+	NA	NA	11	5986.87	12.32	0.00
6	8.10	4.31	NA	+	NA	NA	NA	NA	NA	NA	NA	NA	7	5986.90	12.35	0.00
46	-0.69	4.50	NA	+	+	NA	+	NA	NA	NA	NA	NA	9	5986.92	12.37	0.00
302	-4.72	4.36	NA	+	+	NA	+	NA	NA	+	NA	NA	10	5987.34	12.78	0.00
830	7.43	2.02	NA	+	+	+	+	NA	NA	+	+	NA	12	5988.39	13.83	0.00
9	29.13	NA	NA	NA	+	NA	NA	NA	NA	NA	NA	NA	6	5989.97	15.42	0.00
1	35.32	NA	NA	NA	NA	NA	NA	NA	NA	NA	NA	NA	5	5991.03	16.48	0.00
269	20.82	NA	NA	+	+	NA	NA	NA	NA	+	NA	NA	8	5991.31	16.75	0.00
13	26.85	NA	NA	+	+	NA	NA	NA	NA	NA	NA	NA	7	5991.73	17.18	0.00
5	33.28	NA	NA	+	NA	NA	NA	NA	NA	NA	NA	NA	6	5992.83	18.28	0.00

Table S7. Linear mixed effect models selected for 12 immune parameters and 4 parasitic traits, analysed separately according to sex and roe deer populations (TF: Trois-Fontaines, CH: Chizé). The effect of different age functions (factor, linear, threshold, quadratic) was tested. All models included individual identity, the year of capture and the cohort of individuals as random effects; and were tested with and without body mass (BM) as a covariate. When the age threshold model was selected, “Age of change” represents the age at which the parameter begins to vary, and the “Parameter estimate” of the age function is the slope of the variation with age after the threshold age. Statistical significance of age or body mass function are represented by * for p=0.05, ** for p=0.01 and *** for p=0.001. R2m and R2c are the marginal and conditional variance of the model, respectively. Values are presented ± Standard Error.

					AGE FUNCTION			BODY MASS					
**IMMUNE TRAIT**	Site	Sex	Best model selected	Age of change	Parameter estimate ±SE	t-value		Parameter estimate ±SE	t-value		r^2m	r^2c	Predicted value at 2 years
**INNATE TRAITS**													
**Neutrophil count**	TF	♂	constant	-	-	-	-	-	-	-	0.00	0.51	6.25 ±0.29
		♀	age factor	-	-	-	-	-	-	-	0.10	0.76	6.52 ±0.15
	CH	♂	constant	-	-	-	-	-	-	-	0.00	0.52	5.34 ±0.34
		♀	age threshold	8	0.43 ±0.14	3.00	**	-	-	-	0.05	0.27	5.14 ±0.18
**Monocyte count**	TF	♂	constant	-	-	-	-	-	-	-	0.00	0.46	0.30 ±0.10
		♀	constant	-	-	-	-	-	-	-	0.00	0.45	0.31 ±0.10
	CH	♂	constant	-	-	-	-	-			0.00	0.35	0.29 ±0.07
		♀	constant	-	-	-	-	-	-	-	0.00	0.59	0.30 ±0.10
**Eosinophil count**	TF	♂	constant	-	-	-	-	-	-	-	0.00	0.21	0.07 ±0.01
		♀	constant	-	-	-	-	-	-	-	0.00	0.14	0.12 ±0.02
	CH	♂	constant	-	-			-			0.00	0.08	0.10 ±0.02
		♀	constant	-	-	-	-	-	-	-	0.00	0.14	0.11 ±0.01
**Basophil count**	TF	♂	constant	-	-	-	-	-	-	-	0.00	0.41	0.07 ±0.02
		♀	constant	-	-	-	-	-	-	-	0.00	0.28	0.07 ±0.02
	CH	♂	BM	-	-	-	-	0.01 ±0.003	2.56	*	0.04	0.22	0.07 ±0.02
		♀	age threshold	5	0.01 ±0.004	2.16	*	-	-	-	0.03	0.19	0.08 ±0.01
**Hemagglutination**	TF	♂	constant	-	-	-	-	-	-	-	0.00	0.31	4.03 ±0.33
		♀	constant	-	-	-	-	-	-	-	0.00	0.36	4.03 ±0.25
	CH	♂	constant	-	-	-	-	-	-	-	0.00	0.54	4.26 ±0.35
		♀	age threshold	10	-0.43 ±0.20	-2.17	*	-	-	-	0.02	0.57	3.98 ±0.36
**Hemolysis**	TF	♂	constant	-	-	-	-	-	-	-	0.00	0.51	2.24 ±0.41
		♀	age factor	-	-	-	-	-	-	-	0.07	0.62	2.35 ±0.37
	CH	♂	constant	-	-	-	-	-	-	-	0.00	0.76	2.10 ±0.40
		♀	constant	-	-	-	-	-	-	-	0.00	0.62	1.97 ±0.38
**INFLAMMATORY MARKERS**													
**Beta-globulin**	TF	♂	age linear	2	0.32 ±0.08	4.17	**	-	-	-	0.09	0.81	6.20 ±0.54
		♀	age linear + BM	2	0.27 ±0.03	7.92	***	-0.14 ±0.04	-2.98	**	0.19	0.41	5.73 ±0.36
	CH	♂	age linear	2	0.20 ±0.10	2.06	*	-	-	-	0.03	0.25	7.12 ±0.51
		♀	age linear	2	0.19 ±0.05	4.00	***	-	-	-	0.08	0.44	6.41 ±0.41

**Alpha-1-globulin**	TF	♂	BM	-	-	-	-	-0.06 ±0.02	-3.14	**	0.04	0.55	3.35 ±0.20
		♀	age linear + BM	2	0.04 ±0.01	3.39	***	-0.07 ±0.02	-3.62	***	0.06	0.55	3.11 ±0.19
	CH	♂	constant	-	-	-	-	-	-	-	0.00	0.40	3.24 ±0.16
		♀	age linear	2	0.03 ±0.02	1.84	*	-	-	-	0.02	0.43	2.94 ±0.15
**Alpha-2-globulin**	TF	♂	constant	-	-	-	-	-	-	-	0.00	0.30	5.63 ±0.30
		♀	constant	-	-	-	-	-	-	-	0.00	0.26	5.84 ±0.34
	CH	♂	constant	-	-	-	-	-	-	-	0.00	0.29	5.66 ±0.33
		♀	constant	-	-	-	-	-	-	-	0.00	0.36	5.88 ±0.29
**Haptoglobin**	TF	♂	age threshold	9	0.43 ±0.12	3.46	***	-	-	-	0.07	0.52	0.34 ±0.10
		♀	age threshold	9	0.47 ±0.07	7.04	***	-	-	-	0.21	0.24	0.15 ±0.06
	CH	♂	constant	-	-	-	-	-	-	-	0.00	0.05	0.69 ±0.14
		♀	age factor	-	-	-	-	-	-	-	0.14	0.16	0.30 ±0.11
**ADAPTIVE TRAITS**													
**Gamma-globulin**	TF	♂	age linear + BM	2	0.43 ±0.11	3.88	***	-0.35 ±0.12	-2.98	**	0.10	0.49	13.86 ±0.90
		♀	age linear + BM	2	0.56 ±0.10	5.71	***	-0.29 ±0.12	-2.30	*	0.15	0.55	13.54 ±0.93
	CH	♂	constant	-	-	-	-	-	-	-	0.00	0.74	18.55 ±1.66
		♀	age linear	2	0.42 ±0.12	3.37	***	-	-	-	0.04	0.70	18.43 ±1.57
**Lymphocyte count**	TF	♂	constant	-	-	-	-	-	-	-	0.00	0.41	2.25 ±0.14
		♀	BM	-	-	-	-	-0.07 ±0.03	-2.20	*	0.03	0.38	2.42 ±0.13
	CH	♂	age linear	2	-0.07 ±0.03	-2.20	*	-	-	-	0.03	0.24	1.90 ±0.17
		♀	age linear	2	-0.06 ±0.02	-2.66	**	-	-	-	0.04	0.37	1.89 ±0.15

					AGE FUNCTION			BODY MASS					
**PARASITIC TRAIT**	Site	Sex	Best model selected	Age of change	Parameter estimate ±SE	t-value		Parameter estimate ±SE	t-value		r^2m	r^2c	Predicted value at 2 years
**Gastro-intestinal strongyles**	TF	♂	age threshold + BM	9	43.82 ±10.70	4.10	***	-6.74 ±2.40	-2.81	**	0.15	0.63	34.99 ±10.81
		♀	age threshold	11	42.47 ±13.22	3.21	**	-	-	-	0.08	0.39	15.05 ±4.93
	CH	♂	constant	-	-	-	-	-	-	-	0.00	0.33	34.48 ±10.61
		♀	age linear + BM	2	6.54 ±2.91	2.25	*	-4.70 ±2.35	-2.00	*	0.03	0.95	22.01 ±16.35
***Trichuris* sp.**	TF	♂	age threshold + BM	9	52.44 ±6.65	7.88	***	-2.98 ±1.31	-2.27	*	0.37	0.45	7.97 ±4.75
		♀	age threshold + BM	11	59.99 ±12.21	4.91	***	-3.52 ±1.26	-2.80	**	0.21	0.7	7.35 ±3.40
	CH	♂	age threshold	6	107.34 ±26.74	4.01	***	-	-	-	0.15	0.19	81.42 ±32.81
		♀	constant	-	-	-	-	-	-	-	0.00	0.53	86.40 ±29.43
**Protostrongylids**	TF	♂	age factor + BM	-	-	-	-	-0.35 ±0.14	-2.55	*	0.17	0.83	0.17 ±0.02
		♀	age threshold	11	10.17 ±1.57	6.47	***	-	-	-	0.23	0.23	0.41 ±0.37
	CH	♂	constant	-	-	-	-	-	-	-	0.00	0.00	1.40 ±0.74
		♀	constant	-	-	-	-	-	-	-	0.00	0.06	0.38 ±0.18
**Coccidia**	TF	♂	constant	-	-	-	-	-	-	-	0.00	0.99	233.38 ±194.70
		♀	constant	-	-	-	-	-	-	-	0.00	0.00	244.10 ±202.30
	CH	♂	constant	-	-	-	-	-	-	-	0.00	0.25	26.07 ±13.75
		♀	constant	-	-	-	-	-	-	-	0.00	0.96	27.48 ±8.86

Table S8. Linear mixed models describing the relationships between the 12 immune traits and the 4 parasitic traits of the study. Each immune trait was analysed as a function of parasite load, population and the interaction parasite load*population, considering 4 different parasite groups. Models included individual identity as a random effect. The "estimate" for the population or the parasitic trait is the slope of the variation. Statistical significance is represented by . for p=0.1, * for p=0.05, ** for p=0.01 and *** for p=0.001. Values are presented ± 1 SE.

**GASTRO-INTESTINAL STRONGYLES**	**POPULATION**			**GI STRONGYLES**			**POPULATION*GI STRONGYLES**		
	estimate ±SE	t-value	p	estimate ±SE	t-value	p	estimate ±SE	t-value	p
**Neutrophil count**	-1.08 ±0.27	-3.89	***	-6.47 E-06 ±0.002	-0.003	-	-0.001 ±0.003	-0.44	-
**Monocyte count**	-0.03 ±0.03	-0.95	-	9.04 E-05 ±3.22 E-04	0.28	-	-3.82 E-05 ±4.00 E-04	-0.10	-
**Basophil count**	0.001 ±0.01	0.09	-	3.79 E-05 ±1.12 E-04	0.34	-	6.08 E-05 ±1.39 E-04	0.44	-
**Eosinophil count**	0.001 ±0.01	0.09	-	-3.14 E-04 ±1.38 E-04	-2.28	*	2.99 E-04 ±1.72 E-04	1.74	-
**Lymphocyte count**	-0.74 ±0.10	-7.62	***	-7.22 E-04 ±8.78 E-04	-0.82	-	-2.59 E-04 ±0.001	-0.24	-
**Alpha1-globulin**	-0.04 ±0.06	-0.71	-	0.001 ±0.001	2.35	*	-0.002 ±0.0007	-2.09	*
**Alpha2-globulin**	0.03 ±0.14	0.8	-	0.001 ±0.001	0.55	-	-0.002 ±0.002	-0.98	-
**Beta-globulin**	0.89 ±0.21	4.25	***	0.01 ±0.001	2.79	**	-0.004 ±0.002	-1.71	.
**Gamma-globulin**	4.84 ±0.47	10.26	***	0.01 ±0.004	1.36	-	-0.01 ±0.01	-1.53	-
**Haptoglobin**	0.16 ±0.1	1.63	-	0.002 ±0.001	2.09	*	-0.001 ±0.001	-0.73	-
**Hemagglutination**	0.14 ±0.12	1.17	-	2.95 E-04 ±1.19 E-03	0.25	-	-1.62 E-03 ±1.48 E-03	-1.10	-
**Hemolysis**	-0.11 ±0.10	-1.16	-	-3.49 E-04 ±9.65 E-04	-0.36	-	-8.30 E-05 ±1.19 E-03	-0.07	-

TRICHURIS SP.	**POPULATION**			***TRICHURIS SP.***			**POPULATION*_TRICHURIS SP._**		
	estimates ±SE	t-value	p	estimates ±SE	t-value	p	estimates ±SE	t-value	p
**Neutrophil count**	-0.97 ±0.27	-3.59	***	0.003 ±0.003	1.13	-	-0.005 ±0.003	-1.46	-
**Monocyte count**	-0.04 ±0.03	-1.15	-	-0.001 ±0.0004	-1.27	-	0.0005 ±0.0005	1.18	-
**Basophil count**	0.001 ±0.01	0.11	-	2.02 E-05 ±1.56 E-04	0.13	-	5.53 E-06 ±1.60 E-04	0.04	-
**Eosinophil count**	0.01 ±0.01	0.84	-	3.67 E-05 ±1.94 E-04	0.19	-	-5.18 E-05 ±1.99 E-04	-0.26	-
**Lymphocyte count**	-0.69 ±0.10	-7.30	***	-3.34 E-05 ±1.22 E-03	-0.03	-	-6.40 E-04 ±1.25 E-03	-0.51	-
**Alpha1-globulin**	-0.03 ±0.06	-0.53	-	0.002 ±0.0001	2.34	*	-0.002 ±0.0008	-2.67	**
**Alpha2-globulin**	-0.01 ±0.14	-0.09	-	-0.002 ±0.002	-1.04	-	-0.002 ±0.002	0.86	-
**Beta-globulin**	0.93 ±0.21	4.45	***	0.01 ±0.003	3.55	***	-0.01 ±0.003	-3.67	***
**Gamma-globulin**	4.54 ±0.46	9.78	***	0.01 ±0.01	2.28	*	-0.01 ±0.01	-1.86	.
**Haptoglobin**	0.21 ±0.09	2.24	*	0.01 ±0.001	4.82	***	-0.01 ±0.001	-4.90	***
**Hemagglutination**	0.14 ±0.12	1.2	-	0.002 ±0.002	1.05	-	-0.002 ±0.002	-1.22	-
**Hemolysis**	-0.18 ±0.09	-1.97	*	-0.001 ±0.001	-0.75	-	0.002 ±0.001	1.2	-

PROTOSTRONGYLIDS	POPULATION			PROTOSTRONGYLIDS			POPULATION*PROTOSTRONGYLIDS		
	estimates ±SE	t-value	p	estimates ±SE	t-value	p	estimates ±SE	t-value	p
**Neutrophil count**	-1,10 ±0,26	-4,16	***	0,01 ±0,03	0,26	-	-0,02 ±0,04	-0,40	-
**Monocyte count**	-0,03 ±0,03	-0,87	-	0,003 ±0,005	0,81	-	-0,01 ±0,01	-0,90	-
**Basophil count**	0,002 ±0,02	3,83	**	-8,42 E04 ±0,002	-0,52	-	7,19 E04 ±0,002	0,33	-
**Eosinophil count**	0,008 ±0,014	0,56	-	-0,002 ±0,002	-0,83	-	0,003 ±0,003	1,01	-
**Lymphocyte count**	-0,76 ±0,10	-8,31	***	-0,02 ±0,01	-1,84	.	0,02 ±0,02	0,93	-
**Alpha1-globulin**	-0,10 ±0,06	-1,62	-	-0,002 ±0,008	-0,27	-	0,01 ±0,01	1,08	-
**Alpha2-globulin**	-0,02 ±0,13	-0,14	-	-0,02 ±0,02	-1,19	-	0,003 ±0,03	0,12	-
**Beta-globulin**	0,76 ±0,20	3,79	***	0,004 ±0,003	0,14	-	0,04 ±0,04	1,02	-
**Gamma-globulin**	4,55 ±0,45	10,1	***	-0,05 ±0,06	-0,72	-	0,08 ±0,08	0,99	-
**Haptoglobin**	0,14 ±0,09	1,61	-	0,01 ±0,01	0,41	-	0,002 ±0,02	0,12	-
**Hemagglutination**	0,11 ±0,11	1,03	-	0,001 ±0,017	0,06	-	-0,03 ±0,02	-1,34	-
**Hemolysis**	-0,12 ±0,09	-1,34	-	-0,02 ±0,01	-1,14	-	0,01 ±0,02	0,42	-

COCCIDIA	POPULATION			COCCIDIA			POPULATION*COCCIDIA		
	estimates ±SE	t-value	p	estimates ±SE	t-value	p	estimates ±SE	t-value	p
**Neutrophil count**	-0,92 ±0,27	-3,22	**	0,04 ±0,06	0,66	-	-0,16 ±0,10	-1,63	-
**Monocyte count**	-0,05 ±0,03	-1,54	-	-0,01 ±0,01	-1,61	-	0,02 ±0,01	1,17	-
**Basophil count**	-0,01 ±0,01	-0,92	-	-0,001 ±0,003	-0,45	-	0,01 ±0,005	2,44	*
**Eosinophil count**	-0,002 ±0,02	-0,12	-	-0,01 ±0,004	-2,06	*	0,009 ±0,006	1,43	-
**Lymphocyte count**	-0,78 ±0,10	-7,59	***	-0,05 ±0,02	-1,92	.	0,02 ±0,04	0,48	-
**Alpha1-globulin**	-0,11 ±0,07	-1,69	.	0,01 ±0,02	0,66	-	0,02 ±0,02	0,97	-
**Alpha2-globulin**	0,14 ±0,15	0,93	-	0,09 ±0,0	2,17	*	-0,11 ±0,06	-1,88	-
**Beta-globulin**	0,78 ±0,20	3,9	***	-2,38 E-05 ±5,64 E-05	-0,42	-	2,71 E-05 ±5,74 E-05	0,47	-
**Gamma-globulin**	4,27 ±0,50	8,53	***	-0,04 ±0,12	-0,37	-	0,28 ±0,18	1,53	-
**Haptoglobin**	0,10 ±0,10	1,01	-	-0,01 ±0,03	-0,45	-	0,03 ±0,04	0,85	-
**Hemagglutination**	0,08 ±0,13	0,66	-	0,01 ±0,03	0,3	-	0,006 ±0,05	0,12	-
**Hemolysis**	-0,04 ±0,10	-0,41	-	0,07 ±0,03	2,76	**	-0,05 ±0,04	-1,12	-

Table S9. Set of models fitted to assess senescence patterns of immune traits including a fixed effect of the longevity of individuals, in males and females at Trois-Fontaines, and in females at Chizé. We do not have enough data on Chizé males to test these models. Model comparison was based on AIC, k is the number of parameters, ΔAIC is the difference of AIC between the candidate model and the model having the lowest AIC, and wi the AIC weight of each model. All models included individual identity, the year of capture and the cohort of individuals as random effects. The model with the lowest AIC is shown in italics; and models with very similar explanatory power (ΔAIC < 2), which included fewer terms, are shown in bold.

	TROIS FONTAINES MALES				TROIS FONTAINES FEMALES				CHIZE FEMALES			
**Neutrophil count**	k	AIC	DAIC	wi	k	AIC	DAIC	wi	k	AIC	DAIC	wi
nul	5	**340.60**	**0.67**	**0.14**	5	483.16	6.72	0.03	5	171.61	10.19	0.01
longevity	6	342.53	2.59	0.05	6	485.12	8.68	0.01	6	168.15	6.73	0.03
BM	6	342.02	2.08	0.06	6	485.12	8.67	0.01	6	173.49	12.07	0.00
longevity + BM	7	343.94	4.00	0.02	7	487.09	10.64	0.00	7	169.29	7.86	0.01
age (factor)	15	350.60	10.67	0.00	15	***476.45***	***0.00***	***0.09***	14	166.90	5.47	0.00
age (factor) + longevity	16	349.92	9.99	0.00	16	476.74	0.29	0.05	15	164.87	3.45	0.00
age (factor) + BM	16	352.45	12.52	0.00	16	478.17	1.72	0.02	15	168.89	7.46	0.00
age (factor) + longevity + BM	17	351.77	11.83	0.00	17	478.40	1.95	0.01	16	166.73	5.30	0.00
age (linear)	6	340.18	0.25	0.15	6	479.43	2.98	0.20	6	170.05	8.63	0.01
age (linear) + longevity	7	*339.93*	*0.00*	*0.14*	7	479.34	2.89	0.18	7	169.06	7.64	0.01
age (linear) + BM	7	341.79	1.86	0.05	7	480.91	4.46	0.08	7	171.89	10.47	0.00
age (linear) + longevity + BM	8	341.66	1.72	0.05	8	480.80	4.35	0.07	8	170.44	9.02	0.00
age (quadratic)	7	340.64	0.71	0.10	7	481.04	4.59	0.08	7	171.89	10.47	0.00
age (quadratic) + longevity	8	340.80	0.87	0.07	8	480.83	4.38	0.07	8	170.92	9.50	0.00
age (quadratic) + BM	8	342.64	2.71	0.03	8	482.22	5.77	0.04	8	173.73	12.31	0.00
age (quadratic) + longevity + BM	9	342.80	2.87	0.02	9	481.92	5.48	0.03	9	172.24	10.82	0.00
age (threshold)	6	341.82	1.89	0.07	6	485.14	8.70	0.01	6	164.35	2.93	0.19
age (threshold) + longevity	7	343.01	3.08	0.03	7	487.10	10.65	0.00	7	***161.42***	***0.00***	***0.54***
age (threshold) + BM	7	342.99	3.06	0.03	7	487.10	10.66	0.00	7	166.33	4.90	0.05
age (threshold) + longevity + BM	8	343.97	4.04	0.01	8	489.07	12.62	0.00	8	163.08	1.66	0.14
**Eosinophil count**	k	AIC	DAIC	wi	k	AIC	DAIC	wi	k	AIC	DAIC	wi
nul	5	***-115.72***	***0.00***	***0.28***	5	**-117.25**	**1.91**	**0.11**	5	***-36.65***	***0.00***	***0.31***
longevity	6	-113.73	1.99	0.09	6	*-119.16*	*0.00*	*0.26*	6	-34.70	1.96	0.08
BM	6	-114.43	1.30	0.12	6	-115.49	3.68	0.04	6	-34.93	1.73	0.09
longevity + BM	7	-112.43	3.29	0.04	7	-117.85	1.31	0.11	7	-32.96	3.70	0.02
age (factor)	15	-107.01	8.71	0.00	15	-106.59	12.57	0.00	14	-25.16	11.50	0.00
age (factor) + longevity	16	-105.19	10.54	0.00	16	-106.91	12.25	0.00	15	-23.16	13.50	0.00
age (factor) + BM	16	-106.12	9.60	0.00	16	-104.92	14.24	0.00	15	-23.16	13.50	0.00
age (factor) + longevity + BM	17	-104.32	11.40	0.00	17	-105.24	13.93	0.00	16	-21.16	15.49	0.00
age (linear)	6	-114.13	1.59	0.11	6	-115.98	3.18	0.05	6	-34.65	2.00	0.08
age (linear) + longevity	7	-112.28	3.45	0.03	7	-117.35	1.82	0.09	7	-32.71	3.94	0.02
age (linear) + BM	7	-112.96	2.77	0.05	7	-114.39	4.77	0.02	7	-32.93	3.73	0.02
age (linear) + longevity + BM	8	-111.17	4.55	0.02	8	-116.02	3.15	0.04	8	-30.96	5.69	0.00
age (quadratic)	7	-112.41	3.31	0.04	7	-114.06	5.10	0.02	7	-33.87	2.79	0.03
age (quadratic) + longevity	8	-110.52	5.20	0.01	8	-115.94	3.22	0.04	8	-31.91	4.75	0.01
age (quadratic) + BM	8	-112.07	3.65	0.02	8	-112.41	6.75	0.01	8	-32.16	4.49	0.01
age (quadratic) + longevity + BM	9	-110.22	5.50	0.01	9	-114.43	4.73	0.01	9	-30.18	6.47	0.00
age (threshold)	6	-113.93	1.80	0.10	6	-116.15	3.01	0.06	6	-36.54	0.12	0.20
age (threshold) + longevity	7	-111.94	3.79	0.03	7	-117.28	1.88	0.09	7	-34.74	1.91	0.05
age (threshold) + BM	7	-112.52	3.21	0.04	7	-114.39	4.78	0.02	7	-34.72	1.93	0.05
age (threshold) + longevity + BM	8	-110.52	5.21	0.01	8	-115.93	3.23	0.04	8	-32.91	3.74	0.01
**Basophil count**	k	AIC	DAIC	wi	k	AIC	DAIC	wi	k	AIC	DAIC	wi
nul	5	***-94.96***	***0.00***	***0.21***	5	**-241.77**	**0.52**	**0.16**	5	**-54.71**	**1.79**	**0.18**
longevity	6	-93.35	1.60	0.08	6	-240.59	1.71	0.08	6	-53.23	3.27	0.06
BM	6	-93.00	1.95	0.07	6	-240.18	2.11	0.06	6	-53.06	3.44	0.06
longevity + BM	7	-91.40	3.56	0.02	7	-239.15	3.14	0.03	7	-51.65	4.85	0.02
age (factor)	15	-81.48	13.48	0.00	15	-229.73	12.56	0.00	14	-50.54	5.96	0.00
age (factor) + longevity	16	-81.48	13.48	0.00	16	-227.79	14.50	0.00	15	-48.65	7.85	0.00
age (factor) + BM	16	-79.62	15.34	0.00	16	-229.44	12.86	0.00	15	-48.57	7.93	0.00
age (factor) + longevity + BM	17	-79.60	15.36	0.00	17	-227.46	14.83	0.00	16	-46.65	9.84	0.00
age (linear)	6	-93.95	1.01	0.11	6	-239.98	2.31	0.06	6	-54.49	2.01	0.11
age (linear) + longevity	7	-94.31	0.65	0.10	7	-238.60	3.69	0.02	7	-52.50	4.00	0.03
age (linear) + BM	7	-92.05	2.91	0.03	7	-238.45	3.84	0.02	7	-52.83	3.66	0.03
age (linear) + longevity + BM	8	-92.46	2.50	0.03	8	-237.16	5.13	0.01	8	-50.83	5.66	0.01
age (quadratic)	7	-92.15	2.80	0.04	7	*-242.29*	*0.00*	*0.15*	7	*-56.50*	*0.00*	*0.20*
age (quadratic) + longevity	8	-92.42	2.54	0.03	8	-240.34	1.95	0.05	8	-54.52	1.97	0.05
age (quadratic) + BM	8	-90.54	4.41	0.01	8	-241.23	1.06	0.08	8	-54.73	1.76	0.05
age (quadratic) + longevity + BM	9	-90.83	4.13	0.01	9	-239.32	2.97	0.02	9	-52.74	3.75	0.01
age (threshold)	6	-93.81	1.15	0.10	6	-241.35	0.94	0.11	6	-54.32	2.18	0.11
age (threshold) + longevity	7	-94.07	0.89	0.09	7	-240.86	1.44	0.07	7	-53.41	3.09	0.04
age (threshold) + BM	7	-91.84	3.11	0.03	7	-239.82	2.47	0.04	7	-52.73	3.77	0.03
age (threshold) + longevity + BM	8	-92.08	2.88	0.03	8	-239.60	2.69	0.03	8	-51.95	4.55	0.01

**Monocyte count**	k	AIC	DAIC	wi	k	AIC	DAIC	wi	k	AIC	DAIC	wi
nul	5	***75.23***	***0.00***	***0.28***	5	***53.02***	***0.00***	***0.23***	5	***13.75***	***0.00***	***0.36***
longevity	6	77.23	2.00	0.09	6	55.01	1.98	0.07	6	15.35	1.60	0.11
BM	6	76.96	1.73	0.10	6	54.60	1.58	0.09	6	15.29	1.55	0.12
longevity + BM	7	78.96	3.73	0.03	7	56.57	3.54	0.03	7	16.58	2.84	0.04
age (factor)	15	87.33	12.09	0.00	15	57.63	4.61	0.00	14	17.59	3.85	0.00
age (factor) + longevity	16	88.84	13.61	0.00	16	58.47	5.44	0.00	15	18.49	4.74	0.00
age (factor) + BM	16	89.32	14.09	0.00	16	59.30	6.27	0.00	15	19.58	5.83	0.00
age (factor) + longevity + BM	17	90.84	15.61	0.00	17	60.18	7.15	0.00	16	20.44	6.70	0.00
age (linear)	6	77.01	1.77	0.10	6	54.24	1.22	0.11	6	15.73	1.98	0.09
age (linear) + longevity	7	78.94	3.71	0.03	7	56.10	3.08	0.04	7	17.13	3.39	0.03
age (linear) + BM	7	78.75	3.52	0.03	7	55.76	2.73	0.04	7	17.27	3.53	0.03
age (linear) + longevity + BM	8	80.66	5.43	0.01	8	57.66	4.63	0.01	8	18.22	4.48	0.01
age (quadratic)	7	77.90	2.67	0.05	7	55.60	2.58	0.05	7	17.72	3.97	0.02
age (quadratic) + longevity	8	79.74	4.50	0.02	8	57.57	4.55	0.01	8	19.13	5.38	0.01
age (quadratic) + BM	8	79.89	4.65	0.01	8	57.17	4.15	0.02	8	19.26	5.52	0.01
age (quadratic) + longevity + BM	9	81.72	6.49	0.00	9	59.16	6.13	0.01	9	20.22	6.48	0.00
age (threshold)	6	76.20	0.97	0.14	6	53.58	0.55	0.15	6	15.64	1.90	0.10
age (threshold) + longevity	7	77.99	2.76	0.05	7	55.54	2.51	0.05	7	17.29	3.55	0.03
age (threshold) + BM	7	78.10	2.87	0.05	7	55.13	2.11	0.06	7	17.22	3.48	0.03
age (threshold) + longevity + BM	8	79.90	4.66	0.01	8	57.11	4.09	0.02	8	18.57	4.82	0.01
**Lymphocyte count**	k	AIC	DAIC	wi	k	AIC	DAIC	wi	k	AIC	DAIC	wi
nul	5	***226.82***	***0.00***	***0.25***	5	***272.35***	***0.00***	***0.20***	5	***89.75***	***0.00***	***0.24***
longevity	6	228.63	1.81	0.09	6	273.19	0.84	0.11	6	90.04	0.29	0.15
BM	6	227.54	0.72	0.15	6	274.34	1.99	0.06	6	90.20	0.46	0.13
longevity + BM	7	229.37	2.55	0.05	7	275.13	2.78	0.04	7	90.74	1.00	0.07
age (factor)	15	236.94	10.12	0.00	15	280.33	7.98	0.00	14	104.28	14.54	0.00
age (factor) + longevity	16	238.93	12.11	0.00	16	282.34	9.99	0.00	15	102.37	12.63	0.00
age (factor) + BM	16	238.69	11.87	0.00	16	282.00	9.65	0.00	15	103.68	13.94	0.00
age (factor) + longevity + BM	17	240.66	13.85	0.00	17	283.85	11.50	0.00	16	102.09	12.35	0.00
age (linear)	6	228.46	1.64	0.09	6	273.21	0.86	0.11	6	91.65	1.91	0.06
age (linear) + longevity	7	230.45	3.63	0.03	7	274.86	2.51	0.04	7	90.36	0.61	0.08
age (linear) + BM	7	229.29	2.47	0.05	7	275.14	2.79	0.04	7	92.13	2.38	0.03
age (linear) + longevity + BM	8	231.27	4.45	0.01	8	276.78	4.43	0.01	8	91.31	1.57	0.03
age (quadratic)	7	230.44	3.62	0.03	7	273.15	0.80	0.10	7	93.65	3.90	0.02
age (quadratic) + longevity	8	232.42	5.60	0.01	8	274.98	2.63	0.03	8	92.28	2.54	0.02
age (quadratic) + BM	8	231.10	4.28	0.02	8	275.08	2.73	0.03	8	94.11	4.37	0.01
age (quadratic) + longevity + BM	9	233.09	6.27	0.00	9	276.89	4.54	0.01	9	93.30	3.56	0.01
age (threshold)	6	228.32	1.50	0.10	6	273.21	0.86	0.11	6	91.49	1.74	0.07
age (threshold) + longevity	7	230.32	3.50	0.03	7	274.85	2.50	0.04	7	91.91	2.17	0.04
age (threshold) + BM	7	228.80	1.98	0.06	7	275.14	2.79	0.04	7	91.94	2.20	0.04
age (threshold) + longevity + BM	8	230.78	3.96	0.02	8	276.77	4.42	0.01	8	92.60	2.86	0.02
**Alpha1-globulin**	k	AIC	DAIC	wi	k	AIC	DAIC	wi	k	AIC	DAIC	wi
nul	5	178.43	4.16	0.05	5	205.46	10.36	0.00	5	82.01	10.09	0.00
longevity	6	180.11	5.84	0.02	6	206.29	11.19	0.00	6	83.11	11.19	0.00
BM	6	***174.27***	***0.00***	***0.34***	6	203.19	8.09	0.01	6	76.43	4.50	0.05
longevity + BM	7	175.90	1.63	0.13	7	203.08	7.97	0.01	7	76.26	4.33	0.04
age (factor)	15	186.48	12.21	0.00	15	210.80	15.70	0.00	14	81.66	9.74	0.00
age (factor) + longevity	16	188.47	14.20	0.00	16	212.59	17.49	0.00	15	83.12	11.19	0.00
age (factor) + BM	16	180.70	6.43	0.00	16	208.62	13.51	0.00	15	79.34	7.41	0.00
age (factor) + longevity + BM	17	182.69	8.42	0.00	17	210.37	15.26	0.00	16	79.83	7.90	0.00
age (linear)	6	180.13	5.86	0.02	6	203.20	8.10	0.01	6	76.79	4.86	0.05
age (linear) + longevity	7	182.05	7.78	0.01	7	205.16	10.06	0.00	7	78.28	6.36	0.02
age (linear) + BM	7	175.70	1.43	0.14	7	199.41	4.30	0.03	7	**72.36**	**0.43**	**0.29**
age (linear) + longevity + BM	8	177.66	3.39	0.04	8	201.41	6.30	0.01	8	*71.92*	*0.00*	*0.24*
age (quadratic)	7	181.52	7.25	0.01	7	197.06	1.96	0.11	7	77.02	5.10	0.03
age (quadratic) + longevity	8	183.46	9.19	0.00	8	198.80	3.70	0.04	8	78.26	6.34	0.01
age (quadratic) + BM	8	177.65	3.38	0.04	8	195.36	0.26	0.21	8	73.17	1.25	0.13
age (quadratic) + longevity + BM	9	179.61	5.34	0.01	9	197.01	1.91	0.08	9	72.77	0.85	0.10
age (threshold)	6	179.56	5.30	0.02	6	197.65	2.54	0.09	6	84.01	12.09	0.00
age (threshold) + longevity	7	181.51	7.24	0.01	7	199.64	4.54	0.03	7	85.11	13.19	0.00
age (threshold) + BM	7	175.99	1.72	0.12	7	***195.10***	***0.00***	***0.28***	7	78.23	6.30	0.02
age (threshold) + longevity + BM	8	177.79	3.52	0.04	8	196.90	1.80	0.10	8	78.12	6.19	0.01

**Alpha2-globulin**	k	AIC	DAIC	wi	k	AIC	DAIC	wi	k	AIC	DAIC	wi
nul	5	**304.11**	**1.30**	**0.10**	5	**415.30**	**1.78**	**0.12**	5	***186.72***	***0.00***	***0.28***
longevity	6	305.50	2.69	0.04	6	417.27	3.75	0.04	6	188.72	2.00	0.08
BM	6	304.64	1.83	0.06	6	*413.52*	*0.00*	*0.26*	6	186.80	0.08	0.20
longevity + BM	7	305.98	3.17	0.03	7	415.47	1.95	0.09	7	188.72	2.00	0.06
age (factor)	15	307.12	4.30	0.00	15	426.64	13.12	0.00	14	197.56	10.84	0.00
age (factor) + longevity	16	306.35	3.54	0.00	16	428.55	15.03	0.00	15	199.56	12.84	0.00
age (factor) + BM	16	308.39	5.58	0.00	16	423.82	10.30	0.00	15	197.87	11.15	0.00
age (factor) + longevity + BM	17	307.72	4.90	0.00	17	425.67	12.14	0.00	16	199.81	13.09	0.00
age (linear)	6	304.60	1.78	0.06	6	417.03	3.51	0.05	6	188.72	2.00	0.08
age (linear) + longevity	7	*302.81*	*0.00*	*0.13*	7	418.88	5.36	0.02	7	190.72	4.00	0.02
age (linear) + BM	7	305.23	2.41	0.04	7	415.39	1.86	0.09	7	188.79	2.07	0.05
age (linear) + longevity + BM	8	303.65	0.84	0.07	8	417.21	3.69	0.03	8	190.71	3.99	0.01
age (quadratic)	7	306.40	3.58	0.02	7	418.95	5.43	0.02	7	190.37	3.65	0.02
age (quadratic) + longevity	8	304.79	1.97	0.04	8	420.82	7.30	0.01	8	192.36	5.64	0.01
age (quadratic) + BM	8	307.22	4.41	0.01	8	417.19	3.67	0.03	8	190.71	3.99	0.01
age (quadratic) + longevity + BM	9	305.61	2.80	0.02	9	419.05	5.53	0.01	9	192.61	5.89	0.00
age (threshold)	6	303.67	0.86	0.10	6	416.66	3.14	0.05	6	188.54	1.82	0.08
age (threshold) + longevity	7	303.62	0.80	0.09	7	418.66	5.14	0.02	7	190.51	3.79	0.02
age (threshold) + BM	7	303.54	0.72	0.09	7	414.62	1.10	0.13	7	188.69	1.97	0.06
age (threshold) + longevity + BM	8	302.97	0.16	0.10	8	416.61	3.09	0.04	8	190.67	3.95	0.01

**Haptoglobin**	k	AIC	DAIC	wi	k	AIC	DAIC	wi	k	AIC	DAIC	wi
nul	5	253.52	3.99	0.06	5	326.27	23.95	0.00	5	74.93	6.47	0.02
longevity	6	255.23	5.70	0.02	6	327.37	25.05	0.00	6	76.13	7.68	0.01
BM	6	252.96	3.43	0.07	6	327.98	25.66	0.00	6	76.91	8.45	0.00
longevity + BM	7	254.73	5.20	0.02	7	328.58	26.26	0.00	7	78.12	9.66	0.00
age (factor)	15	258.96	9.44	0.00	15	318.38	16.07	0.00	14	74.26	5.80	0.00
age (factor) + longevity	16	260.96	11.43	0.00	16	320.35	18.03	0.00	15	72.51	4.05	0.00
age (factor) + BM	16	259.62	10.09	0.00	16	319.58	17.26	0.00	15	75.36	6.90	0.00
age (factor) + longevity + BM	17	261.59	12.06	0.00	17	321.55	19.23	0.00	16	74.42	5.96	0.00
age (linear)	6	254.61	5.08	0.03	6	316.54	14.23	0.00	6	71.47	3.02	0.07
age (linear) + longevity	7	256.61	7.08	0.01	7	318.01	15.70	0.00	7	69.55	1.09	0.13
age (linear) + BM	7	254.18	4.65	0.03	7	316.73	14.41	0.00	7	72.98	4.53	0.02
age (linear) + longevity + BM	8	256.17	6.64	0.01	8	318.34	16.02	0.00	8	71.54	3.08	0.03
age (quadratic)	7	253.16	3.63	0.05	7	308.66	6.34	0.02	7	70.79	2.33	0.07
age (quadratic) + longevity	8	255.11	5.58	0.01	8	310.66	8.34	0.01	8	*68.46*	*0.00*	*0.15*
age (quadratic) + BM	8	254.89	5.36	0.02	8	310.28	7.96	0.01	8	72.70	4.24	0.02
age (quadratic) + longevity + BM	9	256.85	7.32	0.00	9	312.28	9.96	0.00	9	70.26	1.80	0.04
age (threshold)	6	***249.53***	***0.00***	***0.37***	6	***302.32***	***0.00***	***0.51***	6	**69.66**	**1.20**	**0.17**
age (threshold) + longevity	7	251.51	1.98	0.11	7	304.32	2.00	0.17	7	69.18	0.73	0.16
age (threshold) + BM	7	251.00	1.47	0.15	7	303.78	1.46	0.22	7	71.66	3.20	0.05
age (threshold) + longevity + BM	8	253.00	3.47	0.04	8	305.77	3.45	0.07	8	70.68	2.22	0.05

**Gamma-globulin**	k	AIC	DAIC	wi	k	AIC	DAIC	wi	k	AIC	DAIC	wi
nul	5	484.59	12.28	0.00	5	664.61	10.59	0.00	5	307.48	2.09	0.11
longevity	6	486.05	13.74	0.00	6	663.23	9.21	0.00	6	307.69	2.30	0.08
BM	6	478.62	6.31	0.02	6	665.98	11.96	0.00	6	308.45	3.06	0.05
longevity + BM	7	479.64	7.32	0.01	7	664.30	10.29	0.00	7	309.21	3.82	0.03
age (factor)	15	491.07	18.75	0.00	15	667.35	13.34	0.00	14	313.67	8.29	0.00
age (factor) + longevity	16	492.33	20.01	0.00	16	669.33	15.31	0.00	15	315.63	10.24	0.00
age (factor) + BM	16	478.20	5.88	0.00	16	668.22	14.20	0.00	15	315.43	10.04	0.00
age (factor) + longevity + BM	17	479.78	7.46	0.00	17	670.20	16.18	0.00	16	317.42	12.03	0.00
age (linear)	6	481.22	8.90	0.01	6	654.78	0.76	0.14	6	***305.39***	***0.00***	***0.25***
age (linear) + longevity	7	482.56	10.24	0.00	7	656.77	2.75	0.04	7	307.31	1.92	0.07
age (linear) + BM	7	***472.32***	***0.00***	***0.40***	7	654.94	0.92	0.11	7	306.25	0.86	0.11
age (linear) + longevity + BM	8	473.49	1.17	0.18	8	656.92	2.90	0.04	8	308.25	2.86	0.03
age (quadratic)	7	482.95	10.63	0.00	7	654.94	0.92	0.11	7	306.22	0.83	0.12
age (quadratic) + longevity	8	484.19	11.87	0.00	8	656.86	2.84	0.04	8	308.19	2.80	0.03
age (quadratic) + BM	8	472.86	0.55	0.24	8	655.62	1.60	0.07	8	307.27	1.88	0.05
age (quadratic) + longevity + BM	9	474.22	1.91	0.10	9	657.55	3.53	0.02	9	309.27	3.88	0.01
age (threshold)	6	483.28	10.96	0.00	6	***654.02***	***0.00***	***0.20***	6	309.42	4.03	0.03
age (threshold) + longevity	7	485.06	12.75	0.00	7	655.94	1.93	0.07	7	309.53	4.14	0.02
age (threshold) + BM	7	477.86	5.54	0.03	7	654.76	0.74	0.12	7	310.45	5.06	0.01
age (threshold) + longevity + BM	8	479.29	6.98	0.01	8	656.63	2.61	0.04	8	311.15	5.76	0.01

**Beta-globulin**	k	AIC	DAIC	wi	k	AIC	DAIC	wi	k	AIC	DAIC	wi
nul	5	398.12	4.04	0.05	5	475.24	12.41	0.00	5	**203.02**	**0.74**	**0.22**
longevity	6	400.10	6.02	0.01	6	477.03	14.20	0.00	6	204.97	2.68	0.06
BM	6	398.86	4.78	0.03	6	473.45	10.62	0.00	6	205.01	2.73	0.06
longevity + BM	7	400.86	6.78	0.01	7	474.93	12.10	0.00	7	206.90	4.62	0.02
age (factor)	15	400.27	6.18	0.00	15	480.45	17.62	0.00	14	203.94	1.66	0.00
age (factor) + longevity	16	400.99	6.91	0.00	16	482.45	19.62	0.00	15	204.01	1.73	0.00
age (factor) + BM	16	400.13	6.05	0.00	16	476.66	13.83	0.00	15	205.05	2.77	0.00
age (factor) + longevity + BM	17	400.99	6.91	0.00	17	478.66	15.83	0.00	16	205.66	3.38	0.00
age (linear)	6	***394.08***	***0.00***	***0.30***	6	467.55	4.72	0.03	6	*202.28*	*0.00*	*0.24*
age (linear) + longevity	7	395.28	1.20	0.14	7	469.53	6.70	0.01	7	203.33	1.04	0.10
age (linear) + BM	7	395.07	0.99	0.15	7	462.89	0.06	0.26	7	204.27	1.98	0.06
age (linear) + longevity + BM	8	396.19	2.11	0.07	8	464.88	2.05	0.08	8	205.30	3.01	0.02
age (quadratic)	7	396.03	1.95	0.09	7	468.08	5.25	0.02	7	204.01	1.73	0.07
age (quadratic) + longevity	8	397.19	3.11	0.04	8	470.04	7.21	0.01	8	204.90	2.62	0.03
age (quadratic) + BM	8	396.83	2.75	0.05	8	464.38	1.55	0.11	8	205.97	3.68	0.02
age (quadratic) + longevity + BM	9	398.02	3.94	0.02	9	466.33	3.50	0.03	9	206.90	4.62	0.01
age (threshold)	6	399.72	5.64	0.02	6	466.00	3.17	0.06	6	205.02	2.74	0.06
age (threshold) + longevity	7	401.65	7.57	0.01	7	467.84	5.01	0.02	7	206.97	4.68	0.02
age (threshold) + BM	7	400.84	6.76	0.01	7	***462.83***	***0.00***	***0.27***	7	207.01	4.73	0.02
age (threshold) + longevity + BM	8	402.83	8.75	0.00	8	464.66	1.82	0.09	8	208.90	6.62	0.00

**Hemagglutination**	k	AIC	DAIC	wi	k	AIC	DAIC	wi	k	AIC	DAIC	wi
nul	5	**296.65**	**0.55**	**0.17**	5	**394.98**	**0.67**	**0.13**	5	**177.97**	**0.78**	**0.15**
longevity	6	298.52	2.42	0.06	6	396.89	2.58	0.05	6	179.89	2.70	0.04
BM	6	298.64	2.54	0.05	6	394.72	0.41	0.14	6	*177.19*	*0.00*	*0.16*
longevity + BM	7	300.50	4.40	0.02	7	396.72	2.41	0.04	7	179.19	2.00	0.04
age (factor)	15	308.61	12.51	0.00	15	403.23	8.92	0.00	14	189.00	11.80	0.00
age (factor) + longevity	16	309.24	13.14	0.00	16	405.00	10.69	0.00	15	189.37	12.18	0.00
age (factor) + BM	16	310.54	14.44	0.00	16	402.71	8.39	0.00	15	188.33	11.14	0.00
age (factor) + longevity + BM	17	311.16	15.06	0.00	17	404.53	10.21	0.00	16	189.10	11.91	0.00
age (linear)	6	297.27	1.17	0.10	6	396.95	2.64	0.04	6	178.31	1.12	0.09
age (linear) + longevity	7	297.50	1.40	0.08	7	398.65	4.33	0.02	7	178.54	1.35	0.06
age (linear) + BM	7	299.25	3.15	0.03	7	396.48	2.17	0.05	7	177.29	0.10	0.11
age (linear) + longevity + BM	8	299.42	3.32	0.02	8	398.32	4.01	0.02	8	178.04	0.85	0.05
age (quadratic)	7	299.13	3.02	0.03	7	396.81	2.50	0.04	7	180.31	3.12	0.02
age (quadratic) + longevity	8	299.26	3.16	0.03	8	398.73	4.42	0.01	8	180.42	3.23	0.02
age (quadratic) + BM	8	301.12	5.02	0.01	8	397.25	2.94	0.03	8	179.23	2.04	0.03
age (quadratic) + longevity + BM	9	301.26	5.15	0.01	9	399.21	4.89	0.01	9	179.84	2.65	0.01
age (threshold)	6	*296.10*	*0.00*	*0.19*	6	394.56	0.25	0.15	6	178.84	1.65	0.07
age (threshold) + longevity	7	296.61	0.50	0.12	7	395.72	1.41	0.07	7	179.79	2.60	0.03
age (threshold) + BM	7	298.05	1.95	0.06	7	*394.31*	*0.00*	*0.15*	7	177.97	0.78	0.08
age (threshold) + longevity + BM	8	298.53	2.43	0.04	8	395.94	1.63	0.06	8	179.31	2.12	0.03

**Hemolysis**	k	AIC	DAIC	wi	k	AIC	DAIC	wi	k	AIC	DAIC	wi
nul	5	**283.08**	**0.69**	**0.18**	5	356.51	5.26	0.10	5	*147.69*	*0.10*	*0.26*
longevity	6	285.04	2.66	0.06	6	357.90	6.65	0.04	6	149.37	1.78	0.09
BM	6	284.43	2.05	0.08	6	357.87	6.62	0.04	6	149.69	2.10	0.07
longevity + BM	7	286.40	4.02	0.02	7	358.96	7.71	0.02	7	151.37	3.77	0.02
age (factor)	15	293.90	11.52	0.00	15	**352.06**	**0.81**	**0.10**	14	150.47	2.87	0.00
age (factor) + longevity	16	295.75	13.37	0.00	16	*351.25*	*0.00*	*0.11*	15	152.44	4.85	0.00
age (factor) + BM	16	293.97	11.58	0.00	16	354.01	2.76	0.03	15	151.40	3.80	0.00
age (factor) + longevity + BM	17	295.86	13.48	0.00	17	353.17	1.92	0.03	16	153.30	5.70	0.00
age (linear)	6	284.77	2.39	0.06	6	357.28	6.02	0.06	6	149.69	2.10	0.07
age (linear) + longevity	7	286.73	4.35	0.02	7	355.70	4.45	0.11	7	151.24	3.65	0.02
age (linear) + BM	7	286.12	3.73	0.03	7	358.91	7.66	0.02	7	151.69	4.10	0.02
age (linear) + longevity + BM	8	288.07	5.69	0.01	8	357.04	5.79	0.05	8	153.23	5.64	0.01
age (quadratic)	7	284.79	2.41	0.05	7	358.97	7.71	0.02	7	149.95	2.35	0.05
age (quadratic) + longevity	8	286.79	4.41	0.02	8	357.65	6.40	0.04	8	151.77	4.18	0.01
age (quadratic) + BM	8	284.65	2.27	0.05	8	360.33	9.08	0.01	8	151.94	4.35	0.01
age (quadratic) + longevity + BM	9	286.64	4.26	0.01	9	358.81	7.56	0.02	9	153.77	6.18	0.00
age (threshold)	6	*282.38*	*0.00*	*0.21*	6	357.16	5.91	0.06	6	**147.59**	**0.00**	**0.21**
age (threshold) + longevity	7	284.18	1.80	0.07	7	356.56	5.30	0.07	7	149.09	1.49	0.07
age (threshold) + BM	7	283.38	0.99	0.11	7	358.63	7.37	0.03	7	149.59	1.99	0.06
age (threshold) + longevity + BM	8	285.19	2.81	0.03	8	357.41	6.16	0.04	8	151.06	3.46	0.02

Fig. S1. **Selection of the best threshold for the "threshold model" by maximum likelihood (see Methods).** We used the deviance profiles of a continuous age model with a varying threshold and selected the age leading to the lowest deviance as the threshold age.

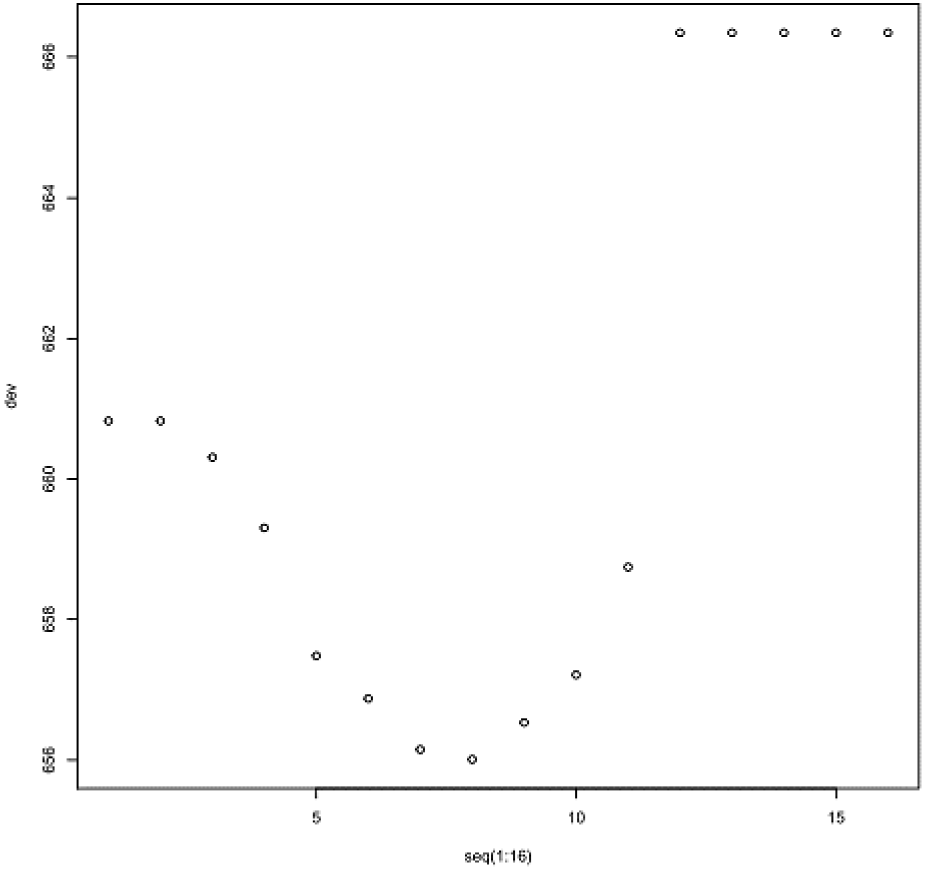

Supplement: Supplementary file 1 — Supporting Information [file 41598_2017_13686_MOESM1_ESM.pdf]
